# Transcriptome and co-expression network analyses of key genes and pathways associated with differential abscisic acid accumulation during maize seed maturation

**DOI:** 10.1186/s12870-022-03751-1

**Published:** 2022-07-22

**Authors:** Liangjie Niu, Cui Du, Wenrui Wang, Man Zhang, Wei Wang, Hui Liu, Jinghua Zhang, Xiaolin Wu

**Affiliations:** National Key Laboratory of Wheat and Maize Crop Science, College of Life Sciences, Henan Agricultural University, Zhengzhou, China

**Keywords:** Transcriptome, Abscisic acid (ABA) synthesis deficiency, Differential expressed genes (DEGs), Seed maturation, Weighted correlation network analysis (WGCNA), *Zea mays*

## Abstract

**Background:**

Currently, mechanical maize kernel harvesting has not been fully utilized in developing countries including China, partly due to the absence of suitable cultivars capable of rapid desiccation during seed maturation. The initiation of rapid desiccation during seed maturation is regulated by abscisic acid (ABA). For further characterization of ABA-regulated key genes and cellular events, it is necessary to perform transcriptome analysis of maize developing embryos. The ABA synthesis-deficient mutant (*vp5*) and normal maize (*Vp5*) seeds are suitable materials for such purpose.

**Results:**

In the present work, developing *vp5 and Vp5* embryos were compared by ABA content and transcriptome analyses. Quantitative analysis revealed the significant difference in ABA synthesis between both genotypes. From 29 days after pollination (DAP), ABA content increased rapidly in *Vp5* embryos, but decreased gradually in *vp5* embryos. At 36 DAP, ABA level in *vp5* decreased to 1/4 that of *Vp5*, suggesting that the differential ABA levels would affect seed maturation. Comparative transcriptomic analysis has found 1019 differentially expressed genes (DEGs) between both genotypes, with the most DEGs (818) at 36 DAP. Further, weighted correlation network analysis (WGCNA) revealed eight DEGs co-expression modules. Particularly, a module was negatively correlated with ABA content in *vp5* embryos. The module was mainly involved in metabolic and cellular processes, and its hub genes encoded thiamine, NPF proteins, calmodulin, metallothionein etc. Moreover, the expression of a set of key genes regulated by ABA was further verified by RT-qPCR. The results of the present work suggested that because of ABA deficiency, the *vp5* seeds maintained strong metabolic activities and lacked dormancy initiation during seed maturation.

**Conclusion:**

Transcriptome and WGCNA analyses revealed significant ABA-related changes in metabolic pathways and DEGs between *vp5* and *Vp5* during seed maturation. The results would provide insights for elucidating the molecular mechanism of ABA signaling and developing high dehydration tolerance maize suitable for mechanical harvesting.

**Supplementary Information:**

The online version contains supplementary material available at 10.1186/s12870-022-03751-1.

## Background

Maize (*Zea mays*) is one of the three major food crops globally and is also an important resource for biofuel production [1]. In the last decades, maize production in developed countries (e.g. USA) has gone through a process of rapidly mechanized transformation from planting to harvesting [2]. However, mechanical kernel harvesting of maize has not been fully utilized in many maize production areas in China, partly due to the absence of suitable cultivars capable of rapid desiccation during seed maturation. The greatly reduced moisture content in mature maize kernels is critical for mechanical harvesting [3]. The initiation of rapid desiccation during seed maturation is regulated by abscisic acid (ABA) signaling [4]. Moreover, ABA regulates a variety of gene expression and protein accumulation, mainly late embryogenesis abundant (LEA) proteins and heat shock proteins (HSPs) [5–7], promoting the acquisition of desiccation tolerance of seeds [8, 9].

Maize *viviparous-5* (*vp5*) mutant is deficient in ABA biosynthesis [10], with much reduced ABA content in seeds, roots and leaves compared to normal *Vp5* [11]. The content of ABA in mature embryos and endosperms of the *vp5* seeds were about 10 and 42% of those of the *Vp5* counterparts [12]. ABA plays a vital role in many physiological processes in plants, from germination to senescence [13, 14]. The regulatory network of ABA influencing maize seed maturation were largely unknown. Thus, the combination of *vp5* and *Vp5* is used for analyzing ABA-related cellular events in maize in our [7, 15–17] and others’ studies [18–22]. Specifically, we have identified the differential protein changes between mature *vp5* and *Vp5* embryos by proteomic approaches and a set of ABA-dependent differential abundance proteins [7].

RNA sequencing (RNA-Seq) is a powerful transcriptomic tool using deep-sequencing technologies [23]. The transcriptomic data generated by RNA-Seq not only quantify the level of gene expression, but facilitate the identification of novel transcripts or genes. Maize seed maturation is a complex and dynamic process involving a large number of genes and complex regulatory networks [24]. RNA-Seq has been widely used in exploring maize seed development [24–26], germination [27] and stress responses [27, 28]. At present, we know little about genome-wide changes in gene expressions affected by ABA-synthesis-deficiency in the mutant *vp5* compared to normal *Vp5*. Therefore, it is necessary to compare transcriptome changes between developing *vp5* and *Vp5* embryos.

In the present work, developing *vp5* and *Vp5* embryos were compared regarding with ABA content and transcriptome changes. By RNA-Seq and weighted correlation network analysis (WGCNA), we aimed to identify ABA-related changes in metabolic pathways and differentially expressed genes (DEGs) between *vp5* and *Vp5* during seed maturation.

## Results

### Dynamic changes of ABA content in developing embryos

**Fig. 1:**
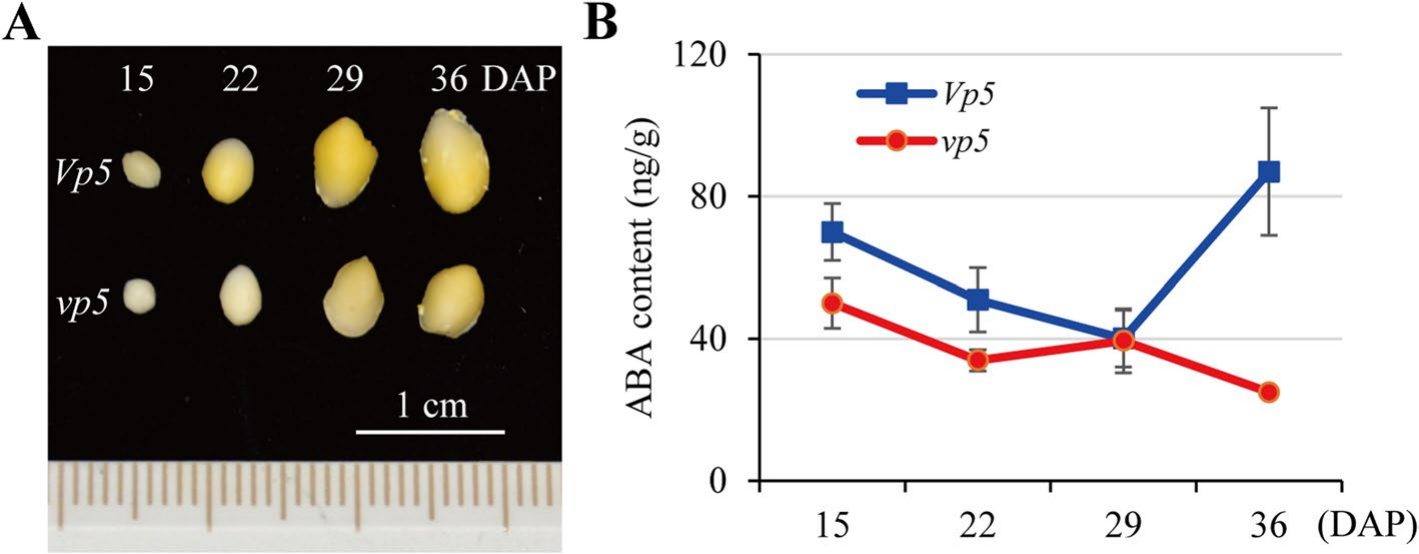
The change in ABA content during *Vp5* and *vp5* seed maturation. **A**, young embryos sampled at 15, 22, 29 and 36 DAP, respectively. **B**, comparison of ABA content between *Vp5* and *vp5* embryos

In order to correlate the changes of ABA content to the process of seed maturation, ABA in developing embryos of *vp5* and *Vp5* was quantitatively analyzed using ultrahigh-performance liquid chromatography coupled to electrospray ionization tandem spectrometry (UPLC/ESI-MS/MS). Before 15 DAP, embryos were too small to be separated from endospermic tissues. Embryos were sampled for ABA assays at 15, 22, 29, 36 DAP, respectively (Fig. 1). The *vp5* embryos were a little smaller than the *Vp5* embryos of the same date. From 15 to 29 DAP, ABA content decreased in both genotypes, with higher levels in *Vp5* embryos. From 29 DAP onward, ABA content increased rapidly in *Vp5* embryos but decreased gradually in *vp5* embryos. At 36 DAP, *vp5* embryos contained only 1/4 ABA of *Vp5* embryos, showing a significant difference. A possible explanation for the above changes was that during early stage of seed development, ABA in young embryos was mainly transported from mother plants, whereas at later stage it was synthesized within embryos. That is, the mutation in ABA-synthesis-deficiency in *vp5* became more prominent at the late stage of seed maturation. Therefore, 36 DAP would be a critical date for analyzing the effect of differential ABA content on gene expression in developing *vp5* and *Vp5* embryos.

### Quality analysis of RNA-Seq data

In order to analyze the effect of ABA content on gene expression during embryo development, the young embryos of *vp5* and *Vp5* were sampled at 15, 22, 29 and 36 DAP, respectively, and subjected to RNA-Seq in three biological replicates. As a result, a total of 167.44 G clean data was obtained, with each sample consisting of 6.92–7.01 G. Quality check showed that 94.32–96.45% had a quality score of 30 or higher for single and paired end reads, an average GC content of 54.42% (Table S1). Of all the reads, 88.85–92.59% were aligned to the reference sequences (Table S2).

The reads with a unique alignment position on the reference sequences were much higher than those with multiple alignment positions, regardless of positive strands or negative strands. Therefore, RNA-Seq data in the present work were of high-quality and accuracy, suitable for gene expression analysis and DEGs identification. The number of high-quality transcripts generated from RNA-Seq was similar for both genotypes. It became smaller from 15 to 36 DAP, being 20,216, 19,809, 19,541 and 18,809 in *Vp5* and 20,120, 19,634, 19,408 and 19,030 in *vp5*.

**Fig. 2:**
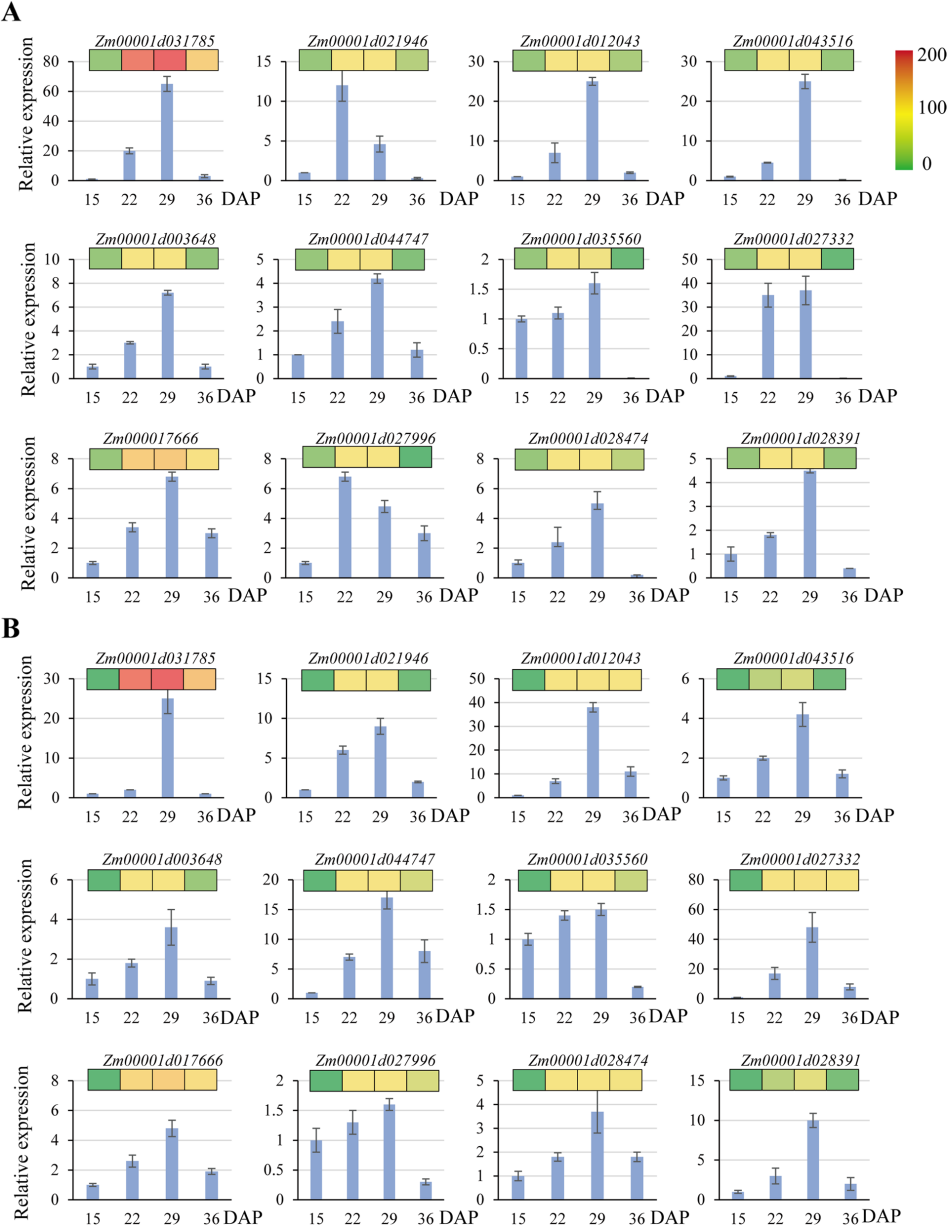
Validation of expression levels of 12 randomly selected DEGs by RT-qPCR. Relative gene expression levels were represented in column. The inserted heatmap was drawn based on RNA-Seq results. Error bar represents standard deviation (*n* = 3). **A**, *Vp5*; **B**, *vp5*

In addition, the expression of 12 randomly selected genes during seed maturation were analyzed by reverse transcription quantitative PCR (RT-qPCR). The expression patterns of these genes were the similar as those of RNA-Seq results (Fig. 2), indicating high-quality of RNA-seq data.

### Gene expression analysis and DEGs identification

**Fig. 3:**
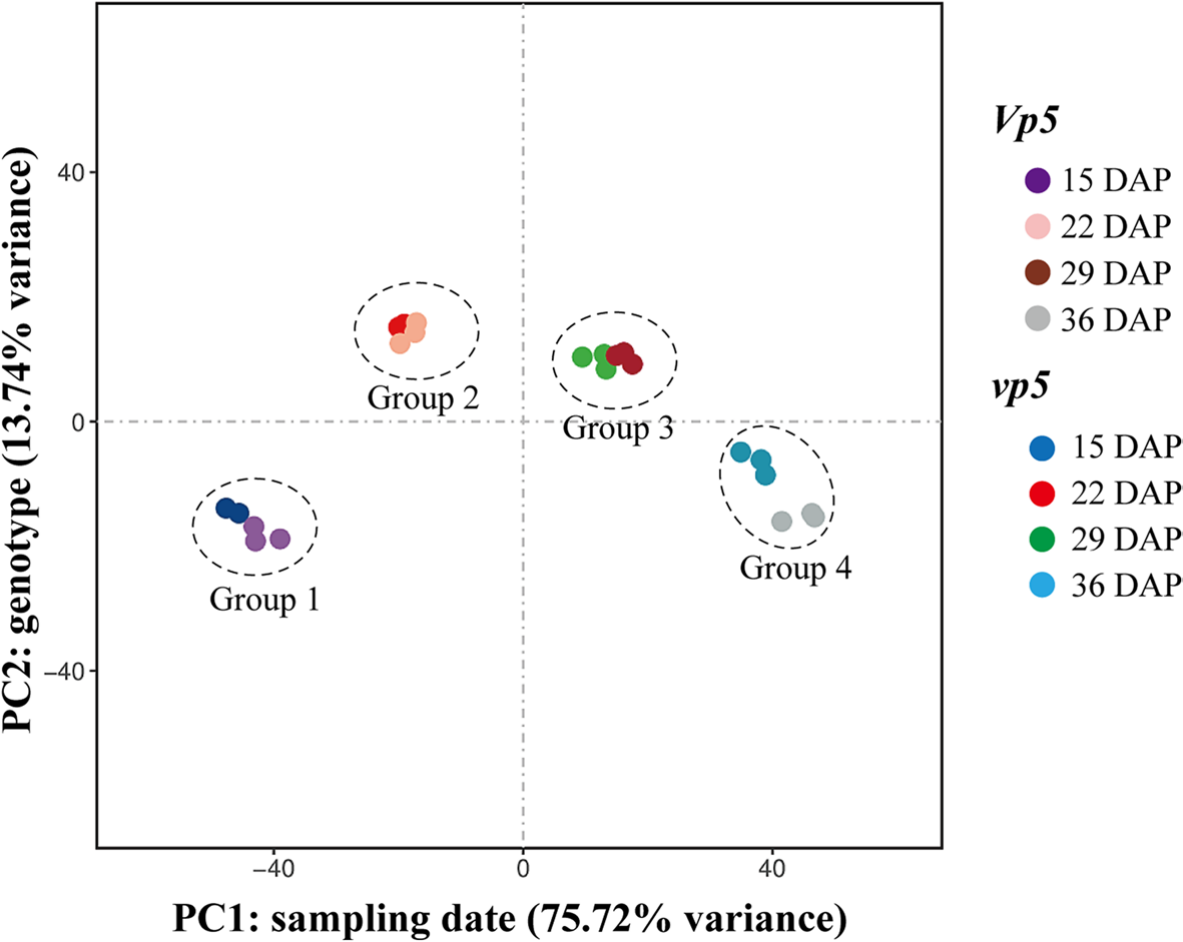
Principal component analysis (PCA) of RNA-seq data

Correlation comparison of gene expression among the 12 *Vp5* samples (collected at four dates in 3 biological replicates) were of high correlation, with a r^2^ value of 0.98 or higher; it was similar for the 12 *vp5* samples (r^2^ > 0.97) (Fig. S1). Principal components analysis (PCA) showed that total 24 samples of both genotypes were clearly divided into four groups according to sampling date (Fig. 3). That is, the main distinctive pattern in the data was associated with the sampling date rather than the genotype, suggesting that the developing stage was the critical factor affecting gene expression. Especially, the principal components of the four groups were widely separated and the divergence were highly significant at 36 DAP. Moreover, the two genotypes within group 4 (at 36 DAP) were well separated. This is consistent with the change in ABA content among the four sampling dates.

**Fig. 4:**
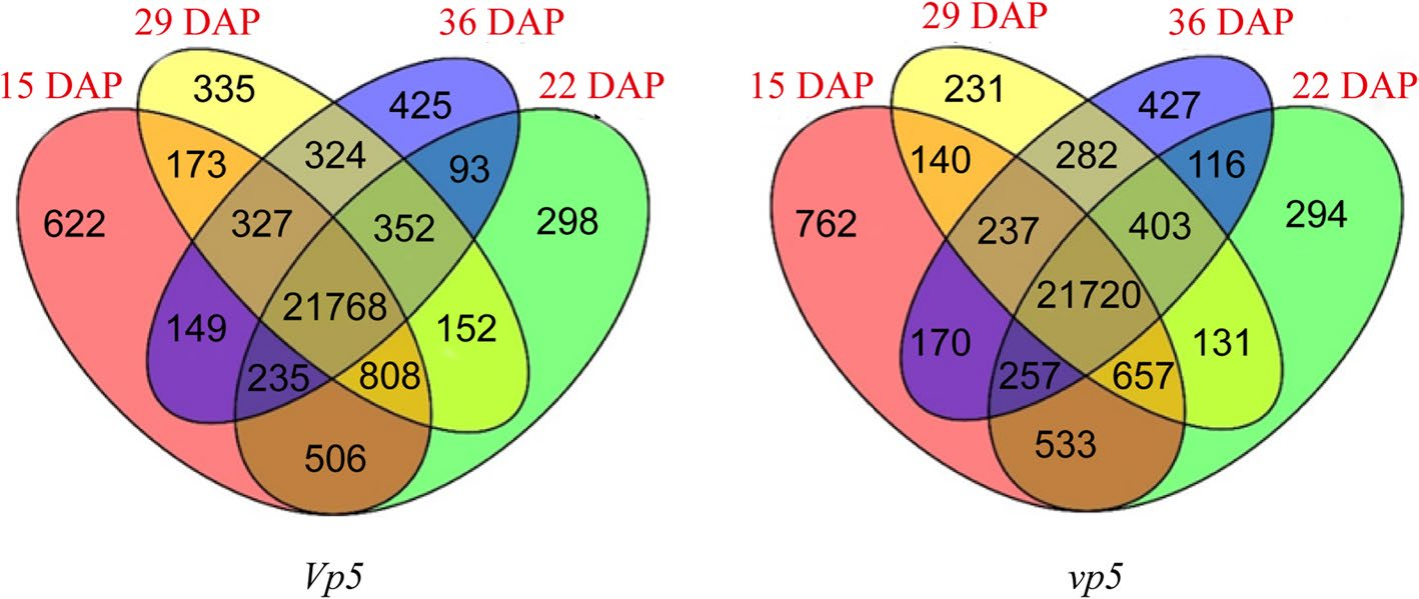
Summary of the genes detected in embryos during seed maturation in Venn diagram

The changes in gene expressions in *Vp5* and *vp5* embryos during seed maturation were compared respectively (Fig. 4). In *Vp5* embryos, 21,768 genes were expressed in all examined dates, with 622, 335, 425 and 298 DEGs identified in 15, 22, 29 and 36 DAP, respectively. In *vp5* embryos, 21,720 genes were expressed in all examined dates, with 762, 231, 427 and 294 DEGs identified in 15, 22, 29 and 36 DAP, respectively. Obviously, an overwhelming number of the genes was always expressed during seed maturation in both genotypes.

**Fig. 5:**
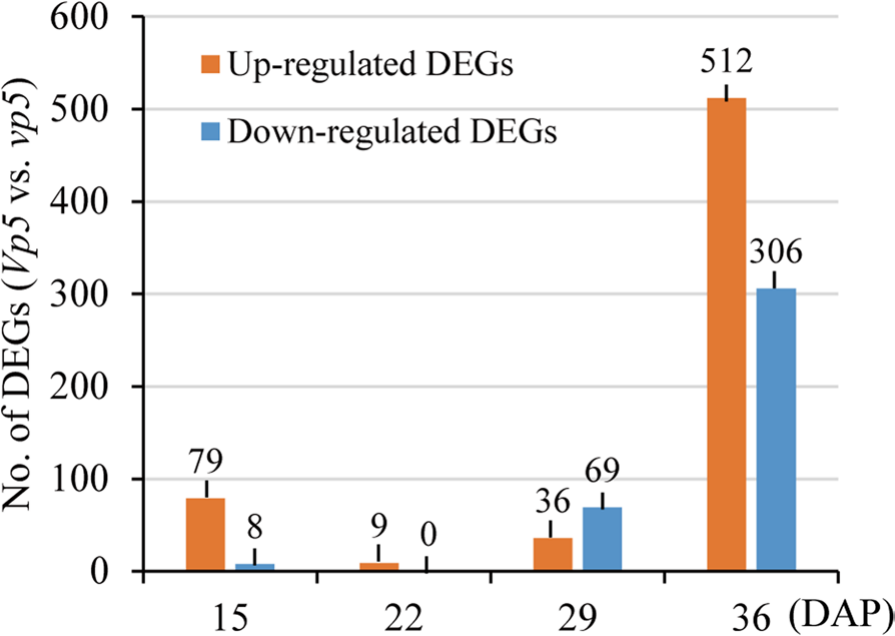
The number of DEGs identified between *Vp5* and *vp5* embryos. The changes in gene expression levels were calculated by *Vp5* versus *vp5*

We were more interested in differential gene expressions of the same date between both genotypes. Thus, the changes in gene expressions between *Vp5* and *vp5* embryos of the same period were compared in pairs. As a result, a total of 1019 DEGs were identified between *Vp5* and *vp5* (*P* < 0.05, |fold-change| ≥ 2), including 636 up-regulated DEGs in *Vp5* embryos and 383 up-regulated DEGs in *vp5* embryos (Fig. 5, Table S3).

In general, the number of DEGs increased with seed maturation. At 15 DAP, 79 DEGs were up-regulated in *Vp5* embryos, many of which encode the proteins involved in seed maturation and abiotic/biotic responses, such as embryonic proteins (*Zm00001d008241*, *Zm00001d029062*), seed maturation protein (*Zm00001d035000*, *Zm00001d044022*), ABA-responsive protein (*Zm00001d023664*, *Zm00001d025401*, *Zm00001d040787*, *Zm00001d042779*), dehydrin (*Zm00001d017547*), etc. In contrast, the genes related to DNA replication (*Zm00001d009374*, *Zm00001d030614*) were highly expressed in *vp5*. This suggested that the processes related to seed maturation and stress response were initiated earlier in *Vp5* than in *vp5*.

At 22 DAP, only 9 DEGs were detected and all were up-regulated in *Vp5*. It should be noted that the gene encoding zinc finger protein (*Zm00001d010800*, *Zm00001d039495*) was up-regulated in *Vp5*, whereas the corresponding genes were up-regulated at 15 DAP in *vp5*.

At 29 DAP, a total of 105 DEGs were identified between *Vp5* and *vp5*. One of significant changes in gene expression was that many genes encoding storage proteins and stress proteins were highly up-regulated in *Vp5*, including zein (*Zm00001d005793*, *Zm00001d019155*, *Zm00001d020591*, *Zm00001d048809*, *Zm00001d048817*, *Zm00001d048848*, *Zm00001d048850*), dehydration- (*Zm00001d031861*), wound-responsive protein (*Zm00001d002160*), and HSPs (*Zm00001d003554*), whereas the genes encoding storage proteins were lowly expressed in *vp5* embryos during all sampling dates, which obviously is unfavorable to normal seed maturation.

At 36 DAP, the majority of the DEGs (818, 80.28%) were detected, with 521 up-regulated DEGs in in *Vp5* embryos and 306 up-regulated DEGs in *vp5* embryos. Significantly, many specific genes involved stress (e.g. dehydration) response, seed maturation, seed germination and growth were highly up-regulated at 36 DAP by several even 10 times higher in *Vp5* than in *vp5,* including genes encoding auxin responsive proteins (*Zm00001d000288*, *Zm00001d002304*, *Zm00001d005804*, *Zm00001d006753*, *Zm00001d018414*, *Zm00001d030993*), dehydration-responsive proteins (*Zm00001d017592*, *Zm00001d022416*), growth-regulating factor (*Zm00001d000238*), cell division control protein (*Zm00001d031222*, *Zm00001d042810*), WRKY transcription factor (*Zm00001d004086*, *Zm00001d012482*, *Zm00001d020137*, *Zm00001d020492*, *Zm00001d032265*, *Zm00001d043025*, *Zm00001d043950*, *Zm00001d053746*), nitrate transport (*Zm00001d003287*), potassium high-affinity transporter (*Zm00001d003861*), UDP-glycosyltransferase (*Zm00001d004248*, *Zm00001d011649*, *Zm00001d032866*, *Zm00001d039607*, *Zm00001d039642*, *Zm00001d053715*), stress protein (*Zm00001d006016*), LEA (*Zm00001d009382*), and HSPs (*Zm00001d015777*, *Zm00001d025508*, *Zm00001d028561*, *Zm00001d039936*, *Zm00001d047841*). This indicated that the machinery of gene expression and protein synthesis in *Vp5* embryos have been efficiently run as programmed for successful seed germination and possible environmental stress. However, the genes encoding storage proteins were highly up-regulated in *vp5*, including zein (*Zm00001d035760*, *Zm00001d037436*, *Zm00001d048850*), globulin 3 (*Zm00001d038597*).

Combined with the results in Figs. 1 and 3, around 36 DAP was a key period for analyzing the differential changes in ABA-regulated gene expressions and cellular events between *Vp5* and *vp5* embryos*.*

### GO and KEGG enrichment analysis of DEGs at 36 DAP

We conducted Gene Ontology (GO) and Kyoto Encyclopedia of Genes and Genomes (KEGG) enrichment analyses to describe the function of DEGs and potential cellular pathways or signaling associated with ABA differential accumulation during maize seed maturation.

**Fig. 6:**
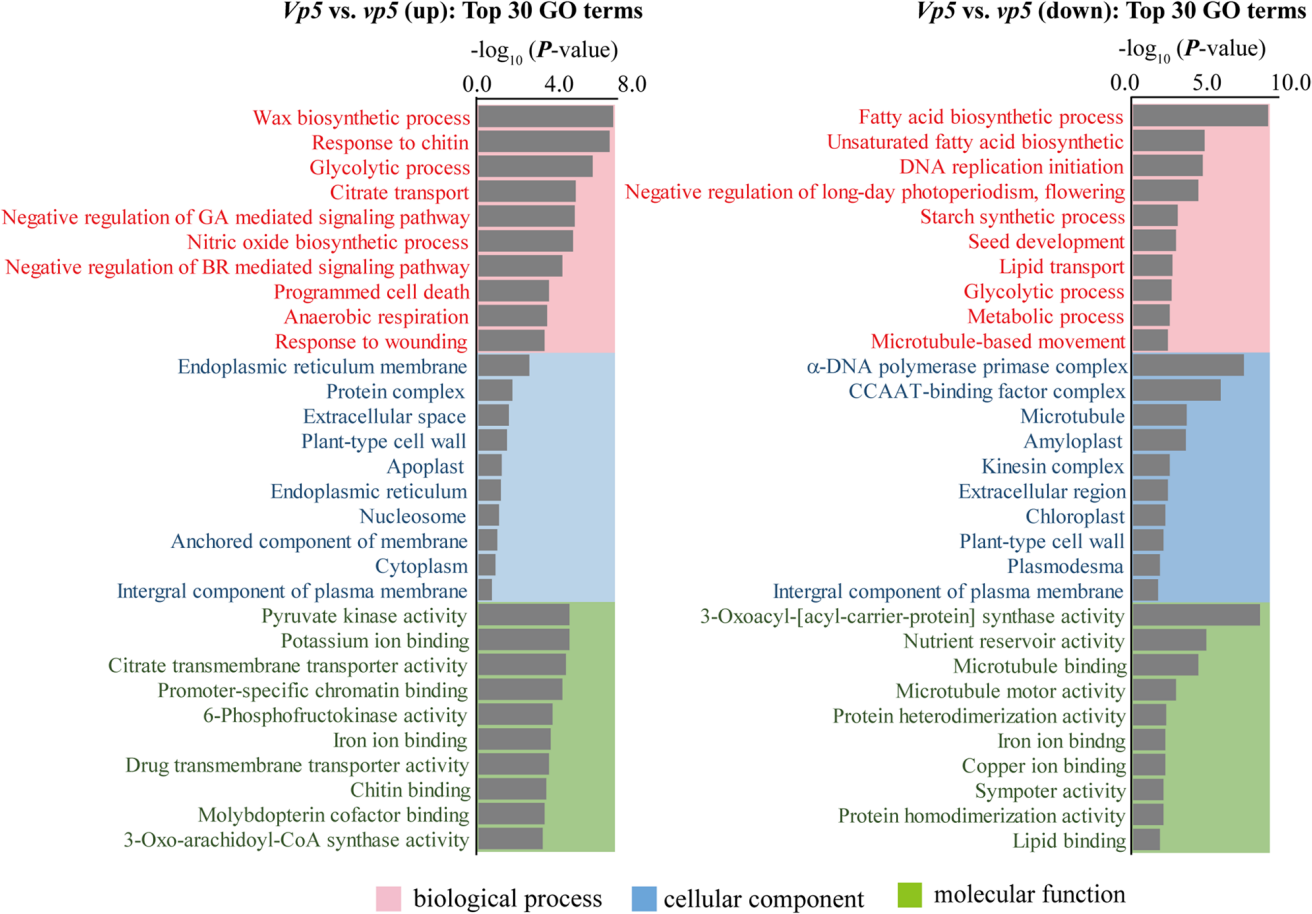
GO enrichment analysis of DEGs in *Vp5* versus *vp5* at 36 DAP

According to GO terms, the DEGs identified at 36 DAP were divided into three categories: biological process, cellular component and molecular function (Fig. 6). In *Vp5*, several enrichment processes were all related to ABA signaling, such as response to wounding, response to chitin, and programmed cell death, etc. Molecular functions enriched included pyruvate kinase activity, potassium ion binding, citrate transmembrane transporter activity. Cellular components were enriched in endoplasmic reticulum membrane, protein complex and extracellular space.

In *vp5*, biological processes were enriched in metabolic pathways, including starch synthesis, lipid synthesis, DNA synthesis, and other metabolic events were still active in *vp5* during the late stage of seed maturation. The enriched molecular functions included 3-oxoacyl-[acyl-carrier-protein] synthase activity, nutrient reservoir activity, and microtubule binding, etc. Cellular component was enriched in DNA polymerase primase complex, CCAAT-binding factor complex and microtubule among others.

**Fig. 7:**
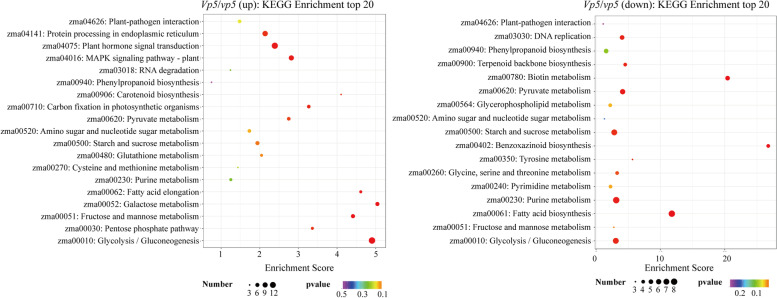
KEGG pathway analysis of DEGs in *Vp5* versus *vp5* at 36 DAP

The potential pathways generated from KEGG enrichment analysis were represented in Fig. 7. The significantly enriched pathways in *Vp5* included plant hormone signal transduction, glycolysis/gluconeogenesis, protein processing in endoplasmic reticulum and mitogen-activated protein kinase (MAPK) signal pathway. Whereas the pathways of purine metabolism, fatty acid biosynthesis and starch and sucrose metabolism were significantly enriched in *vp5*.

### Identification of DEGs related to differential ABA synthesis by WGCNA

**Fig. 8:**
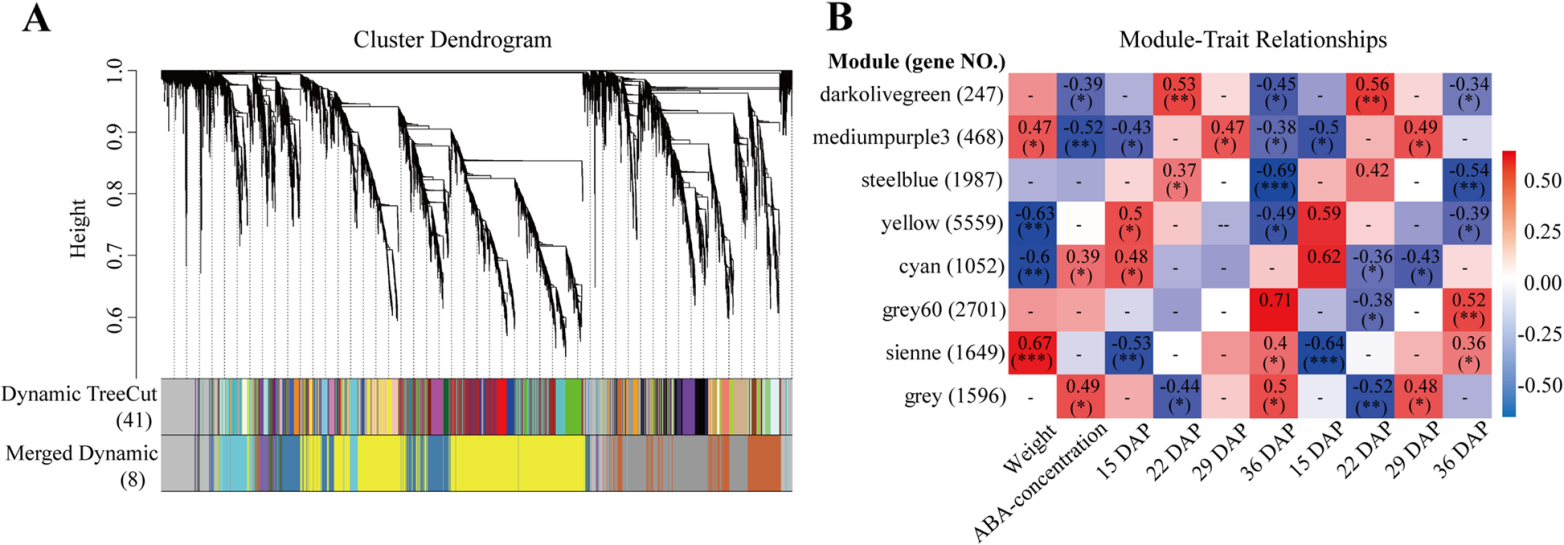
Network analysis of dendrogram showing modules identified using WGCNA. **A**, Hierarchical cluster tree showing co-expressing modules. **B**, Heatmap of module-ABA weight correlations and corresponding *P*-values

In order to identify highly correlated DEGs that co-occurred and responded to differential ABA synthesis during seed maturation, we used WGCNA to analyze gene expressions in all 24 samples of both genotypes collected at 15, 22, 29 and 36 DAP, respectively. After removal of the genes with low fluctuation in expression (standard deviation ≤0.3), 15,259 of 24,233 genes were subjected to pairwise correlation analysis regarding gene expression and sorted into different modules, i.e., highly correlated gene clusters. The genes in the same modules shared high correlation coefficients, constructing eight co-expression networks (Fig. 8).

**Fig. 9:**
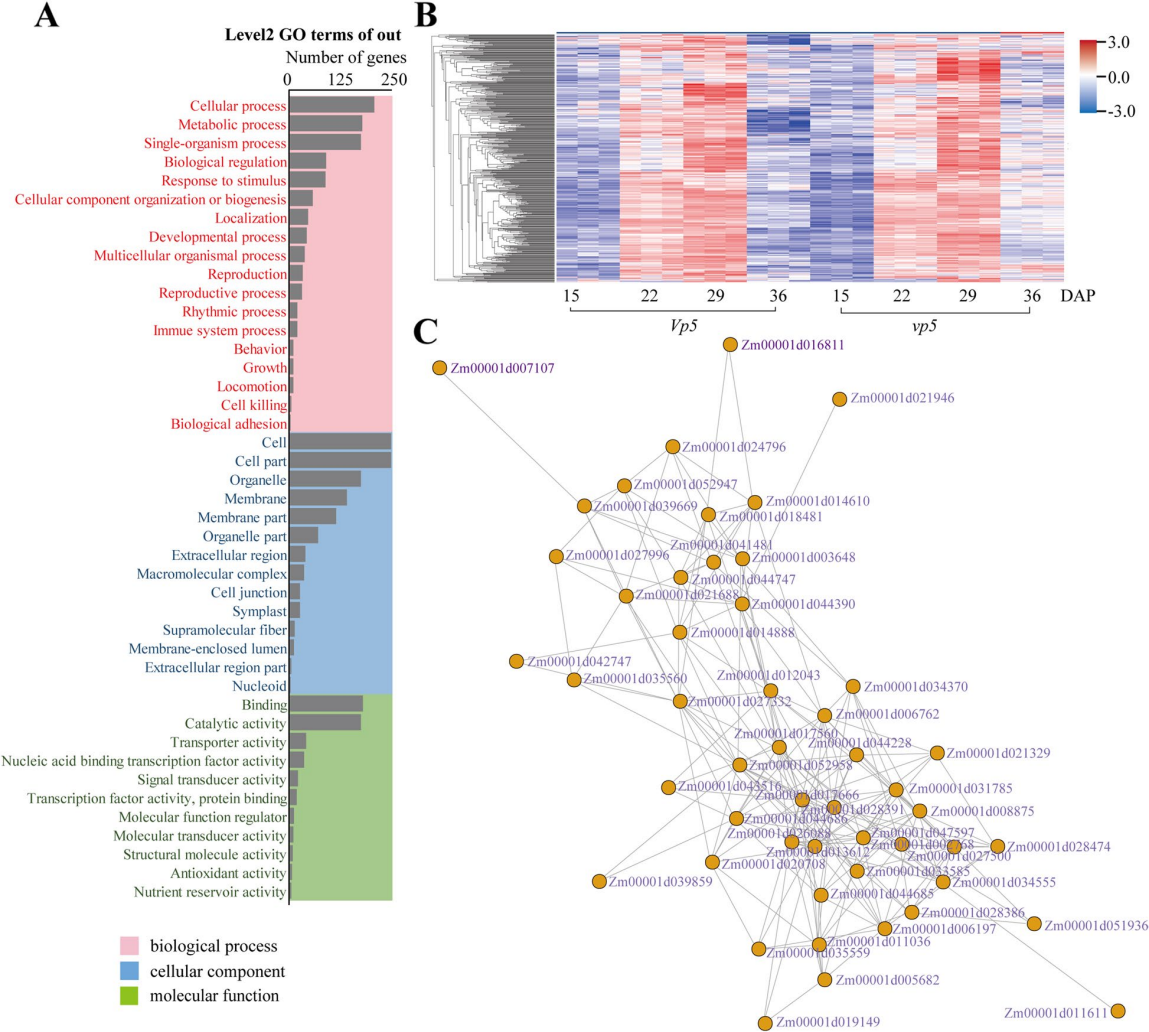
Characterization of genes in midumpurple3 module. **A**, GO enrichment analysis. **B**, Heatmap of gene expression profile. **C**, Co-expression network analysis

Next, these modules correlated with differential ABA synthesis were identified. The midumpurple3 module was highly correlated with ABA content (*P* < 0.01, r^2^ = 0.52, Fig. 8). Only a small number of in the midumpurple3 module were the DEGs (Table S4) based on their expression changes. This module includes 18 transcription factors, such as MYB family, EREBP, ZIM family and bHLH28, which may be involved in regulating ABA-related gene expression during seed maturation. GO enrichment analysis was performed to describe the functions of the genes in the module (Fig. 9), mainly involved in cellular process, metabolic process and single organic process in biological process, with binding function and catalytic activity. As revealed above (Fig. 6), these analyses also suggested that *vp5* embryos had strong cell metabolic activities during the late stage of seed maturation.

Finally, the hub genes in ABA-related modules were screened based on the heatmap of gene expression and ABA content (Fig. 8). The DEGs in the module midumpurple3 were highly up-regulated in 29 DAP and negatively correlated with ABA content. The top 50 genes with the highest connectivity in the module were analyzed (Fig. 9). The genes with the highest connectivity included those encoding thiamine biosynthesis (*zm00001d044228*), protein NRT1/PTR family 7.2 (*zm00001d017666*), receptor like protein kinase 4 (*zm00001d031785*), protein kinase superfamily protein (*zm00001d008875*), nonspecific lipid transfer protein (*zm00001d027332*), tubulin β-4 (*zm00001d013612*) and calmodulin 7 (*zm00001d047597*). Of them, thiamine biosynthesis gene and protein NRT1/PTR family 7.2 gene belong to NPF family genes, which are identified as nitrate or tripeptide transporters and can transport phytohormones ABA, indole-3-acetic acid (IAA) and gibberellin (GA), and affect the accumulation of nitrogen during plant embryo development. It is speculated that these genes may be the key genes of the midumpurple3 module and involved in ABA regulation of maize seed maturation.

### RT-qPCR validation of the expression of key genes regulated by ABA

**Fig. 10:**
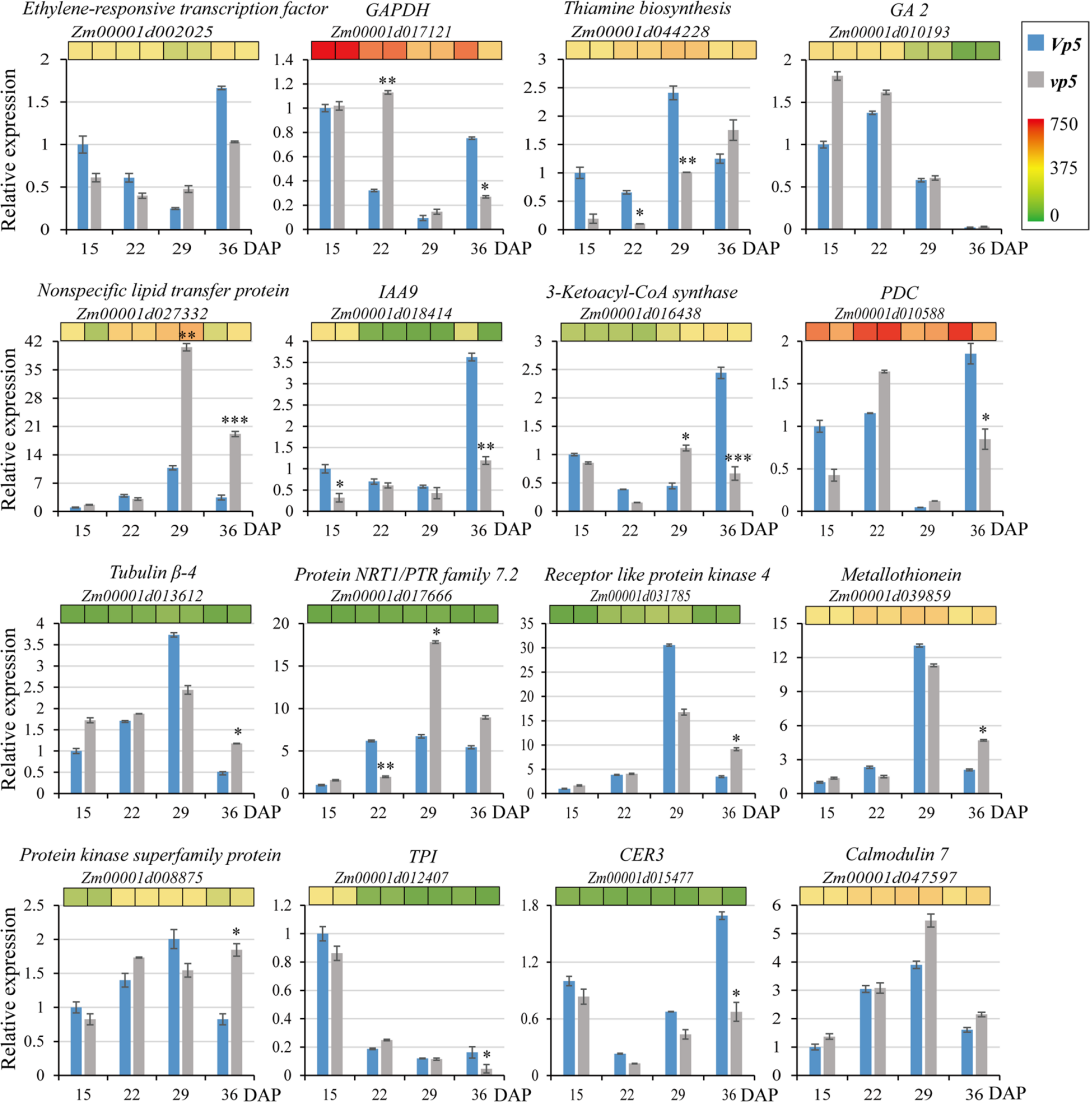
RT-qPCR verification of the expression of key genes regulated by ABA. Relative gene expression levels were represented in column. The inserted heatmap was drawn based on RNA-Seq results. Error bars represent standard deviation (*n* = 3). Asterisks indicate levels of significance of differential expression (Student’s t-test: * *P* < 0.05, ** *P* < 0.01, *** *P* < 0.01)

Based on the KEGG and WGCNA analysis, a set of DEGs negatively correlated with ABA content were identified (Table S4, Figs. 7 and 8). A total of 16 DEGs were selected to verify their expression levels during embryo development in the context of differential ABA content between *Vp5* and *vp5*. RT-qPCR analysis showed that the expression trends of these DEGs were similar with the results of RNA-Seq (Fig. 10). In particular at 36 DAP embryos, *glyceraldehyde-3-phosphate dehydrogenase* (*GAPDH*), *IAA9*, *3-ketoacyl-CoA synthase*, *pyruvate decarboxylase* (*PDC*), *triose phosphate isomerase* (*TPI*), *ECERIFERUM 3* (*CER3*) were highly up-regulated in *Vp5*, whereas *nonspecific lipid transfer protein*, *tubulin β-4*, *receptor like protein kinase 4*, *metallothionein* and *protein kinase superfamily protein* were highly up-regulated in *vp5*.

## Discussion

ABA plays various roles in plant life cycle, such as seed maturation, dormancy, and germination, and stress responses [13, 14]. Maize mutant *vp5* is deficient in ABA synthesis in seeds [10], due to the first step of ABA synthesis (catalyzed by phytoene desaturase) is blocked in *vp5* seeds [29], mainly in embryos [12]. To identify the DEGs associated with differential ABA synthesis, we here compared the developing embryos of *Vp5* and *vp5* by RNA-Seq analysis*.*

### DEGs and differential pathways between *Vp5* and *vp5* embryos

The ABA content in developing embryos differed remarkedly between *Vp5* and *vp5*, especially at 36 DAP with 3 times more in *Vp5* than in *vp5* (Fig. 1B), as found in previous study [30]. RNA-Seq have detected 23,612 (36 DAP, *vp5* embryos) to 24,588 (15 DAP, *Vp5* embryos) expressed genes, which were comparable to 22,790 expressed genes detected in young maize seeds [24]. Overall, most DEGs (818) between *Vp5* and *vp5* were detected at 36 DAP, i.e., ABA-regulated DEGs were mainly expressed at the late stage of seed maturation. This could be explained that even *vp5* embryos contained a substantial ABA (ca. 7/10 of *Vp5* ABA content), mainly transporting from maternal organs, but it lacks the ability of responding to ABA [31].

GO and KEGG enrichment analyses revealed significantly differential changes in DEGs and pathways between both genotypes, especially at 36 DAP. The DEGs up-regulated in *Vp5* were mainly involved in wax biosynthesis, glycolysis, negative regulation of GA and brassinosteroids (BR) signaling and stress responses to (wounding, chitin), which are all important to seed maturation and subsequent seed germination. These pathways enriched in 36 DAP *Vp5* embryos represent normal events occurring during seed maturation, whereas the pathways enriched in 36 DAP *vp5* embryos were still characterized by active metabolic processes (e.g. the syntheses of starch, lipid and DNA).

Wax biosynthesis helps plants reduce water loss and resist environmental stresses such as dehydration [32]. CER3 [33, 34], 3-ketoacyl-CoA synthase [35], O-acyltransferase WSD1 [36] are all key enzymes involved in wax biosynthesis. In ABA-insensitive *Arabidopsis* mutants, the expression of wax biosynthesis genes was lowered under dehydration or ABA treatment [32]. Similarly, the expressions of wax biosynthesis genes encoding 3-ketoacyl-CoA synthase (*Zm00001d016438*) and CER3 protein (*Zm00001d015477*, *Zm00001d046865*) in *Vp5* were significantly higher than those in *vp5* (Fig. 10). Therefore, ABA signaling promotes the expression of genes involved in wax biosynthesis and facilitates the acquisition of seed dehydration tolerance.

TPI, GAPDH and PDC are enzymes involved in the catalytic glycolysis step [36, 37]. In this work, the expression of genes encoding TPI (*Zm00001d012407*), GAPDH (*Zm00001d017121*, *Zm00001d051001*) and PDC (*Zm00001d010588*) was higher in *Vp5* at 36 DAP, which indicated that the energy metabolism process of *vp5* was weak during seed development (Fig. 10). Moreover, pyruvate kinase is involved in glucose metabolism and fatty acid metabolism and its expression is significantly regulated by ABA content in plants [38]. Here we found that seven genes encoding pyruvate kinase were down-regulated in *vp5*, consistent with the previous results [38].

Seed dormancy in cereal crops is necessary for preventing premature seed germination on mother ears (i.e., vivipary). Phytohormones often mutually regulate signaling and physiological processes, such as ABA, ethylene and GA in seed maturation [39]. ABA-synthesis deficient mutants, such as maize *vp* mutants and *Arabidopsis ABA-deficient* (*aba*) and *ABA-insensitive* (*abi*) mutants [40] produce premature germinating seeds. Altering the expression of the ABA-synthetic and catabolic genes may affect the germinability of barley grains under field conditions [41]. Moreover, the application of exogenous auxin (IAA) enhances the inhibitory effect of ABA on seed germination in wheat [42] and Arabidopsis [43]. We here found that the expressions of IAA genes, e.g. *Zm00001d018414*, *Zm00001d030993*, and *Zm00001d000288* in 36 DAP *Vp5* embryos were highly up-regulated (2–12 times higher than those in *vp5*), suggesting a possible role of ABA in regulating IAA synthesis. The expression of ethylene-responsive transcription factor (*Zm00001d002025*, *Zm00001d010175*, *Zm00001d026563*, *Zm00001d043205*) in *Vp5* is more than twice that in *vp5*. Moreover, the expressions of two GA-regulatory proteins GA2 (*Zm00001d010193*) and GA10 (*Zm00001d013222*) were twice more in *vp5* than in *Vp5* at 36 DAP (Fig. 10), suggesting that ABA and GA may antagonize each other to regulate seed dormancy [44, 45].

In addition, the genes encoding LEA protein (*Zm00001d009382*) and 17.4 kDa HSPs (*Zm00001d028561*, *Zm00001d039936*, *Zm00001d047841*) in *Vp5* at 36 DAP were highly up-regulated*,* consistent with our proteomic results [7]. LEA protein and HSPs functions as a dehydration protective agent during mature dehydration and stress responses [7, 46, 47]. These results show that ABA can promote the acquisition of dehydration tolerance and affects seed maturation by regulating the accumulation of LEA protein and HSPs.

### Potential hub genes related to differential ABA synthesis

Weighted correlation network analysis (WGCNA) has been widely used in data analysis of RNA-seq recently, e.g. plants, animals, microorganisms. It can find clusters (modules) of highly correlated genes to external sample traits by eigengene network methodology [48, 49].

In the present study, we identified a module negatively correlated with ABA content through WGCNA analysis. The genes with the highest connectivity in the module was thiamine (vitamin B1) biosynthesis gene (*THI2*) (*Zm00001d044228*). Maize *THI2* is homologous to *Arabidopsis THI1*, which is highly expressed in differentiated tissues and regulates organ formation [50]. Thiamine is necessary for seed development, and tissues and organs with high cell division activity need thiamine [51]. The gene with the second highest connectivity in the module is the gene encoding protein NRT1/PTR family protein (*Zm00001d017666*). NPF was identified as a peptide transporter and can transport a variety of substrates, including dipeptides, nitrates, nitrites, chlorides, glucosinolates and amino acids, as well as IAA, ABA, jasmonate and GA [52]. The gene (*Zm00001d047597*) encoding calmodulin 7, which plays an important role in calcium signal transduction, including ABA mediated seed germination. During seed germination, the expression of calmodulin is inhibited by ABA [53].

The gene encoding metallothionein (*Zm00001d039859*) may be regulate by ABA. During seed germination, storage proteins were decomposed to provide raw materials and energy for seedling growth. Metallothionein can reduce the disulfide bond of storage protein, increase its solubility and contribute to the mobilization of storage protein [54]. In this study, a variety of genes encoding aspartate or cysteine proteolytic enzymes were identified as potential hub genes related to differential ABA synthesis. The expressions of these genes in *vp5* were higher than that in *Vp5* at 36 DAP. It is speculated that ABA may alleviate seed dormancy and promote seed germination via negatively regulating these genes, which is consistent with the low degree of dormancy of *vp5* seeds.

## Conclusions

We here used two maize materials (*Vp5*, normal; *vp5*, ABA-deficient mutant) to identify the key genes and pathways associated with differential ABA synthesis during seed maturation. Bioinformatics and WGCNA revealed the potential pathways involving these DEGs and the highly correlated gene clusters (modules) associated with differential ABA synthesis. Several hub genes possibly involved in ABA regulation of maize seed maturation were screened in view of correlation between gene expression and ABA content. These results obtained here would provide insights for elucidating the molecular mechanism of ABA signaling and developing high dehydration tolerance maize suitable for mechanical harvesting.

## Materials and methods

### Maize field planting and sampling

Maize seeds of ABA-deficient mutant *vp5* and its wild-type *Vp5* were used in the present study. The heterozygous seeds (*Vp5/vp5*, kindly provided by Maize Genetics Stock Center, Urbana, IL, USA) were grown at the university farm (Zhengzhou, China) under field conditions. In selfing-ears, homozygous kernels (*vp5/vp5*) appeared white due to lack of carotenoids, obviously different from yellow wild-type kernels [11]. The *vp5* and *Vp5* kernels were collected on the same ears at 15, 22, 29 and 36 DAP, respectively (Fig. S2). The embryos were manually separated with scalpel and forceps, frozen in liquid N_2_ and stored in a freezer (− 80 °C) before ABA and RNA extraction.

### Extraction of ABA in developing embryos

ABA was extracted as described previously [55] with some modifications. Briefly, 30 mg frozen embryo material was ground were with mixer mill MM400 (Retsch GmbH, Haan, Germany) in a 2 mL Eppendorf tube at 20 Hz for 2 min and then extracted in 500 μL extraction solvent (isopropanol: water: FA, 2:1:0.002) for 20 min (− 20 °C), followed by ultra-sonication (0–4 °C) for 30 min. The labeled d6-ABA was added as internal standards. Afterwards, 1 mL of chloroform was added to the tube and kept at − 20 °C for 20 min, followed by ultrasonic treatment at ice bath for 5 min. The extract was centrifuged at 12,000 *g* for 5 min. The resultant bottom layer (ca. 900 μL) was pipetted to a brown LC/MS (Liquid Chromatography/Mass Spectrometry) injection bottle for evaporation drying. The pellets were resolubilized in 200 μL of 80% methanol, filtered through a 0.22 μm PTFE filter (Waters, Milford, MA, USA). ABA were determined by LC/MS/MS in six independent replicates for each sample. Quantification was done by the creation of calibration curves including each of the unlabeled ABA. Ten standard solutions were prepared ranging from 0.05 to 200 ng/mL and for each solution a constant amount of internal ABA standard was added.

### ABA assay by UPLC/ESI-MS/MS

The quantitative assay of ABA in developing maize embryos was preformed using ultrahigh-performance liquid chromatography coupled to electrospray ionization tandem spectrometry (UPLC/ESI-MS/MS) [55]. The HPLC system consisted of Aquity UPLC™ System (Waters, Milford, MA, USA) quaternary pump equipped with an autosampler. A Poroshell 120 column (EC-C18, Agilent, 3 × 100 mm, 2.7 μm) was used for ABA separation, with 3 μL injection volume. Gradient elution was done with 0.1% formic acid/H_2_O (solvent A) and acetonitrile with 0.1% formic acid/acetonitrile (solvent B) at a constant flow rate of 0.6 mL/min. Mass spectrometry (MS) and MS/MS were done in multiple reaction mode on an API 5500 triple quadrupole mass spectrometer (AB Sciex, Boston, MA, USA), assisted with electrospray ionization (ESI) and Analyst® Software 1.6.2. The optimized conditions were as follows: curtain gas: 30; collision gas: 8; ion spray voltage: 3000 V; temperature: 500 °C; ion source gas 1: 35; gas 2: 45.

### RNA extraction and RNA-Seq

The frozen maize embryos were ground using liquid N_2_ to make fine powder with mortar and pestle. Total RNA from was with TRIzol™ Plus RNA Purification Kit following the manufacturer’s instructions. DNase-treated RNA samples were run on 1% agarose gel plus 1% formaldehyde to confirm the integrity and quality. RNA was determined an Agilent 2100 Bioanalyzer (Agilent Technologies, Palo Alto, CA, USA). mRNA was fragmented and enriched with by oligo-dT beads magnetic beads. Sequencing library was amplified with the broken mRNA as the templates to synthesize cDNA. The cDNA templates were purified with the kit and enriched by PCR. After quality check, RNA-Seq was performed using HiSeqTM 2500 Sequencing System (Illumina, USA). The raw reads obtained from sequencing were processed with Trimmomatic software to remove low-quality reads.

### DEGs identification

The expression level of each protein-coding gene in each sample was identified by sequence similarity comparison [26]. The reference genomes and the annotation file were downloaded from the ENSEMBL database (http://www.ensembl.org/). The clean data were aligned to the reference genome by HISAT2 [56]. Gene expression was calculated using Cufflinks software based on fragments per kilobase of exon model per million reads mapped (FPKM) value [57] which is the number of fragments from a protein coding gene per kb length per million fragments. The software DESeq2 was used to identify DEGs (*q* ≤ 0.05, expression change fold ≥2).

Function enrichment analysis of Gene Ontology (GO) and Kyoto Encyclopedia of Genes and Genomes (KEGG) were performed in R language against the maize Genome Assembly information [58].

### Statistical analysis

The analysis of variance (ANOVA) was evaluated using a Student’s *t*-test, and differences between samples were considered significant at a probability level of *P* < 0.05. Pearson correlation test was determined in SPSS software (SPSS, Inc., Chicago, IL). In order to create a low-dimensional global map, gene expression data of developing embryos were analyzed using PCA. PCA was performed using the prcomp function in R language. The gene co-expression network was constructed using the weighted gene co-expression network analysis (WGCNA) package in R language [48]. The correlation between genes was calculated by the Pearson correlation matrix and the means of the connecting rod. The results were visualized using R language and python language. Module identification was finished after merging the modules of similar expression profiles. Merge cut height was 0.5. The co-expression network was built and visualized using Cytoscape v 3.8.0 software.

### RT-qPCR

RNA in developing maize embryos was extracted using the Fast Plant Total RNA Kit (Nobelab Biotech Co., Ltd., Beijing, China) and the cDNA was using the kit (Starscript II First-stand cDNA Synthesis Mix With gDNA Remover, GenStar, Beijing, China) according to the manufacturer’s instructions. The gene-specific primers of DEGs were designed using Primer Premier 5.0 software (http://www.premierbiosoft.com/) (Table S5) and commercially synthesized at the Biomed Cooperation (Beijing, China).

RT-qPCR was carried out using StepOnePlus™ Real-Time PCR Instrument Thermal Cycling Block (Applied Bio-systems). The PCR amplification procedure was as follows: an initial denaturation step at 95 °C for 5 min, followed by 40 cycles at 95 °C for 10 s and 60 °C for 30 s. The relative expression level of DEGs was assessed by the 2^-ΔΔCT^ method [59]. Data were presented as relative expression (mean ± SD) from three biological replicates, with *ZmUBI* as reference gene. Significant differences in expression changes between *Vp5* and *vp5* embryos were analyzed by Student’s *t*-test (*P < 0.05, ** *P* < 0.01, *** *P* < 0.001).

## Supplementary Information

**Additional file 1: Table S1.** Statistical analysis of RNA-seq data quality.

**Additional file 2: Table S2.** Comparison of RNA-seq data with reference genome.

**Additional file 3: Table S3.** DEGs identified in *vp5 and Vp5* embryos during maize seed maturation.

**Additional file 4: Table S4.** Genes identified mediumpurple3 module.

**Additional file 5: Table S5.** Gene-specific primers used in RT-qPCR.

**Additional file 6: Fig. S1.** Heat map of correlation coefficient between *Vp5* and *vp5* samples.

**Additional file 7: Fig. S2.** A mature ear with white *vp5* kernels and yellow *Vp5* kernels separating from the mutation.

## Data Availability

The raw RNA-seq data files have been deposited in European Nucleotide Archive (ENA) (https://www.ebi.ac.uk/ena/browser/text-search?query=E-MTAB-11224) and are publicly available.

## Abbreviations

ABA: Abscisic acid
ANOVA: Analysis of variance
BR: Brassinosteroids
CER3: CERIFERUM 3
DAP: Days after pollination
DEGs: Differentially expressed genes
FPKM: Fragments per kilobase of exon model per million reads mapped
GO: Gene Ontology
GAPDH: Glyceraldehyde-3-phosphate dehydrogenase
GA: Gibberellin
HSPs: Heat shock proteins
IAA: Indole-3-acetic acid
KEGG: Kyoto Encyclopedia of Genes and Genomes
LEA: Late embryogenesis abundant
LC/MS: Liquid chromatography/mass spectrometry
MAPK: Mitogen-activated protein kinase
PCA: Principal components analysis
PDC: Pyruvate decarboxylase
RNA-Seq: RNA Sequencing
RT-qPCR: Reverse transcription quantitative PCR
TPI: Triose phosphate isomerase
UPLC/ESI-MS/MS: Ultrahigh-performance liquid chromatography coupled to electrospray ionization tandem spectrometry
vp5: viviparous-5
WGCNA: Weighted correlation network analysis
