# Supplementary material for: Transcriptome and co-expression network analyses of key genes and pathways associated with differential abscisic acid accumulation during maize seed maturation
Source: BMC Plant Biol. 2022 Jul 22;22:359. doi: 10.1186/s12870-022-03751-1 (PMC9308322; doi:10.1186/s12870-022-03751-1)
Supplement: Supplementary file 1 — Additional file 1: Table S1. Statistical analysis of RNA-seq data quality. [file 12870_2022_3751_MOESM1_ESM.docx]

**Supplemental Table 1** Statistical analysis of RNA-seq data quality.

| Samples | Cleans Reads (M) | Clean base (G) | GC Content (%) | ≥Q30 (*%*) |
| --- | --- | --- | --- | --- |
| 15 DAP *Vp5*  22 DAP *Vp5*  29 DAP *Vp5*  36 DAP *Vp5*  15 DAP *vp5*  22 DAP *vp5*  29 DAP *vp5*  36 DAP *vp5* | 48.06  47.84  48.08  47.92  47.72  48.01  48.15  47.96 | 7.0  6.94  7.0  6.99  6.92  6.98  7.01  6.97 | 53.28  55.18  55.68  54.90  52.78  54.05  55.34  54.11 | 96.16  96.03  94.38  94.32  95.13  96.35  96.38  96.45 |

Note: Clean reads: the number of clean reads obtained after filtering; Clean bases: the amount of sequencing obtained after filtering, the number of bases; Q30: the bases in the original data whose base mass value is greater than 30 account for the total base Percentage; GC: The sum of the number of G and C in the Clean bases as a percentage of the total number of bases.
