# Supplementary material for: Transcriptome and co-expression network analyses of key genes and pathways associated with differential abscisic acid accumulation during maize seed maturation
Source: BMC Plant Biol. 2022 Jul 22;22:359. doi: 10.1186/s12870-022-03751-1 (PMC9308322; doi:10.1186/s12870-022-03751-1)
Supplement: Supplementary file 2 — Additional file 2: Table S2. Comparison of RNA-seq data with reference genome. [file 12870_2022_3751_MOESM2_ESM.docx]

**Supplemental Table 2** Comparison of RNA-seq data with reference genome.

| Samples | Total reads | Total mapped | Uniquely mapped | Multiple mapped | Reads mapped to '+' | Reads mapped to '-' |
| --- | --- | --- | --- | --- | --- | --- |
| 15 DAP *Vp5* | 4805908 | 44312067  (92.2%) | 42652915  (92.2%) | 165915  (3.45%) | 21355200  (44.44%) | 21297715  (44.31%) |
| 22 DAP *Vp5* | 47840022 | 43560833  (91.06%) | 41927391  (87.64%) | 1633441  (3.41%) | 20975714  （43.85%） | 20951678  （43.8%） |
| 29 DAP *Vp5* | 48082865 | 43371657  (90.20%) | 41598134  (86.51%) | 1773522  (3.69%) | 20802328  (43.26%) | 20795806  (43.25%) |
| 36 DAP *Vp5* | 47923630 | 42756504  (89.92%) | 40914752  (85.38%) | 1841753  (3.84%) | 20477410  (42.73%) | 20437342  (42.64%) |
| 15 DAP *vp5* | 47721947 | 440807762  (92.38%) | 42503599  (89.06%) | 1584163  (3.32%) | 21276429  (44.58%) | 21227170  (44.48%) |
| 22 DAP *vp5* | 48009910 | 43716894  (91.06%) | 42049704  (87.58%) | 1667190  (3.47%) | 21040501  (43.83%) | 21009143  (43.76%) |
| 29 DAP *vp5* | 48157486 | 43457399  (90.24%) | 41714279  (86.62%) | 1743121  (3.62%) | 20860386  (43.32%) | 20853892  (43.30%) |
| 36 DAP *vp5* | 47957725 | 42790964  (89.23%) | 40900786  (85.28%) | 1889978  (3.94%) | 20463349  (42.67%) | 20437437  (42.61%) |

Note: Total reads: Cleansing of sequenced data after sequencing data; Total mapped: Statistics of the number of sequencing sequences that can be mapped to the genome; in general, if there is no contamination and the reference genome is selected appropriately. In this case, the percentage of this part of the data is greater than 70%; Multiple mapped: the number of sequencing sequences with multiple alignment positions on the reference sequence; Uniquely mapped: the number of sequencing sequences with unique alignment positions on the reference sequence; Reads map to '+', Reads map to '-': Sequencing sequences are aligned to the statistics of the positive and negative strands on the genome.
