# Supplementary material for: Transcriptome and co-expression network analyses of key genes and pathways associated with differential abscisic acid accumulation during maize seed maturation
Source: BMC Plant Biol. 2022 Jul 22;22:359. doi: 10.1186/s12870-022-03751-1 (PMC9308322; doi:10.1186/s12870-022-03751-1)
Supplement: Supplementary file 3 — Additional file 3: Table S3. DEGs identified in vp5 and Vp5 embryos during maize seed maturation. [file 12870_2022_3751_MOESM3_ESM.docx]

**Supplemental Table 3** DEGs identified in *vp5 and Vp5* embryos during maize seed maturation**.**

**A**. DEGs by *vp5 vs.Vp5* at 15 DAP

| **Gene ID** | **Fold change** | **log2fold-change** | ***P* adjusted** | **Up/down** | **Note** |
| --- | --- | --- | --- | --- | --- |
| *Zm00001d000316* | 0.444455 | -1.16989 | 1.57E-05 | Down | NADP-dependent malic enzyme (chloroplastic) |
| *Zm00001d003112* | 0.372438 | -1.42493 | 4.19E-09 | Down | Leucine-rich repeat family protein |
| *Zm00001d003247* | 0.482324 | -1.05192 | 0.001204 | Down | Malate synthase1 |
| *Zm00001d004006* | 0.387182 | -1.36892 | 1.35E-09 | Down | SNF1-related protein kinase regulatory subunit gamma-1 |
| *Zm00001d007753* | 0.327862 | -1.60884 | 1.44E-09 | Down | Eukaryotic aspartyl protease family protein |
| *Zm00001d007765* | 0.497874 | -1.00615 | 1.01E-07 | Down | Probable prolyl 4-hydroxylase 12 |
| *Zm00001d008241* | 0.484243 | -1.0462 | 3.45E-07 | Down | Embryonic protein DC-8 |
| *Zm00001d009118* | 0.465955 | -1.10174 | 2.82E-07 | Down | Adenine nucleotide alpha hydrolases-like superfamily protein |
| *Zm00001d012221* | 0.46311 | -1.11057 | 1.48E-11 | Down | Stearoyl-acyl-carrier-protein desaturase9 |
| *Zm00001d012304* | 0.494832 | -1.01499 | 0.001689 | Down | Putative cytochrome P450 superfamily protein |
| *Zm00001d012821* | 0.496601 | -1.00984 | 2.02E-09 | Down | Probable inactive poly [ADP-ribose] polymerase SRO1 |
| *Zm00001d013099* | 0.484259 | -1.04615 | 3.86E-05 | Down | Probable carboxylesterase 15 |
| *Zm00001d013193* | 0.426972 | -1.22779 | 8.41E-12 | Down | CCG-binding protein 1 |
| *Zm00001d014166* | 0.438139 | -1.19054 | 2.09E-05 | Down | Putative calcium-transporting ATPase 13 plasma membrane-type |
| *Zm00001d014852* | 0.426844 | -1.22822 | 2.13E-05 | Down | Ser/Thr-rich protein T10 in DGCR region |
| *Zm00001d016128* | 0.245658 | -2.02527 | 5.30E-33 | Down |  |
| *Zm00001d016705* | 0.277462 | -1.84964 | 1.13E-11 | Down | ATPase inhibitor |
| *Zm00001d017140* | 0.484508 | -1.04541 | 5.86E-05 | Down | GDSL esterase/lipase |
| *Zm00001d017274* | 0.457297 | -1.1288 | 1.96E-11 | Down | Phenylalanine ammonia-lyase |
| *Zm00001d017547* | 0.319307 | -1.64699 | 7.23E-14 | Down | Dehydrin COR410 |
| *Zm00001d018342* | 0.416536 | -1.26349 | 2.00E-09 | Down | Trehalose-6-phosphate synthase11 |
| *Zm00001d018966* | 0.324386 | -1.62422 | 7.05E-11 | Down | Acidic endochitinase |
| *Zm00001d020396* | 0.456991 | -1.12976 | 2.21E-07 | Down | Trehalose-6-phosphate synthase13 |
| *Zm00001d021006* | 0.471478 | -1.08474 | 5.28E-07 | Down | Mtd1 |
| *Zm00001d022467* | 0.497043 | -1.00856 | 0.000384 | Down | Anthocyanidin 3-O-glucosyltransferase |
| *Zm00001d023664* | 0.46204 | -1.11391 | 3.31E-05 | Down | ABA-responsive protein |
| *Zm00001d023694* | 0.447245 | -1.16086 | 1.97E-09 | Down | 2-oxoisovalerate dehydrogenase subunit alpha 2 mitochondrial |
| *Zm00001d024778* | 0.44265 | -1.17576 | 1.17E-10 | Down | Hydrophobic protein RCI2B |
| *Zm00001d024839* | 0.324574 | -1.62338 | 1.08E-09 | Down | Glutathione S-transferase2 |
| *Zm00001d024963* | 0.406044 | -1.30029 | 8.41E-12 | Down | Glutathione S-transferase GSTU6 |
| *Zm00001d025229* | 0.417894 | -1.25879 | 5.28E-07 | Down |  |
| *Zm00001d025401* | 0.414754 | -1.26967 | 2.26E-06 | Down | Abscisic acid stress ripening5 |
| *Zm00001d026632* | 0.363687 | -1.45923 | 2.56E-11 | Down | Stem-specific protein TSJT1 |
| *Zm00001d027742* | 0.393115 | -1.34698 | 2.78E-07 | Down |  |
| *Zm00001d027924* | 0.421142 | -1.24762 | 1.02E-12 | Down | AP2-EREBP transcription factor |
| *Zm00001d028588* | 0.482747 | -1.05066 | 0.000301 | Down | Fes1B |
| *Zm00001d028647* | 0.423425 | -1.23982 | 1.82E-07 | Down | Glyoxalase family protein superfamily |
| *Zm00001d029062* | 0.416299 | -1.26431 | 4.72E-06 | Down | Vicilin-like seed storage protein |
| *Zm00001d030915* | 0.432103 | -1.21055 | 2.61E-05 | Down |  |
| *Zm00001d031315* | 0.346607 | -1.52863 | 1.60E-15 | Down | Transducin/WD40 repeat-like superfamily protein |
| *Zm00001d032519* | 0.378649 | -1.40107 | 2.54E-11 | Down | Expressed protein |
| *Zm00001d032552* | 0.422118 | -1.24428 | 1.77E-05 | Down | Protein NEOXANTHIN-DEFICIENT 1 |
| *Zm00001d032810* | 0.303186 | -1.72173 | 2.34E-13 | Down | CHY-type/CTCHY-type/RING-type Zinc finger protein |
| *Zm00001d032825* | 0.480173 | -1.05837 | 0.000743 | Down |  |
| *Zm00001d033714* | 0.401248 | -1.31744 | 3.65E-06 | Down | Regulator of Vps4 activity in the MVB pathway protein |
| *Zm00001d034501* | 0.478683 | -1.06286 | 2.04E-05 | Down | AAA-ATPase ASD (mitochondrial) |
| *Zm00001d035000* | 0.498263 | -1.00502 | 6.07E-05 | Down | Seed maturation protein |
| *Zm00001d035683* | 0.491389 | -1.02506 | 1.10E-06 | Down | Subtilisin-chymotrypsin inhibitor CI-1B |
| *Zm00001d037656* | 0.322643 | -1.63199 | 1.10E-12 | Down | Xylanase inhibitor protein 1 |
| *Zm00001d037684* | 0.490462 | -1.02779 | 0.001165 | Down |  |
| *Zm00001d038003* | 0.425959 | -1.23121 | 4.18E-08 | Down |  |
| *Zm00001d038558* | 0.438899 | -1.18804 | 0.000112 | Down | Cystatin3 |
| *Zm00001d038870* | 0.3734 | -1.42121 | 1.48E-08 | Down | Late embryogenesis abundant protein group 3 |
| *Zm00001d040787* | 0.316358 | -1.66037 | 9.82E-10 | Down | Abscisic stress-ripening protein 1 |
| *Zm00001d042114* | 0.339271 | -1.55949 | 4.35E-12 | Down |  |
| *Zm00001d042779* | 0.409708 | -1.28733 | 1.68E-05 | Down | Protein ABSCISIC ACID-INSENSITIVE 5 |
| *Zm00001d042848* | 0.43932 | -1.18666 | 8.05E-05 | Down | Laccase-7 |
| *Zm00001d043121* | 0.499701 | -1.00086 | 0.000324 | Down | Osmotin-like protein OSM34 |
| *Zm00001d043525* | 0.381191 | -1.39141 | 2.21E-07 | Down | Oxidative stress 3 |
| *Zm00001d043540* | 0.4874 | -1.03682 | 0.000278 | Down |  |
| *Zm00001d044022* | 0.2884 | -1.79386 | 5.14E-14 | Down | Seed maturation protein PM41 |
| *Zm00001d044908* | 0.466152 | -1.10113 | 0.000227 | Down | 12-oxo-phytodienoic acid reductase1 |
| *Zm00001d044915* | 0.34215 | -1.5473 | 8.06E-12 | Down |  |
| *Zm00001d045390* | 0.415927 | -1.2656 | 7.49E-13 | Down | Early nodulin-related |
| *Zm00001d045391* | 0.288894 | -1.79139 | 3.98E-30 | Down | Early nodulin-related |
| *Zm00001d045392* | 0.286856 | -1.8016 | 2.95E-35 | Down | Early nodulin 93 |
| *Zm00001d047480* | 0.446859 | -1.16211 | 0.000192 | Down | Beta-amylase |
| *Zm00001d047492* | 0.468053 | -1.09526 | 7.92E-07 | Down | Heavy metal transport/detoxification superfamily protein |
| *Zm00001d047951* | 0.470229 | -1.08857 | 4.15E-10 | Down | Senescence/dehydration-associated protein-related |
| *Zm00001d047993* | 0.435512 | -1.19922 | 6.54E-05 | Down | Probable inactive poly [ADP-ribose] polymerase SRO1 |
| *Zm00001d048307* | 0.453255 | -1.1416 | 1.29E-06 | Down |  |
| *Zm00001d049244* | 0.376084 | -1.41087 | 9.52E-07 | Down | Orphans transcription factor |
| *Zm00001d049889* | 0.477237 | -1.06722 | 0.000641 | Down | Putative AP2/EREBP transcription factor superfamily protein |
| *Zm00001d050336* | 0.3532 | -1.50144 | 1.12E-12 | Down | Methylcrotonoyl-CoA carboxylase beta chain mitochondrial |
| *Zm00001d050346* | 0.36742 | -1.4445 | 4.17E-07 | Down | Annexin |
| *Zm00001d051938* | 0.269163 | -1.89345 | 4.52E-18 | Down | Cinnamoyl- CoA reductase 1 |
| *Zm00001d052060* | 0.451403 | -1.14751 | 2.85E-08 | Down | Trehalose-6-phosphate synthase10 |
| *Zm00001d052435* | 0.42376 | -1.23868 | 3.06E-06 | Down | Probable peptide/nitrate transporter |
| *Zm00001d054093* | 0.311344 | -1.68342 | 7.71E-10 | Down | Senescence-associated protein DIN1 |
| *Zm00001d007489* | 2.500187 | 1.322036 | 7.71E-10 | Up | Zinc-finger domain of monoamine-oxidase A repressor R1 |
| *Zm00001d008393* | 2.04929 | 1.035124 | 0.000465 | Up | Putative aldehyde dehydrogenase family protein |
| *Zm00001d008797* | 2.121795 | 1.085285 | 0.000641 | Up | E3 ubiquitin-protein ligase CCNB1IP1 homolog |
| *Zm00001d009374* | 2.170261 | 1.117869 | 1.43E-06 | Up | DNA replication licensing factor MCM4 |
| *Zm00001d030614* | 2.01689 | 1.012133 | 1.48E-08 | Up | DNA replication licensing factor MCM7 |
| *Zm00001d035590* | 5.442695 | 2.444321 | 7.46E-21 | Up | DUF1639 family protein |
| *Zm00001d035963* | 2.216014 | 1.147967 | 1.50E-06 | Up |  |
| *Zm00001d045354* | 2.341535 | 1.227455 | 5.68E-08 | Up | CDT1-like protein A (chloroplastic) |

**B**. DEGs by *vp5* vs.*Vp5* at 22 DAP.

| **Gene ID** | **Fold change** | **log2fold-change** | ***P* adjusted** | **Up/down** | **Note** |
| --- | --- | --- | --- | --- | --- |
| *Zm00001d008812* | 0.448714 | -1.15613 | 4.13E-23 | Down | C3H47 C3H type transcription factor |
| *Zm00001d010800* | 0.481856 | -1.05333 | 2.46E-16 | Down | Zinc finger CCCH domain-containing protein 54 |
| *Zm00001d021006* | 0.383203 | -1.38382 | 1.02E-22 | Down | MTD1 |
| *Zm00001d026632* | 0.36188 | -1.46641 | 4.13E-23 | Down | Stem-specific protein TSJT1 |
| *Zm00001d027924* | 0.329503 | -1.60164 | 2.50E-73 | Down | AP2-EREBP transcription factor |
| *Zm00001d029052* | 0.489633 | -1.03023 | 9.38E-12 | Down |  |
| *Zm00001d039495* | 0.445764 | -1.16565 | 8.22E-19 | Down | Zinc finger CCCH domain-containing protein 23 |
| *Zm00001d042541* | 0.481339 | -1.05487 | 1.42E-16 | Down | Lipoxygenase2 |
| *Zm00001d044915* | 0.440531 | -1.18268 | 3.29E-22 | Down |  |

**C**. DEGs by *vp5 vs. Vp5* at 29 DAP.

| **Gene ID** | **Fold change** | **log2fold-change** | ***P* adjusted** | **Up/down** | **Note** |
| --- | --- | --- | --- | --- | --- |
| *Zm00001d002160* | 0.49051 | -1.02765 | 5.79E-09 | Down | Wound-responsive family protein |
| *Zm00001d003554* | 0.487851 | -1.03549 | 9.21E-10 | Down | 22.0 kDa heat shock protein |
| *Zm00001d005793* | 0.483553 | -1.04825 | 1.79E-08 | Down | Zein-beta |
| *Zm00001d006467* | 0.443263 | -1.17376 | 6.45E-13 | Down | Adenosine 5'-phosphosulfate reductase-like2 |
| *Zm00001d007820* | 0.428858 | -1.22143 | 7.05E-07 | Down | Plant-specific domain TIGR01615 family protein |
| *Zm00001d008808* | 0.471528 | -1.08458 | 8.84E-05 | Down | Putative MYB DNA-binding domain superfamily protein |
| *Zm00001d012323* | 0.166933 | -2.58266 | 1.20E-26 | Down |  |
| *Zm00001d013296* | 0.327183 | -1.61183 | 4.04E-24 | Down | ATP sulfurylase 1 (chloroplastic) |
| *Zm00001d013412* | 0.41633 | -1.2642 | 8.37E-08 | Down | Histidine kinase 4 |
| *Zm00001d014158* | 0.401795 | -1.31547 | 3.92E-09 | Down | Expressed protein |
| *Zm00001d014549* | 0.311495 | -1.68272 | 1.44E-11 | Down | Cwf15 / Cwc15 cell cycle control family protein |
| *Zm00001d017545* | 0.468158 | -1.09493 | 1.18E-08 | Down | SEC14 cytosolic factor family protein / phosphoglyceride transfer family protein |
| *Zm00001d019155* | 0.285805 | -1.80689 | 3.17E-20 | Down | Zein-alpha A20 |
| *Zm00001d020591* | 0.369921 | -1.43471 | 2.98E-14 | Down | Zein-gamma 50 kD |
| *Zm00001d021596* | 0.450135 | -1.15157 | 1.41E-05 | Down | Adenosine 5'-phosphosulfate reductase-like1 |
| *Zm00001d025533* | 0.488679 | -1.03304 | 0.000120191 | Down | NAD(P)-linked oxidoreductase superfamily protein |
| *Zm00001d029708* | 0.368742 | -1.43932 | 5.58E-30 | Down | Glutathione transferase30 |
| *Zm00001d031861* | 0.472854 | -1.08053 | 5.31E-13 | Down | Dehydration-responsive element-binding protein 2G |
| *Zm00001d032090* | 0.487802 | -1.03563 | 7.41E-10 | Down | NADPH--cytochrome P450 reductase 2 |
| *Zm00001d033539* | 0.348844 | -1.51935 | 1.20E-14 | Down | Gamma-glutamyl peptidase 1 |
| *Zm00001d033981* | 0.467008 | -1.09848 | 1.97E-09 | Down | ATP sulfurylase1 |
| *Zm00001d038642* | 0.432203 | -1.21022 | 5.26E-12 | Down | Protein SULFUR DEFICIENCY-INDUCED 1 |
| *Zm00001d041681* | 0.480728 | -1.05671 | 0.000189843 | Down | Hat transposon superfamily protein |
| *Zm00001d042504* | 0.493388 | -1.0192 | 0.000103486 | Down | Patatin-like protein 2 |
| *Zm00001d042661* | 0.418101 | -1.25808 | 2.52E-16 | Down | Serine hydroxymethyltransferase 7 |
| *Zm00001d042780* | 0.449408 | -1.1539 | 5.09E-12 | Down | Alpha/beta-Hydrolases superfamily protein |
| *Zm00001d042801* | 0.409059 | -1.28962 | 2.50E-08 | Down | GABA transporter 1 |
| *Zm00001d048469* | 0.465725 | -1.10245 | 4.00E-06 | Down | Hydroxylase5 |
| *Zm00001d048809* | 0.393662 | -1.34497 | 3.03E-12 | Down | Zein protein 22.1 |
| *Zm00001d048817* | 0.437061 | -1.19409 | 1.52E-06 | Down | Zein protein3 |
| *Zm00001d048848* | 0.3316 | -1.59248 | 3.86E-13 | Down | Zein-alpha 19B1 |
| *Zm00001d048849* | 0.369706 | -1.43555 | 2.19E-11 | Down | Putative uncharacterized protein |
| *Zm00001d048850* | 0.457735 | -1.12742 | 3.97E-05 | Down | Zein-alpha PMS1 |
| *Zm00001d048851* | 0.453876 | -1.13963 | 3.79E-05 | Down | Floury4 |
| *Zm00001d049243* | 0.408661 | -1.29102 | 9.65E-10 | Down | Floury2 |
| *Zm00001d050577* | 0.430641 | -1.21544 | 3.57E-07 | Down | Sugars will eventually be exported transporter15a |
| *Zm00001d001862* | 2.188185 | 1.129735 | 1.15E-08 | Up |  |
| *Zm00001d002744* | 2.013774 | 1.009902 | 5.90E-05 | Up | Dehydration-responsive element-binding protein 1C |
| *Zm00001d002899* | 2.068268 | 1.048423 | 7.05E-07 | Up | Peroxidase 12 |
| *Zm00001d003272* | 2.00254 | 1.001831 | 0.000381784 | Up | Nucleotide binding protein |
| *Zm00001d003533* | 2.057229 | 1.040703 | 1.11E-09 | Up | Lipoxygenase |
| *Zm00001d006171* | 2.305746 | 1.205234 | 7.97E-06 | Up | Protein DETOXIFICATION 50 |
| *Zm00001d006446* | 2.304627 | 1.204533 | 2.20E-12 | Up | Salmon silk2 |
| *Zm00001d006828* | 2.18989 | 1.130858 | 3.59E-05 | Up | Protein LONGIFOLIA 2 |
| *Zm00001d007175* | 2.167857 | 1.11627 | 2.78E-07 | Up | Expressed protein |
| *Zm00001d007764* | 2.541106 | 1.345456 | 1.34E-12 | Up | Hydroxyproline O-galactosyltransferase GALT4 |
| *Zm00001d008266* | 2.100602 | 1.070803 | 2.47E-09 | Up | Peroxidase 24 |
| *Zm00001d011208* | 2.011336 | 1.008154 | 1.84E-13 | Up | 1-Aminocyclopropane-1-carboxylate oxidase 1 |
| *Zm00001d013046* | 2.135002 | 1.094238 | 1.44E-08 | Up | Lipid phosphate phosphatase delta |
| *Zm00001d016225* | 2.034972 | 1.025009 | 9.50E-09 | Up | U-box domain-containing protein 17 |
| *Zm00001d016719* | 2.13359 | 1.093283 | 9.64E-05 | Up | U-box domain-containing protein 29 |
| *Zm00001d017699* | 2.114037 | 1.080001 | 0.000113312 | Up | Probable xyloglucan endotransglucosylase/hydrolase protein 30 |
| *Zm00001d017762* | 2.065185 | 1.046271 | 4.47E-08 | Up | Abscisic acid 8'-hydroxylase1 |
| *Zm00001d017983* | 2.296297 | 1.199309 | 4.33E-09 | Up | Formin homologue 4 |
| *Zm00001d018717* | 2.007577 | 1.005455 | 0.000208367 | Up |  |
| *Zm00001d018807* | 2.037236 | 1.026613 | 2.19E-11 | Up | Leucine-rich repeat receptor-like serine/threonine-protein kinase |
| *Zm00001d019505* | 2.211295 | 1.144892 | 4.91E-14 | Up | SNAP25 homologous protein SNAP33 |
| *Zm00001d020257* | 2.358089 | 1.237618 | 4.36E-06 | Up | Heavy metal transport/detoxification superfamily protein |
| *Zm00001d020492* | 2.180431 | 1.124613 | 4.32E-05 | Up | Probable WRKY transcription factor 40 |
| *Zm00001d021026* | 3.045453 | 1.606657 | 1.79E-14 | Up | Protein capI |
| *Zm00001d021971* | 2.090102 | 1.063573 | 0.000113312 | Up | Triacylglycerol lipase |
| *Zm00001d022017* | 2.038127 | 1.027244 | 1.63E-14 | Up | Probable indole-3-acetic acid-amido synthetase GH3.1 |
| *Zm00001d022041* | 2.100256 | 1.070565 | 3.07E-14 | Up |  |
| *Zm00001d022245* | 2.927411 | 1.549626 | 8.02E-13 | Up | IQ domain-containing protein IQM2 |
| *Zm00001d022416* | 2.393884 | 1.259353 | 2.24E-16 | Up | Early response to dehydration 15-like protein |
| *Zm00001d023385* | 2.143744 | 1.100132 | 4.05E-09 | Up | Phytosulfokine4 |
| *Zm00001d023400* | 2.218439 | 1.149545 | 8.25E-10 | Up | Putative cytochrome P450 superfamily protein |
| *Zm00001d024934* | 2.138791 | 1.096795 | 2.47E-09 | Up | Probable beta-14-xylosyltransferase IRX9H |
| *Zm00001d025166* | 2.593768 | 1.375049 | 9.21E-16 | Up | Putative quinone-oxidoreductase homolog chloroplastic |
| *Zm00001d027991* | 2.119811 | 1.083935 | 7.97E-06 | Up | HB-type transcription factor |
| *Zm00001d028093* | 2.38079 | 1.251441 | 1.23E-13 | Up | Calcium-transporting ATPase 2 plasma membrane-type |
| *Zm00001d029051* | 2.036475 | 1.026074 | 3.10E-06 | Up | Expressed protein |
| *Zm00001d029853* | 2.027369 | 1.019609 | 2.90E-07 | Up | Proline oxidase |
| *Zm00001d031657* | 2.113291 | 1.079491 | 0.000127616 | Up | Probable inactive purple acid phosphatase 27 |
| *Zm00001d031796* | 3.419408 | 1.773747 | 1.23E-13 | Up | AP2 domain containing protein |
| *Zm00001d032287* | 2.956719 | 1.563997 | 1.33E-09 | Up |  |
| *Zm00001d033019* | 2.043565 | 1.031088 | 0.00030379 | Up | Tpd1 |
| *Zm00001d033020* | 2.106074 | 1.074556 | 1.04E-11 | Up | Cytochrome P450 CYP78A1 |
| *Zm00001d033050* | 2.37343 | 1.246974 | 4.00E-08 | Up | ZIM motif family protein |
| *Zm00001d034205* | 2.030396 | 1.021761 | 1.97E-16 | Up | Probable xyloglucan glycosyltransferase 12 |
| *Zm00001d034494* | 2.694377 | 1.429952 | 1.04E-15 | Up | Actin cross-linking protein |
| *Zm00001d035035* | 2.48927 | 1.315723 | 2.63E-08 | Up | Putative CRINKLY4-like receptor protein kinase family protein |
| *Zm00001d041608* | 2.096609 | 1.068058 | 3.96E-11 | Up | Phosphatidylinositol:ceramide inositolphosphotransferase 1 |
| *Zm00001d042063* | 2.755754 | 1.462447 | 6.14E-12 | Up |  |
| *Zm00001d042449* | 4.119857 | 2.042594 | 6.54E-18 | Up | BCL-2 binding anthanogene-1 |
| *Zm00001d042627* | 2.233088 | 1.15904 | 3.51E-20 | Up | UDP-glucuronate:xylan alpha-glucuronosyltransferase 1 |
| *Zm00001d042799* | 2.646102 | 1.403868 | 2.12E-16 | Up | G10 family protein |
| *Zm00001d042800* | 2.434303 | 1.283509 | 1.36E-10 | Up | Protein BUD31-like protein 1 |
| *Zm00001d042975* | 3.386127 | 1.759636 | 3.17E-20 | Up | Serine carboxypeptidase-like 27 |
| *Zm00001d043196* | 2.744286 | 1.456431 | 4.57E-21 | Up | DNA glycosylase superfamily protein |
| *Zm00001d043935* | 2.439922 | 1.286835 | 8.25E-10 | Up | Ras-related protein RABA1f |
| *Zm00001d043989* | 2.633111 | 1.396769 | 3.03E-09 | Up | F-box/kelch-repeat protein |
| *Zm00001d047055* | 2.175297 | 1.121213 | 5.55E-05 | Up | Formin-like protein 18 |
| *Zm00001d047402* | 2.627605 | 1.393749 | 4.65E-22 | Up | Putative calmodulin-binding family protein |
| *Zm00001d047931* | 2.076682 | 1.05428 | 6.12E-05 | Up | 3-ketoacyl-coa synthase |
| *Zm00001d049091* | 2.365123 | 1.241915 | 3.47E-08 | Up |  |
| *Zm00001d049510* | 2.145767 | 1.101493 | 5.60E-05 | Up | Putative cellulose synthase-like family protein |
| *Zm00001d049643* | 2.00902 | 1.006492 | 4.65E-06 | Up | Zinc finger (C3HC4-type RING finger) family protein |
| *Zm00001d051981* | 2.040726 | 1.029083 | 2.46E-20 | Up | GATA transcription factor 8 |
| *Zm00001d052053* | 2.060401 | 1.042925 | 0.0001264 | Up |  |
| *Zm00001d052189* | 2.153541 | 1.106711 | 7.53E-05 | Up | Putative RING zinc finger domain superfamily protein |
| *Zm00001d052206* | 2.1773 | 1.12254 | 7.77E-07 | Up | Protein EXORDIUM |
| *Zm00001d052530* | 3.276055 | 1.71196 | 1.97E-16 | Up | Cytochrome P450 94C1 |
| *Zm00001d053087* | 2.250786 | 1.170429 | 9.70E-06 | Up | G-type lectin S-receptor-like serine/threonine-protein kinase SD2-5 |
| *Zm00001d053818* | 3.412303 | 1.770746 | 1.57E-29 | Up | Protein kinase superfamily protein |

**D**. DEGs by *vp5* vs. *Vp5* at 36 DAP.

| **Gene ID** | **Fold change** | **log2fold-change** | ***P* adjusted** | **Up/down** | **Note** |
| --- | --- | --- | --- | --- | --- |
| *Zm00001d000133* | 0.374078328 | -1.41858771 | 5.49E-51 | Down | Acetyl-coenzyme A synthetase (chloroplastic/glyoxysomal) |
| *Zm00001d000179* | 0.489173329 | -1.031582348 | 0.002829673 | Down | AP2-EREBP-transcription factor 1 |
| *Zm00001d000195* | 0.497775838 | -1.006431891 | 4.50E-05 | Down | Putative MATE efflux family protein |
| *Zm00001d000203* | 0.470928358 | -1.086420494 | 0.019473876 | Down | Patatin-like protein 6 |
| *Zm00001d000222* | 0.489334001 | -1.031108566 | 8.94E-18 | Down | Sugars will eventually be exported transporter1a |
| *Zm00001d000237* | 0.378294689 | -1.402417573 | 1.38E-38 | Down | Cis-zeatin O-glucosyltransferase1 |
| *Zm00001d000238* | 0.363133389 | -1.461428509 | 5.56E-12 | Down | Growth-regulating factor 6 |
| *Zm00001d000288* | 0.455272026 | -1.135199277 | 1.74E-06 | Down | Auxin-responsive protein IAA29 |
| *Zm00001d000316* | 0.429505549 | -1.219251325 | 7.13E-12 | Down | NADP-dependent malic enzyme (chloroplastic) |
| *Zm00001d001766* | 0.390328677 | -1.357238636 | 8.76E-07 | Down |  |
| *Zm00001d002025* | 0.414660598 | -1.269997129 | 6.58E-39 | Down | AP2-like ethylene-responsive transcription factor AIL5 |
| *Zm00001d002071* | 0.25898591 | -1.949054483 | 5.79E-07 | Down | DNA topoisomerase 2 |
| *Zm00001d002287* | 0.214444616 | -2.221323001 | 5.00E-10 | Down | LRR receptor-like serine/threonine-protein kinase FLS2 |
| *Zm00001d002304* | 0.43057901 | -1.215650102 | 0.003276403 | Down | Auxin responsive protein |
| *Zm00001d002358* | 0.492946086 | -1.020498228 | 3.11E-18 | Down | Necrotic4 |
| *Zm00001d002410* | 0.392295274 | -1.34998814 | 8.69E-26 | Down | Probable xyloglucan endotransglucosylase/hydrolase protein 21 |
| *Zm00001d002520* | 0.386888904 | -1.370008743 | 1.89E-05 | Down | Osjnba0013k16.16 protein |
| *Zm00001d002535* | 0.420483382 | -1.249879312 | 6.98E-11 | Down | RING-H2 finger protein ATL80 |
| *Zm00001d002601* | 0.447900784 | -1.158748903 | 5.97E-35 | Down | Histone H1-3 |
| *Zm00001d002690* | 0.486552683 | -1.039332069 | 1.63E-27 | Down | Plasma membrane intrinsic protein1 |
| *Zm00001d002743* | 0.20486966 | -2.287221746 | 6.77E-14 | Down | Zinc ion binding protein |
| *Zm00001d002759* | 0.329881672 | -1.599979469 | 5.84E-21 | Down | Nudix hydrolase 8 |
| *Zm00001d002847* | 0.390438392 | -1.356833176 | 7.94E-06 | Down | (+)-Neomenthol dehydrogenase |
| *Zm00001d002893* | 0.456972148 | -1.129821858 | 1.93E-05 | Down | WAT1-related protein |
| *Zm00001d002960* | 0.422528724 | -1.242878672 | 2.92E-05 | Down | Kunitz trypsin inhibitor 1 |
| *Zm00001d003110* | 0.449218167 | -1.15451182 | 0.002155612 | Down | Calmodulin-binding receptor-like cytoplasmic kinase 3 |
| *Zm00001d003151* | 0.410376538 | -1.284979844 | 0.001882109 | Down | N-acetyltransferase ESCO1 |
| *Zm00001d003190* | 0.476974403 | -1.06801625 | 1.64E-34 | Down | Endochitinase A |
| *Zm00001d003287* | 0.322345741 | -1.633319175 | 2.08E-36 | Down | Nitrate transport4 |
| *Zm00001d003669* | 0.407106666 | -1.296521251 | 0.00158605 | Down |  |
| *Zm00001d003690* | 0.315324658 | -1.665090104 | 9.56E-11 | Down |  |
| *Zm00001d003861* | 0.369177414 | -1.437613803 | 1.11E-08 | Down | Potassium high-affinity transporter |
| *Zm00001d003931* | 0.491202423 | -1.025610418 | 0.000691003 | Down | Acr4 |
| *Zm00001d003981* | 0.29637149 | -1.754521422 | 9.69E-22 | Down | Drought-induced protein 1 |
| *Zm00001d004006* | 0.44659913 | -1.162947654 | 2.03E-35 | Down | SNF1-related protein kinase regulatory subunit gamma-1 |
| *Zm00001d004086* | 0.471990567 | -1.083170068 | 5.97E-09 | Down | Probable WRKY transcription factor 11 |
| *Zm00001d004248* | 0.314890386 | -1.667078386 | 6.11E-05 | Down | UDP-glycosyltransferase 85A7 |
| *Zm00001d004524* | 0.48503733 | -1.043832308 | 0.027306687 | Down |  |
| *Zm00001d004640* | 0.486430844 | -1.039693383 | 0.024704344 | Down | Sec23/Sec24 protein transport family protein |
| *Zm00001d004897* | 0.311919827 | -1.680752835 | 3.28E-23 | Down | Basic-leucine zipper (BZIP) transcription factor family protein |
| *Zm00001d004908* | 0.415410791 | -1.267389402 | 0.001037308 | Down | Expressed protein |
| *Zm00001d005306* | 0.496905585 | -1.008956339 | 0.001303235 | Down | Dynamin-related protein 1E |
| *Zm00001d005609* | 0.390092067 | -1.358113434 | 1.46E-15 | Down | Protein phosphatase homolog7 |
| *Zm00001d005656* | 0.27263177 | -1.874974405 | 4.35E-13 | Down | Heavy metal transport/detoxification superfamily protein |
| *Zm00001d005659* | 0.478852122 | -1.062347901 | 1.36E-07 | Down |  |
| *Zm00001d005698* | 0.391594495 | -1.352567611 | 5.61E-08 | Down | An17 |
| *Zm00001d005748* | 0.423685257 | -1.238935167 | 5.71E-19 | Down | CIPK-like protein 1 |
| *Zm00001d005804* | 0.434718809 | -1.201845576 | 6.64E-11 | Down | Auxin-repressed protein |
| *Zm00001d005849* | 0.371404268 | -1.4289377 | 2.08E-11 | Down | Histone H3 K4-specific methyltransferase SET7/9 family protein |
| *Zm00001d005945* | 0.477491793 | -1.06645216 | 5.96E-07 | Down |  |
| *Zm00001d006016* | 0.283202102 | -1.820096123 | 4.49E-31 | Down | Multiple stress-responsive zinc-finger protein ISAP1 |
| *Zm00001d006211* | 0.437460524 | -1.192775258 | 0.000419081 | Down | Protein STAY-GREEN 1 (chloroplastic) |
| *Zm00001d006254* | 0.393457647 | -1.345719746 | 3.96E-11 | Down | Serine/threonine protein phosphatase 2A 59 kDa regulatory subunit B zeta isoform |
| *Zm00001d006256* | 0.392664775 | -1.348629912 | 4.55E-22 | Down | Syringolide-induced protein 14-1-1 |
| *Zm00001d006601* | 0.401110177 | -1.317929524 | 3.01E-06 | Down | Cotton fiber expressed protein 1 like |
| *Zm00001d006753* | 0.406330909 | -1.299272982 | 6.28E-31 | Down | Indole-3-acetic acid amido synthetase |
| *Zm00001d006883* | 0.409900307 | -1.286655024 | 1.54E-08 | Down | Calmodulin binding protein |
| *Zm00001d006947* | 0.365717011 | -1.451200363 | 0.000470467 | Down | Cytochrome P450 CYP709C14 |
| *Zm00001d007079* | 0.464331665 | -1.106772427 | 5.22E-08 | Down | Phospholipase A2 family protein |
| *Zm00001d007097* | 0.415192859 | -1.268146465 | 1.75E-21 | Down | Protein early responsive to dehydration 15 |
| *Zm00001d007199* | 0.415429691 | -1.267323765 | 4.67E-05 | Down |  |
| *Zm00001d007420* | 0.397145465 | -1.332260564 | 9.33E-07 | Down |  |
| *Zm00001d007581* | 0.421838851 | -1.245236124 | 2.48E-05 | Down | Harpin binding protein1 |
| *Zm00001d007814* | 0.39114344 | -1.354230327 | 0.001948244 | Down | Protein detoxification 42 |
| *Zm00001d007830* | 0.325738683 | -1.618213038 | 8.98E-08 | Down | Leucine-rich repeat extensin-like protein 6 |
| *Zm00001d007876* | 0.499868047 | -1.000380787 | 8.24E-06 | Down | Nine-cis-epoxycarotenoid dioxygenase4 |
| *Zm00001d008288* | 0.384899011 | -1.37744813 | 4.70E-24 | Down |  |
| *Zm00001d008734* | 0.470051287 | -1.089109917 | 1.24E-12 | Down | Putative BZIP transcription factor superfamily protein |
| *Zm00001d008756* | 0.347071493 | -1.526695223 | 0.000164082 | Down | HXXXD-type acyl-transferase family protein |
| *Zm00001d008862* | 0.49444393 | -1.016121167 | 0.033259392 | Down | Cytokinin oxidase 5 |
| *Zm00001d008911* | 0.495097199 | -1.014216309 | 0.032920245 | Down | Sodium/calcium exchanger family protein / calcium-binding  EF hand family protein |
| *Zm00001d008913* | 0.428724889 | -1.221875923 | 0.005572024 | Down | Evolutionarily conserved C-terminal region 10 |
| *Zm00001d008987* | 0.455734173 | -1.133735541 | 0.012106372 | Down | Putative cytochrome P450 superfamily protein |
| *Zm00001d009118* | 0.356685234 | -1.487276603 | 2.95E-50 | Down | Adenine nucleotide alpha hydrolases-like superfamily protein |
| *Zm00001d009328* | 0.281422154 | -1.829192188 | 5.49E-10 | Down | Glycosyltransferase |
| *Zm00001d009382* | 0.410469989 | -1.28465135 | 3.53E-45 | Down | Late embryogenesis abundant protein Lea14-A |
| *Zm00001d009568* | 0.489332458 | -1.031113113 | 0.026321109 | Down | Glutaredoxin-C9 |
| *Zm00001d009971* | 0.468218369 | -1.094746561 | 0.012186006 | Down | Protein SHI related sequence 6 |
| *Zm00001d010136* | 0.433819665 | -1.204832643 | 0.002392336 | Down | Probable ferredoxin-4 (chloroplastic) |
| *Zm00001d010175* | 0.437514554 | -1.192597086 | 1.46E-06 | Down | Ethylene-responsive transcription factor ABR1 |
| *Zm00001d010201* | 0.353601098 | -1.499805339 | 6.58E-15 | Down | Transcription repressor MYB6 |
| *Zm00001d010375* | 0.310838298 | -1.685763828 | 2.08E-11 | Down | ATP-dependent 6-phosphofructokinase 4 chloroplastic |
| *Zm00001d010385* | 0.395974505 | -1.336520551 | 4.61E-09 | Down | Protein upstream of FLC |
| *Zm00001d010388* | 0.34781931 | -1.523590064 | 0.000293061 | Down |  |
| *Zm00001d010520* | 0.493817123 | -1.017951232 | 0.001071944 | Down | Glycerol-3-phosphate acyltransferase 5 |
| *Zm00001d010588* | 0.312404528 | -1.678512732 | 9.74E-12 | Down | Pyruvate decarboxylase1 |
| *Zm00001d010640* | 0.418482621 | -1.256760384 | 4.08E-13 | Down | F-box protein |
| *Zm00001d010662* | 0.441333734 | -1.180058069 | 0.000101869 | Down | SNF1-related protein kinase regulatory subunit beta-1 |
| *Zm00001d010812* | 0.413649418 | -1.273519543 | 0.00270362 | Down | Heat stress transcription factor A-4a |
| *Zm00001d010956* | 0.438243349 | -1.190195898 | 4.87E-05 | Down | Zinc finger CCCH domain-containing protein 29 |
| *Zm00001d011228* | 0.214168012 | -2.223185076 | 1.46E-12 | Down | Putative calmodulin-binding family protein |
| *Zm00001d011396* | 0.462937706 | -1.111110021 | 1.42E-07 | Down | GTPase activating protein Putative calcium-dependent lipid-binding (CALB domain) family protein |
| *Zm00001d011543* | 0.496073286 | -1.011374826 | 0.017642726 | Down | Grx_I1-glutaredoxin subgroup III |
| *Zm00001d011614* | 0.454202977 | -1.138590932 | 0.001140377 | Down | Putative MYB DNA-binding domain superfamily protein |
| *Zm00001d011649* | 0.492108137 | -1.022952724 | 0.020382776 | Down | UDP-glycosyltransferase 88A1 |
| *Zm00001d011734* | 0.14294424 | -2.80647561 | 1.02E-29 | Down | Inorganic pyrophosphatase 1 |
| *Zm00001d011735* | 0.206047318 | -2.278952408 | 2.53E-13 | Down | Serine acetyltransferase1 |
| *Zm00001d011819* | 0.357998105 | -1.481976143 | 7.84E-11 | Down | Chlorophyllide A oxygenase (chloroplastic) |
| *Zm00001d011832* | 0.276474455 | -1.854781907 | 4.61E-59 | Down | G-type lectin S-receptor-like serine/threonine-protein kinase SD2-5 |
| *Zm00001d011879* | 0.419735083 | -1.252449042 | 0.000648818 | Down |  |
| *Zm00001d011919* | 0.478750956 | -1.062652728 | 9.97E-10 | Down | Src2-like protein |
| *Zm00001d012103* | 0.457478481 | -1.128224213 | 3.05E-38 | Down | Aldolase2 |
| *Zm00001d012120* | 0.393451411 | -1.345742615 | 3.91E-06 | Down | KDEL-tailed cysteine endopeptidase CEP1 |
| *Zm00001d012169* | 0.482427589 | -1.051615682 | 0.00271769 | Down | RHOMBOID-like protein 13 |
| *Zm00001d012228* | 0.383096692 | -1.384219525 | 1.74E-13 | Down | 4,5-DOPA dioxygenase extradiol |
| *Zm00001d012255* | 0.398381264 | -1.327778298 | 0.00232988 | Down | Putative MYB DNA-binding domain superfamily protein |
| *Zm00001d012304* | 0.285880878 | -1.80651397 | 3.87E-07 | Down | Putative cytochrome P450 superfamily protein |
| *Zm00001d012321* | 0.371172563 | -1.429838026 | 2.52E-18 | Down | Nematode resistance protein-like HSPRO2 |
| *Zm00001d012407* | 0.244955449 | -2.029408709 | 1.27E-19 | Down | Triose phosphate isomerase5 |
| *Zm00001d012456* | 0.468727627 | -1.093178264 | 0.01056446 | Down | Jasmonate-regulated gene 21 |
| *Zm00001d012482* | 0.433131317 | -1.207123607 | 0.00745038 | Down | Putative WRKY DNA-binding domain superfamily protein |
| *Zm00001d012494* | 0.318589498 | -1.650229385 | 2.23E-15 | Down | U-box domain-containing protein 16 |
| *Zm00001d012518* | 0.326090894 | -1.616653939 | 3.18E-45 | Down | C3-bisphosphoglycerate-independent phosphoglycerate mutase |
| *Zm00001d012527* | 0.428456165 | -1.222780483 | 2.72E-15 | Down | NAC domain-containing protein 2 |
| *Zm00001d012663* | 0.453333604 | -1.141354987 | 0.004120623 | Down | Putative MATE efflux family protein |
| *Zm00001d012821* | 0.404775699 | -1.304805416 | 2.24E-05 | Down | Probable inactive poly [ADP-ribose] polymerase SRO1 |
| *Zm00001d012920* | 0.350799662 | -1.511280738 | 0.000329902 | Down | St225 |
| *Zm00001d012956* | 0.487415954 | -1.036774621 | 1.23E-24 | Down |  |
| *Zm00001d013003* | 0.38607705 | -1.373039298 | 3.01E-30 | Down | NAC domain-containing protein 2 |
| *Zm00001d013099* | 0.281451464 | -1.82904194 | 4.89E-25 | Down | Probable carboxylesterase 15 |
| *Zm00001d013326* | 0.29396056 | -1.766305491 | 2.75E-06 | Down |  |
| *Zm00001d013376* | 0.493434622 | -1.019069149 | 0.026044167 | Down | Probable LRR receptor-like serine/threonine-protein kinase |
| *Zm00001d013493* | 0.374714594 | -1.416135929 | 1.18E-10 | Down | Lipoxygenase5 |
| *Zm00001d013530* | 0.396613868 | -1.33419297 | 3.11E-09 | Down | 5'-AMP-activated protein kinase-related |
| *Zm00001d013635* | 0.389522329 | -1.360222064 | 2.49E-06 | Down | O-Glycosyl hydrolases family 17 protein |
| *Zm00001d013809* | 0.306678101 | -1.705202945 | 2.26E-26 | Down | Protein detoxification 40 |
| *Zm00001d013830* | 0.490751562 | -1.026935236 | 0.000651575 | Down | Cytochrome P450 family 76 subfamily C polypeptide 7 |
| *Zm00001d013909* | 0.495400679 | -1.013332248 | 0.010279569 | Down |  |
| *Zm00001d014007* | 0.485891617 | -1.041293553 | 0.009833695 | Down | Senescence regulator |
| *Zm00001d014063* | 0.408308365 | -1.29226897 | 5.77E-06 | Down | MDIS1-interacting receptor like kinase 2 |
| *Zm00001d014102* | 0.432695368 | -1.208576419 | 2.40E-10 | Down | Divalent ion symporter |
| *Zm00001d014481* | 0.445339071 | -1.167023905 | 6.48E-12 | Down | Dihydroflavonol-4-reductase |
| *Zm00001d014494* | 0.223375987 | -2.162453994 | 1.52E-29 | Down |  |
| *Zm00001d014703* | 0.489186218 | -1.031544335 | 0.006478557 | Down | AT3G13000 transcription factor |
| *Zm00001d014722* | 0.437962573 | -1.191120509 | 1.41E-08 | Down | Glycosyltransferase |
| *Zm00001d014748* | 0.244986012 | -2.029228719 | 3.27E-14 | Down | (Z)-3-hexen-1-ol acetyltransferase |
| *Zm00001d014765* | 0.463604097 | -1.10903478 | 7.45E-16 | Down | B-box zinc finger protein 20 |
| *Zm00001d015477* | 0.300760362 | -1.733313651 | 3.61E-13 | Down | Protein eceriferum 3 |
| *Zm00001d015515* | 0.283874669 | -1.816673977 | 3.36E-08 | Down | Probable WRKY transcription factor 40 |
| *Zm00001d015517* | 0.437995448 | -1.19101222 | 0.004905261 | Down | Protein polar localization during asymmetric division and redistribution |
| *Zm00001d015777* | 0.469926294 | -1.0894936 | 4.08E-21 | Down | Chloroplast small heat shock protein |
| *Zm00001d015844* | 0.372102274 | -1.426228886 | 2.99E-20 | Down | Alpha/beta hydrolase family protein |
| *Zm00001d015905* | 0.267362619 | -1.903130327 | 7.35E-34 | Down | Sugars will eventually be exported transporter4a |
| *Zm00001d016255* | 0.350152399 | -1.513945121 | 3.50E-34 | Down | Heat stress transcription factor C-1 |
| *Zm00001d016438* | 0.493858993 | -1.017828912 | 0.007302616 | Down | 3-ketoacyl-coa synthase |
| *Zm00001d016471* | 0.410246277 | -1.285437855 | 2.46E-20 | Down | Trans-cinnamate 4-monooxygenase |
| *Zm00001d016483* | 0.313160855 | -1.675024205 | 2.00E-27 | Down | MACPF domain-containing protein |
| *Zm00001d016586* | 0.403600083 | -1.309001625 | 0.000204878 | Down | Hydroxyproline-rich glycoprotein family protein |
| *Zm00001d016708* | 0.456122432 | -1.132506972 | 6.78E-06 | Down | Beta-fructofuranosidase cell wall isozyme |
| *Zm00001d016719* | 0.430688334 | -1.21528385 | 0.00099609 | Down | U-box domain-containing protein 29 |
| *Zm00001d016727* | 0.495833986 | -1.012070933 | 0.03394626 | Down | Vacuole membrane protein KMS1 |
| *Zm00001d016764* | 0.418955325 | -1.255131683 | 0.000235949 | Down | Ubiquitin-protein ligase |
| *Zm00001d016802* | 0.44469182 | -1.169122228 | 4.04E-09 | Down | L-ascorbate peroxidase S (chloroplastic/mitochondrial) |
| *Zm00001d016924* | 0.334946171 | -1.577998834 | 3.89E-06 | Down | Ethylene insensitive 3-like 5 protein |
| *Zm00001d017019* | 0.307226323 | -1.702626264 | 4.56E-06 | Down | ATFP4 |
| *Zm00001d017060* | 0.325275996 | -1.620263733 | 7.63E-57 | Down | Hypoxia-responsive family protein |
| *Zm00001d017066* | 0.385595857 | -1.374838543 | 0.001023998 | Down | Triacylglycerol lipase SDP1 |
| *Zm00001d017121* | 0.269335488 | -1.892523761 | 1.92E-62 | Down | Glyceraldehyde-3-phosphate dehydrogenase4 |
| *Zm00001d017147* | 0.454459715 | -1.13777568 | 0.00086005 | Down |  |
| *Zm00001d017152* | 0.192303914 | -2.37853997 | 2.99E-20 | Down | Endochitinase precursor4 |
| *Zm00001d017178* | 0.488263638 | -1.034267754 | 7.22E-12 | Down |  |
| *Zm00001d017592* | 0.301458278 | -1.729969748 | 8.95E-06 | Down | Dehydration-responsive element-binding protein 1A |
| *Zm00001d017615* | 0.439052428 | -1.18753487 | 2.17E-17 | Down | RING/U-box superfamily protein |
| *Zm00001d017648* | 0.315082702 | -1.666197544 | 2.19E-07 | Down | OSJNBa0013K16.16-like protein |
| *Zm00001d017682* | 0.497206179 | -1.008083869 | 3.70E-08 | Down | PLATZ transcription factor family protein |
| *Zm00001d017918* | 0.218538004 | -2.194043906 | 7.28E-15 | Down | Trichome birefringence-like 20 |
| *Zm00001d017991* | 0.28351701 | -1.818492799 | 3.66E-16 | Down |  |
| *Zm00001d017992* | 0.395882821 | -1.336854629 | 0.002194031 | Down | Metalloendoproteinase 1 |
| *Zm00001d018106* | 0.301699402 | -1.72881626 | 4.84E-41 | Down | Protein exordium |
| *Zm00001d018107* | 0.365209314 | -1.453204535 | 6.65E-17 | Down | Protein exordium |
| *Zm00001d018155* | 0.440198491 | -1.183773894 | 6.77E-07 | Down | Galactoside 2-alpha-L-fucosyltransferase |
| *Zm00001d018206* | 0.383705435 | -1.381928893 | 4.88E-06 | Down | Nitrate reductase [NADH] 2 |
| *Zm00001d018393* | 0.484647135 | -1.044993371 | 0.003242732 | Down | Protein binding protein |
| *Zm00001d018414* | 0.080168491 | -3.640820875 | 4.62E-47 | Down | IAA9-auxin-responsive Aux/IAA family member |
| *Zm00001d018428* | 0.496702263 | -1.009546776 | 2.26E-05 | Down | Zinc finger (C3HC4-type RING finger) family protein |
| *Zm00001d018799* | 0.482504876 | -1.051384572 | 1.73E-07 | Down | CBL-interacting serine/threonine-protein kinase 23 |
| *Zm00001d018803* | 0.434588231 | -1.202278989 | 4.80E-06 | Down | Probable inositol transporter 2 |
| *Zm00001d018965* | 0.283586005 | -1.818141758 | 2.08E-16 | Down | Xylanase inhibitor protein 1 |
| *Zm00001d019207* | 0.286045425 | -1.805683825 | 8.61E-40 | Down | NAC domain-containing protein 2 |
| *Zm00001d019399* | 0.470881584 | -1.086563794 | 0.019189981 | Down | Putative lipid-transfer protein DIR1 |
| *Zm00001d019411* | 0.458393554 | -1.125341337 | 0.014467355 | Down | L-type lectin-domain containing receptor kinase V.9 |
| *Zm00001d019725* | 0.460144722 | -1.119840415 | 0.00061162 | Down | Uclacyanin-3 |
| *Zm00001d019925* | 0.22433414 | -2.156278901 | 3.32E-67 | Down |  |
| *Zm00001d020137* | 0.329976856 | -1.599563257 | 6.05E-09 | Down | Probable WRKY transcription factor 30 |
| *Zm00001d020251* | 0.334952944 | -1.577969661 | 2.50E-11 | Down |  |
| *Zm00001d020257* | 0.272288849 | -1.876790191 | 4.30E-08 | Down | Heavy metal transport/detoxification superfamily protein |
| *Zm00001d020378* | 0.260068061 | -1.943038861 | 1.04E-06 | Down | Type IV inositol polyphosphate 5-phosphatase 9 |
| *Zm00001d020492* | 0.187869447 | -2.41219763 | 2.31E-23 | Down | Probable WRKY transcription factor 40 |
| *Zm00001d020638* | 0.364798406 | -1.454828669 | 0.00062953 | Down | Protein gamete expressed 1 |
| *Zm00001d020652* | 0.423505769 | -1.239546474 | 0.004437404 | Down | Plant viral-response family protein-like |
| *Zm00001d020697* | 0.285454869 | -1.808665426 | 1.43E-32 | Down | Histone H3 K4-specific methyltransferase SET7/9 family protein |
| *Zm00001d020703* | 0.410157601 | -1.285749729 | 0.002629578 | Down | G-type lectin S-receptor-like serine/threonine-protein kinase |
| *Zm00001d020717* | 0.424530179 | -1.236060978 | 0.005791846 | Down | Abscisic acid 8'-hydroxylase4 |
| *Zm00001d020780* | 0.38225086 | -1.387408346 | 2.79E-17 | Down | Glutathione transferase23 |
| *Zm00001d020925* | 0.310456277 | -1.687537992 | 4.75E-06 | Down | BCR/ABL-regulated protein |
| *Zm00001d020926* | 0.418138536 | -1.257947087 | 2.76E-17 | Down | AN15 |
| *Zm00001d020932* | 0.467423832 | -1.097196801 | 4.10E-05 | Down | Helix-loop-helix DNA-binding domain containing protein |
| *Zm00001d021285* | 0.479432029 | -1.060601802 | 0.003183466 | Down | Protein SHI related sequence 1 |
| *Zm00001d021303* | 0.414575927 | -1.270291749 | 3.77E-06 | Down | Probable receptor-like protein kinase |
| *Zm00001d021422* | 0.464228429 | -1.107093219 | 4.72E-06 | Down | Histone H4 |
| *Zm00001d021494* | 0.360869749 | -1.470449884 | 2.73E-14 | Down | C2 calcium/lipid-binding plant phosphoribosyl transferase family protein |
| *Zm00001d021579* | 0.361529898 | -1.467813134 | 1.82E-34 | Down | Filament-like plant protein 3 |
| *Zm00001d021627* | 0.490339534 | -1.02814701 | 0.030616111 | Down | Senescence regulator |
| *Zm00001d021665* | 0.383353487 | -1.383252792 | 3.16E-11 | Down | PRAS-rich protein |
| *Zm00001d021667* | 0.489868352 | -1.029534005 | 2.02E-06 | Down | Probable xyloglucan endotransglucosylase/hydrolase protein 8 |
| *Zm00001d021732* | 0.174760627 | -2.516547905 | 2.15E-14 | Down | Cellulose synthase-like protein D3 |
| *Zm00001d021775* | 0.363227036 | -1.461056503 | 6.16E-08 | Down | Sugar carrier protein C |
| *Zm00001d021779* | 0.431120545 | -1.213836778 | 2.87E-16 | Down | F-box protein SKIP27 |
| *Zm00001d021803* | 0.312345322 | -1.678786174 | 6.63E-06 | Down | Putative uncharacterized protein |
| *Zm00001d021816* | 0.486096368 | -1.04068574 | 0.026919552 | Down |  |
| *Zm00001d021839* | 0.468898525 | -1.092652353 | 1.76E-05 | Down | Remorin family protein |
| *Zm00001d021882* | 0.348079237 | -1.522512334 | 5.73E-11 | Down |  |
| *Zm00001d021901* | 0.292844329 | -1.771794138 | 5.35E-49 | Down | Stress-induced protein1 |
| *Zm00001d022044* | 0.366279112 | -1.448984666 | 2.21E-05 | Down | Probable LRR receptor-like serine/threonine-protein kinase |
| *Zm00001d022081* | 0.198655296 | -2.331660842 | 6.45E-13 | Down | Protein COBRA |
| *Zm00001d022084* | 0.177414158 | -2.494806951 | 7.82E-17 | Down | B12D protein |
| *Zm00001d022130* | 0.435712545 | -1.198551443 | 6.06E-05 | Down | DUF1677 family protein |
| *Zm00001d022227* | 0.4330256 | -1.207475777 | 3.11E-15 | Down | Fused leaves1 |
| *Zm00001d022245* | 0.258160458 | -1.953660051 | 3.60E-10 | Down | IQ domain-containing protein IQM2 |
| *Zm00001d022314* | 0.414571741 | -1.270306315 | 3.71E-18 | Down | Probable mediator of RNA polymerase II  Transcription subunit 26b |
| *Zm00001d022327* | 0.381242773 | -1.391218106 | 0.000596084 | Down | Probable carboxylesterase 18 |
| *Zm00001d022416* | 0.328218991 | -1.60726938 | 1.80E-73 | Down | Early response to dehydration 15-like protein |
| *Zm00001d022467* | 0.463231157 | -1.110195803 | 0.004222042 | Down | Anthocyanidin 3-O-glucosyltransferase |
| *Zm00001d022553* | 0.497422273 | -1.007456986 | 0.00016147 | Down |  |
| *Zm00001d022636* | 0.34014306 | -1.555786442 | 2.14E-16 | Down | Major facilitator superfamily protein |
| *Zm00001d023379* | 0.37373548 | -1.419910565 | 4.76E-42 | Down | Pyruvate kinase2 |
| *Zm00001d023385* | 0.324626034 | -1.62314939 | 3.39E-07 | Down | Phytosulfokine4 |
| *Zm00001d023404* | 0.274454037 | -1.865363532 | 7.60E-14 | Down | Probable purple acid phosphatase 20 |
| *Zm00001d023700* | 0.28878434 | -1.791935586 | 9.48E-06 | Down | Expressed protein |
| *Zm00001d024210* | 0.445662874 | -1.165975313 | 0.003569839 | Down | Terpene synthase6 |
| *Zm00001d024432* | 0.394671233 | -1.341276727 | 0.000287022 | Down | Phenolic glucoside malonyltransferase 1 |
| *Zm00001d024725* | 0.485081216 | -1.043701781 | 0.022253441 | Down | Putative MYB DNA-binding domain superfamily protein |
| *Zm00001d024843* | 0.304731765 | -1.714388203 | 1.10E-08 | Down | 1-aminocyclopropane-1-carboxylate oxidase15 |
| *Zm00001d025055* | 0.452847811 | -1.14290181 | 1.16E-10 | Down | Protein phosphatase homolog11 |
| *Zm00001d025360* | 0.242623896 | -2.043206446 | 3.06E-36 | Down | Probable F-box protein At1g60180 |
| *Zm00001d025508* | 0.423098427 | -1.240934773 | 2.56E-22 | Down | Class IV heat shock protein |
| *Zm00001d025533* | 0.40027377 | -1.320941016 | 8.15E-05 | Down | NAD(P)-linked oxidoreductase superfamily protein |
| *Zm00001d025545* | 0.33916472 | -1.559941987 | 0.00013085 | Down | Zeaxanthin epoxidase2 |
| *Zm00001d025916* | 0.357748732 | -1.48298144 | 4.18E-30 | Down | E3 ubiquitin-protein ligase RMA1 |
| *Zm00001d026047* | 0.39542578 | -1.338521165 | 6.72E-21 | Down | PLATZ transcription factor family protein |
| *Zm00001d026282* | 0.418682012 | -1.25607316 | 0.003045533 | Down | Bifunctional inhibitor/lipid-transfer protein/seed storage; 2S albumin superfamily protein |
| *Zm00001d026563* | 0.340713561 | -1.553368723 | 1.93E-05 | Down | Ethylene-responsive transcription factor 7 |
| *Zm00001d026619* | 0.496084057 | -1.011343502 | 8.58E-19 | Down | Pyruvate kinase |
| *Zm00001d026649* | 0.392164213 | -1.350470206 | 1.41E-08 | Down | Opaque endosperm7 |
| *Zm00001d026657* | 0.450263473 | -1.15115865 | 0.004192908 | Down | Probable CCR4-associated factor 1 homolog 11 |
| *Zm00001d026662* | 0.460880223 | -1.117536235 | 9.87E-09 | Down | Harpin inducing protein |
| *Zm00001d027355* | 0.38255557 | -1.386258764 | 3.03E-26 | Down | Rhomboid-like protein 3 |
| *Zm00001d027533* | 0.489024474 | -1.032021425 | 0.027495477 | Down | LysM domain-containing GPI-anchored protein 2 |
| *Zm00001d027708* | 0.423482929 | -1.239624282 | 1.13E-06 | Down | Metal ion binding protein |
| *Zm00001d027740* | 0.462809076 | -1.111510939 | 3.32E-28 | Down | Rab28 protein |
| *Zm00001d027901* | 0.421089449 | -1.247801367 | 0.000104847 | Down | ZIM motif family protein |
| *Zm00001d027924* | 0.294707426 | -1.762644683 | 1.53E-43 | Down | AP2-EREBP transcription factor |
| *Zm00001d027928* | 0.325078739 | -1.62113889 | 1.49E-06 | Down |  |
| *Zm00001d027988* | 0.410375963 | -1.284981865 | 0.000105907 | Down |  |
| *Zm00001d028055* | 0.468530973 | -1.093783671 | 1.27E-13 | Down | Probable E3 ubiquitin-protein ligase RHY1A |
| *Zm00001d028241* | 0.489988075 | -1.029181456 | 0.001387094 | Down | 3-ketoacyl-CoA synthase |
| *Zm00001d028307* | 0.19828544 | -2.334349349 | 4.92E-15 | Down | Probable inactive poly [ADP-ribose] polymerase SRO1 |
| *Zm00001d028404* | 0.495037099 | -1.014391446 | 1.56E-09 | Down | Chaperone protein dnaJ 11 (chloroplastic) |
| *Zm00001d028561* | 0.384736295 | -1.378058158 | 1.76E-33 | Down | 17.4 kDa class I heat shock protein |
| *Zm00001d028577* | 0.422196479 | -1.244013549 | 1.69E-19 | Down | DNA-binding protein |
| *Zm00001d028585* | 0.441135878 | -1.180704995 | 0.009082954 | Down | Sigma-like factor2b |
| *Zm00001d028598* | 0.466830116 | -1.099030461 | 0.000435889 | Down | ROTUNDIFOLIA like 8 |
| *Zm00001d028630* | 0.304684595 | -1.714611534 | 1.25E-51 | Down | Heat shock cognate 70 kDa protein 2 |
| *Zm00001d028718* | 0.326577282 | -1.614503662 | 2.41E-19 | Down | Hydrophobic protein RCI2B |
| *Zm00001d028725* | 0.430097756 | -1.217263492 | 1.29E-05 | Down | Beta3-glucuronyltransferase |
| *Zm00001d028806* | 0.439218946 | -1.186987806 | 0.004104866 | Down | Sex determination protein tassel seed-2 |
| *Zm00001d028809* | 0.45789398 | -1.126914497 | 0.013655591 | Down | PLAC8 family protein |
| *Zm00001d028930* | 0.191254426 | -2.38643496 | 3.12E-34 | Down | MYB domain protein 112 |
| *Zm00001d028931* | 0.474989671 | -1.074031953 | 0.013553163 | Down | Galactinol synthase1 |
| *Zm00001d028968* | 0.483236131 | -1.049199766 | 3.94E-14 | Down | Chaperone protein dnaJ 20 (chloroplastic) |
| *Zm00001d028987* | 0.497063715 | -1.008497304 | 0.003888815 | Down | Phospholipid-transporting ATPase 1 |
| *Zm00001d029011* | 0.481483313 | -1.054442297 | 3.42E-14 | Down | Endoglucanase 25 |
| *Zm00001d029098* | 0.476823781 | -1.068471905 | 0.007994182 | Down | Sugars will eventually be exported transporter16 |
| *Zm00001d029102* | 0.473083933 | -1.079831932 | 1.41E-13 | Down | Dormancy-associated protein homolog 3 |
| *Zm00001d029313* | 0.414519063 | -1.270489646 | 0.000285804 | Down | Plasmodesmata callose-binding protein 5 |
| *Zm00001d029359* | 0.454577713 | -1.137401142 | 0.001309774 | Down | Benzoxazinone synthesis10 |
| *Zm00001d029630* | 0.241127887 | -2.05212958 | 2.88E-16 | Down | Cystatin6 |
| *Zm00001d029841* | 0.479304959 | -1.060984229 | 0.000594105 | Down | Myosin heavy chain-related |
| *Zm00001d029853* | 0.39160225 | -1.35253904 | 0.000181471 | Down | Proline oxidase |
| *Zm00001d029934* | 0.481858572 | -1.053318326 | 0.003177725 | Down | Homeobox-leucine zipper protein HAT4 |
| *Zm00001d030158* | 0.492238461 | -1.022570707 | 1.41E-11 | Down | Protein NRT1/ PTR FAMILY 8.3 |
| *Zm00001d030171* | 0.466333702 | -1.100565398 | 0.001454498 | Down | Serine/threonine-protein kinase |
| *Zm00001d030993* | 0.437302114 | -1.193297771 | 0.004758198 | Down | Auxin-responsive protein IAA4 |
| *Zm00001d031222* | 0.47915952 | -1.061422062 | 0.001716953 | Down | Cell Division Protein AAA ATPase family |
| *Zm00001d031228* | 0.367975389 | -1.442318818 | 7.69E-41 | Down |  |
| *Zm00001d031264* | 0.186716698 | -2.421077138 | 4.76E-44 | Down | Cysteine dioxygenase |
| *Zm00001d031278* | 0.282920636 | -1.821530686 | 5.03E-07 | Down | Dof zinc finger protein DOF1.6 |
| *Zm00001d031315* | 0.473515275 | -1.078517128 | 6.69E-30 | Down | Transducin/WD40 repeat-like superfamily protein |
| *Zm00001d031375* | 0.269025487 | -1.894185236 | 2.99E-06 | Down | Calcium-binding EF hand family protein |
| *Zm00001d031396* | 0.320879039 | -1.639898543 | 7.99E-05 | Down | Calcium-binding EF hand family protein |
| *Zm00001d031404* | 0.494794072 | -1.015099878 | 0.012756153 | Down | Calcium-binding EF hand family protein |
| *Zm00001d031458* | 0.385265478 | -1.376075176 | 0.000177139 | Down | Hat transposon superfamily protein |
| *Zm00001d031601* | 0.356275186 | -1.488936088 | 0.000193022 | Down | Receptor-like kinase-like |
| *Zm00001d031677* | 0.273657032 | -1.869559164 | 6.19E-56 | Down | Mtn19-like protein |
| *Zm00001d031796* | 0.411541025 | -1.280891841 | 0.000281531 | Down | AP2 domain containing protein |
| *Zm00001d032091* | 0.361723817 | -1.467039504 | 1.44E-10 | Down | SAUR33-auxin-responsive SAUR family member |
| *Zm00001d032265* | 0.22222439 | -2.169910926 | 7.92E-12 | Down | WRKY69-superfamily of TFs having WRKY and zinc finger domains |
| *Zm00001d032298* | 0.340325779 | -1.555011657 | 3.41E-16 | Down | Trehalose-6-phosphate phosphatase1 |
| *Zm00001d032310* | 0.441186124 | -1.180540678 | 0.005679461 | Down | Protein kinase superfamily protein |
| *Zm00001d032386* | 0.461177183 | -1.116606957 | 1.72E-19 | Down | Phosphofructose kinase2 |
| *Zm00001d032438* | 0.341412699 | -1.550411373 | 2.19E-05 | Down | AAA-ATPase |
| *Zm00001d032506* | 0.429873586 | -1.218015629 | 0.005260843 | Down |  |
| *Zm00001d032552* | 0.313483901 | -1.67353674 | 7.34E-42 | Down | Protein neoxanthin-deficient 1 |
| *Zm00001d032740* | 0.417280314 | -1.260911234 | 0.000137508 | Down | Protein NRT1/ PTR FAMILY 5.2 |
| *Zm00001d032849* | 0.449703974 | -1.152952463 | 4.39E-17 | Down | RING/U-box superfamily protein |
| *Zm00001d032850* | 0.196973759 | -2.343924652 | 5.26E-36 | Down | Phosphate transporter protein2 |
| *Zm00001d032866* | 0.378051793 | -1.403344196 | 9.91E-06 | Down | UDP-glycosyltransferase 86A2 |
| *Zm00001d032873* | 0.4684236 | -1.094114333 | 5.66E-25 | Down | Metal ion binding protein |
| *Zm00001d033049* | 0.271097782 | -1.883114784 | 3.39E-08 | Down | ZIM motif family protein |
| *Zm00001d033050* | 0.408130651 | -1.292897033 | 0.00225313 | Down | ZIM motif family protein |
| *Zm00001d033091* | 0.465381055 | -1.103515613 | 5.77E-11 | Down | Mpv17 / PMP22 family protein |
| *Zm00001d033316* | 0.467094273 | -1.09821434 | 5.99E-15 | Down | CBL-interacting serine/threonine-protein kinase 4 |
| *Zm00001d033385* | 0.479692167 | -1.059819214 | 0.024300872 | Down | Probable galacturonosyltransferase-like 1 |
| *Zm00001d033489* | 0.473951835 | -1.077187641 | 0.021404814 | Down | Plant cysteine oxidase 2 |
| *Zm00001d033705* | 0.48165882 | -1.05391651 | 3.95E-12 | Down | Myosin-binding protein 3 |
| *Zm00001d033839* | 0.474979232 | -1.074063659 | 7.06E-05 | Down | Probable LRR receptor-like serine/threonine-protein kinase |
| *Zm00001d033862* | 0.453162833 | -1.141898554 | 0.012844054 | Down | 1-aminocyclopropane-1-carboxylate synthase6 |
| *Zm00001d033906* | 0.383128226 | -1.384100778 | 2.46E-27 | Down | Endoplasmic oxidoreductin-1 |
| *Zm00001d033924* | 0.319951473 | -1.644074988 | 5.90E-05 | Down |  |
| *Zm00001d034037* | 0.268359081 | -1.897763384 | 4.25E-100 | Down | Circumsporozoite protein |
| *Zm00001d034040* | 0.49624543 | -1.010874278 | 0.032048545 | Down | Probable calcium-binding protein CML27 |
| *Zm00001d034107* | 0.410952824 | -1.282955308 | 0.003788521 | Down | Cytochrome P450 family 81 subfamily D polypeptide 8 |
| *Zm00001d034173* | 0.476353198 | -1.069896421 | 0.000679413 | Down | Nuclear pore complex protein NUP58 |
| *Zm00001d034175* | 0.299735304 | -1.738239073 | 4.47E-07 | Down | CCG-binding protein 1 |
| *Zm00001d034197* | 0.24867434 | -2.00767045 | 7.97E-14 | Down |  |
| *Zm00001d034356* | 0.385654608 | -1.374618747 | 4.36E-21 | Down | Glutathione transferase5 |
| *Zm00001d034388* | 0.463687141 | -1.108776377 | 1.57E-29 | Down | Aldehyde oxidase4 |
| *Zm00001d034558* | 0.334460037 | -1.580094255 | 3.13E-14 | Down | Remorin |
| *Zm00001d034588* | 0.339724459 | -1.557563005 | 3.79E-21 | Down | Protein CDI |
| *Zm00001d034615* | 0.363782115 | -1.458853481 | 4.53E-13 | Down | Protein LURP-one-related 14 |
| *Zm00001d034642* | 0.379246217 | -1.398793304 | 0.001233063 | Down | Zinc finger protein ZAT11 |
| *Zm00001d035115* | 0.21780447 | -2.198894533 | 1.19E-20 | Down | Multidrug and toxic compound extrusion1 |
| *Zm00001d035178* | 0.225986635 | -2.145690644 | 2.11E-18 | Down | Cytochrome P450 71A26 |
| *Zm00001d035236* | 0.446428014 | -1.163500534 | 0.008954054 | Down | Plant calmodulin-binding protein-related |
| *Zm00001d035440* | 0.443859124 | -1.17182624 | 2.11E-16 | Down | Low temperature-induced protein15 |
| *Zm00001d035753* | 0.407179833 | -1.296261987 | 0.002856636 | Down | Polygalacturonase inhibitor |
| *Zm00001d035916* | 0.24476518 | -2.030529755 | 9.43E-10 | Down | UMP/CMP kinase1 |
| *Zm00001d036003* | 0.280368138 | -1.834605689 | 1.08E-13 | Down | DRE-binding protein4 |
| *Zm00001d036197* | 0.48100361 | -1.055880374 | 0.000336898 | Down | HXXXD-type acyl-transferase family protein |
| *Zm00001d036409* | 0.493643615 | -1.018458227 | 4.17E-13 | Down | Osjnba0013K16.15-like protein |
| *Zm00001d036450* | 0.320433462 | -1.641903281 | 9.78E-07 | Down | F-box domain containing protein |
| *Zm00001d036537* | 0.315113864 | -1.666054865 | 1.21E-06 | Down | Pyrophosphate--fructose 6-phosphate 1-phosphotransferase; subunit alpha 2 |
| *Zm00001d036551* | 0.474923722 | -1.074232275 | 0.002837835 | Down | Transcription factor MYB104 |
| *Zm00001d036671* | 0.370859203 | -1.431056524 | 1.02E-07 | Down |  |
| *Zm00001d036700* | 0.407293756 | -1.295858398 | 9.71E-08 | Down | CASP-like protein 13 |
| *Zm00001d036797* | 0.497398325 | -1.007526446 | 4.76E-08 | Down | Non-structural maintenance of chromosomes element 4 homolog A |
| *Zm00001d036902* | 0.321130488 | -1.638768453 | 3.31E-05 | Down |  |
| *Zm00001d036925* | 0.402489278 | -1.312977743 | 7.56E-32 | Down | Protein kinase Kelch repeat:Kelch |
| *Zm00001d037150* | 0.259116877 | -1.94832511 | 6.00E-49 | Down | Probable tyrosine-protein phosphatase |
| *Zm00001d037165* | 0.496379697 | -1.010483987 | 0.03172987 | Down | Putative AP2/EREBP transcription factor superfamily protein |
| *Zm00001d037205* | 0.492999475 | -1.020341984 | 0.009061572 | Down | Protein EXORDIUM |
| *Zm00001d037395* | 0.418306363 | -1.257368151 | 0.004798733 | Down | Polyadenylate-binding protein-interacting protein 10 |
| *Zm00001d037656* | 0.258568768 | -1.95138007 | 8.36E-102 | Down | Xylanase inhibitor protein 1 |
| *Zm00001d037689* | 0.388650403 | -1.363455083 | 1.69E-51 | Down | Hexokinase7 |
| *Zm00001d037693* | 0.352403908 | -1.504698172 | 8.98E-08 | Down | Malic enzyme |
| *Zm00001d037724* | 0.411948737 | -1.279463277 | 1.23E-31 | Down | Gibberellin 2-beta-dioxygenase |
| *Zm00001d037747* | 0.390232335 | -1.357594768 | 0.001273917 | Down | Prenylated Rab acceptor family protein |
| *Zm00001d037783* | 0.316819086 | -1.658268846 | 2.24E-11 | Down | Putative receptor protein kinase zmpk1 |
| *Zm00001d038003* | 0.474755116 | -1.074744549 | 2.07E-32 | Down |  |
| *Zm00001d038060* | 0.345578579 | -1.532914301 | 4.36E-07 | Down | Probable microtubule-binding protein TANGLED |
| *Zm00001d038151* | 0.240631165 | -2.055104594 | 4.27E-21 | Down |  |
| *Zm00001d038165* | 0.378230014 | -1.402664244 | 4.84E-42 | Down | Putative GID1-like gibberellin receptor |
| *Zm00001d038280* | 0.466853531 | -1.0989581 | 0.004892955 | Down | U-box domain-containing protein 29 |
| *Zm00001d038291* | 0.401803045 | -1.315439596 | 1.35E-09 | Down | Ferredoxin |
| *Zm00001d038320* | 0.288540606 | -1.793153733 | 1.89E-10 | Down | Putative AP2/EREBP transcription factor superfamily protein |
| *Zm00001d038471* | 0.487027777 | -1.037924039 | 0.011215958 | Down | U-box domain-containing protein 16 |
| *Zm00001d038476* | 0.442305386 | -1.176885286 | 0.000527782 | Down | Alpha expansin5 |
| *Zm00001d038526* | 0.451518624 | -1.147142597 | 4.71E-05 | Down |  |
| *Zm00001d038775* | 0.4546345 | -1.137220929 | 1.69E-20 | Down | ATP-dependent 6-phosphofructokinase 3 |
| *Zm00001d038780* | 0.493135571 | -1.019943773 | 7.69E-10 | Down | Oxidative stress 3 |
| *Zm00001d038791* | 0.473899199 | -1.077347872 | 6.63E-06 | Down | Putative leucine-rich repeat receptor-like protein kinase family protein |
| *Zm00001d038810* | 0.48712926 | -1.037623453 | 0.028094774 | Down | DNA-binding WRKY |
| *Zm00001d038860* | 0.486818036 | -1.038545477 | 0.003276757 | Down | MHM17_10-like protein |
| *Zm00001d038959* | 0.490764544 | -1.026897072 | 0.002446009 | Down | O-acyltransferase WSD1 |
| *Zm00001d039001* | 0.447950087 | -1.158590107 | 2.93E-19 | Down | Expressed protein |
| *Zm00001d039532* | 0.266237229 | -1.909215771 | 4.21E-07 | Down | Probable WRKY transcription factor 36 |
| *Zm00001d039607* | 0.114265238 | -3.129541528 | 6.85E-17 | Down | UDP-glycosyltransferase 75B1 |
| *Zm00001d039642* | 0.291561578 | -1.778127481 | 1.51E-05 | Down | UDP-glycosyltransferase 73D1 |
| *Zm00001d039771* | 0.26526401 | -1.914499144 | 2.63E-47 | Down |  |
| *Zm00001d039826* | 0.336974383 | -1.569289172 | 3.61E-18 | Down |  |
| *Zm00001d039935* | 0.286969113 | -1.801032629 | 4.34E-14 | Down | Heat shock protein17.2 |
| *Zm00001d039936* | 0.351738959 | -1.507422955 | 7.00E-45 | Down | 17.4 kDa class I heat shock protein |
| *Zm00001d039993* | 0.421500751 | -1.246392893 | 0.004869692 | Down | Putative uncharacterized protein |
| *Zm00001d040002* | 0.443860606 | -1.171821424 | 4.48E-07 | Down | Metal tolerance protein 10 |
| *Zm00001d040291* | 0.486908904 | -1.03827621 | 1.13E-07 | Down | Dynein light chain type 1 family protein |
| *Zm00001d040292* | 0.244411763 | -2.032614372 | 1.32E-43 | Down | Protein induced upon tuberization |
| *Zm00001d040446* | 0.307159134 | -1.702941809 | 2.89E-30 | Down | Pyruvate kinase |
| *Zm00001d040682* | 0.410032033 | -1.286191471 | 2.99E-07 | Down | Sequence-specific DNA binding transcription factors |
| *Zm00001d041826* | 0.474073432 | -1.07681755 | 0.005727696 | Down | Protein kinase domain containing protein |
| *Zm00001d042056* | 0.471053601 | -1.086036862 | 0.016450363 | Down | Calcium-binding protein CML38 |
| *Zm00001d042063* | 0.275946874 | -1.857537554 | 1.15E-14 | Down |  |
| *Zm00001d042114* | 0.244197876 | -2.033877441 | 3.59E-63 | Down |  |
| *Zm00001d042140* | 0.351961718 | -1.506509575 | 3.56E-31 | Down | Beta-1,3-glucanase |
| *Zm00001d042211* | 0.491997437 | -1.023277296 | 2.41E-08 | Down | PsbP domain-containing protein 7 (chloroplastic) |
| *Zm00001d042246* | 0.363674832 | -1.459279008 | 2.70E-16 | Down |  |
| *Zm00001d042285* | 0.370432326 | -1.432718092 | 5.81E-10 | Down |  |
| *Zm00001d042349* | 0.466930462 | -1.098720384 | 0.009875426 | Down | Uncharacterized protein |
| *Zm00001d042353* | 0.269401947 | -1.892167817 | 7.28E-12 | Down | Sucrose phosphate synthase2 |
| *Zm00001d042388* | 0.398418744 | -1.327642571 | 6.81E-07 | Down | DUF538 family protein |
| *Zm00001d042411* | 0.374960553 | -1.415189268 | 0.000856569 | Down | Protein DETOXIFICATION 42 |
| *Zm00001d042446* | 0.458417322 | -1.125266534 | 0.013772221 | Down | KDEL-tailed cysteine endopeptidase CEP1 |
| *Zm00001d042449* | 0.379040413 | -1.39957642 | 3.80E-05 | Down | BCL-2 binding anthanogene-1 |
| *Zm00001d042572* | 0.30063127 | -1.733933016 | 5.11E-07 | Down |  |
| *Zm00001d042739* | 0.365182069 | -1.453312164 | 0.000238082 | Down | Anthocyanidin 3-O-glucosyltransferase |
| *Zm00001d042779* | 0.477859037 | -1.065342994 | 2.12E-12 | Down | Protein ABSCISIC ACID-INSENSITIVE 5 |
| *Zm00001d042810* | 0.476509676 | -1.069422586 | 0.01954847 | Down | Cell division control protein 6 homolog B |
| *Zm00001d042887* | 0.497872864 | -1.006150709 | 0.009624614 | Down | Avr9/Cf-9 rapidly elicited protein 137 |
| *Zm00001d042898* | 0.398716805 | -1.326563683 | 1.99E-19 | Down | Aspartic proteinase PCS1 |
| *Zm00001d042944* | 0.436554298 | -1.195766989 | 0.006313284 | Down | Alpha/beta-Hydrolases superfamily protein |
| *Zm00001d042975* | 0.46939812 | -1.09111603 | 0.000504149 | Down | Serine carboxypeptidase-like 27 |
| *Zm00001d043025* | 0.461305783 | -1.116204715 | 0.015603164 | Down | Probable WRKY transcription factor 33 |
| *Zm00001d043043* | 0.488906219 | -1.032370339 | 6.65E-14 | Down | U-box domain-containing protein 16 |
| *Zm00001d043097* | 0.48017863 | -1.058356896 | 5.27E-06 | Down | Ribosomal protein L34e superfamily protein |
| *Zm00001d043104* | 0.462503973 | -1.112462335 | 1.64E-34 | Down |  |
| *Zm00001d043131* | 0.462484896 | -1.112521843 | 9.97E-08 | Down | MYB domain protein 81 |
| *Zm00001d043205* | 0.338148444 | -1.564271379 | 2.68E-09 | Down | Ethylene-responsive transcription factor 4 |
| *Zm00001d043512* | 0.389059269 | -1.361938145 | 1.08E-09 | Down | Protein kinase family protein with leucine-rich repeat domain |
| *Zm00001d043525* | 0.386988352 | -1.369637953 | 5.40E-32 | Down | Oxidative stress 3 |
| *Zm00001d043574* | 0.103079063 | -3.278176767 | 8.33E-81 | Down |  |
| *Zm00001d043639* | 0.481009287 | -1.055863347 | 0.005443156 | Down |  |
| *Zm00001d043723* | 0.41070656 | -1.283820106 | 1.37E-09 | Down | F-box/kelch-repeat protein |
| *Zm00001d043740* | 0.463463648 | -1.10947191 | 0.011913372 | Down | Mitogen-activated protein kinase kinase kinase YODA |
| *Zm00001d043782* | 0.442904144 | -1.1749336 | 1.47E-20 | Down | DNA-binding protein RAV1 |
| *Zm00001d043795* | 0.474315462 | -1.076081196 | 0.006501002 | Down | Glutathione S-transferase GSTU6 |
| *Zm00001d043800* | 0.368636839 | -1.439727843 | 2.11E-16 | Down | Leaf rust 10 disease-resistance locus; Receptor-like protein kinase-like 1.1 |
| *Zm00001d043839* | 0.408383565 | -1.292003289 | 1.01E-05 | Down |  |
| *Zm00001d043853* | 0.346638139 | -1.5284977 | 3.33E-16 | Down | O-acyltransferase WSD1 |
| *Zm00001d043950* | 0.456233603 | -1.132155385 | 0.000289708 | Down | Probable WRKY transcription factor 71 |
| *Zm00001d043974* | 0.474532305 | -1.07542179 | 0.00228047 | Down | DUF1645 family protein |
| *Zm00001d043994* | 0.456293453 | -1.131966142 | 0.003341457 | Down | Aldehyde oxygenase (deformylating) |
| *Zm00001d043998* | 0.446131138 | -1.164460249 | 6.47E-29 | Down | Ubiquitin-conjugating enzyme E2 8 |
| *Zm00001d044253* | 0.413556877 | -1.273842336 | 2.85E-13 | Down | IQ domain-containing protein IQM5 |
| *Zm00001d044291* | 0.278853497 | -1.842420733 | 2.21E-25 | Down | Actin binding protein family |
| *Zm00001d044426* | 0.476593054 | -1.069170171 | 2.51E-28 | Down | DnaJ-like protein |
| *Zm00001d044688* | 0.489902618 | -1.029433093 | 0.030221239 | Down |  |
| *Zm00001d044734* | 0.446227304 | -1.164149301 | 0.005455108 | Down | U-box domain-containing protein 21 |
| *Zm00001d044754* | 0.368195855 | -1.44145471 | 4.00E-09 | Down | Pyrophosphate--fructose 6-phosphate 1-phosphotransferase subunit beta 2 |
| *Zm00001d044833* | 0.326089625 | -1.616659554 | 3.45E-07 | Down | Protein SUR2 |
| *Zm00001d044841* | 0.447690426 | -1.15942663 | 2.32E-09 | Down | Glutaredoxin family protein |
| *Zm00001d044995* | 0.434814508 | -1.201528017 | 1.58E-05 | Down | Root-specific kinase 1 |
| *Zm00001d045051* | 0.316947985 | -1.657681997 | 9.63E-06 | Down | Zinc finger nuclease2 |
| *Zm00001d045063* | 0.328279813 | -1.607002057 | 4.89E-05 | Down | Cytochrome P450 709B2 |
| *Zm00001d045361* | 0.253422122 | -1.980385628 | 8.31E-08 | Down |  |
| *Zm00001d045370* | 0.431342163 | -1.21309535 | 2.09E-26 | Down |  |
| *Zm00001d045432* | 0.282565883 | -1.823340809 | 1.67E-10 | Down |  |
| *Zm00001d045475* | 0.470650457 | -1.087272099 | 1.78E-15 | Down | Histone H3 |
| *Zm00001d045695* | 0.402110388 | -1.314336488 | 0.00012214 | Down | Aspartyl protease APCB1 |
| *Zm00001d045785* | 0.427994161 | -1.224336979 | 0.000936293 | Down | Leucine-rich repeat receptor-like serine/threonine/tyrosine-protein kinase SOBIR1 |
| *Zm00001d046145* | 0.403184034 | -1.310489584 | 2.26E-10 | Down | Putative RING zinc finger domain superfamily protein |
| *Zm00001d046184* | 0.425997474 | -1.231083219 | 6.88E-06 | Down | Peroxidase 52 |
| *Zm00001d046234* | 0.462900563 | -1.111225778 | 3.28E-06 | Down | Inositol oxygenase 2 |
| *Zm00001d046263* | 0.473137207 | -1.079669477 | 1.26E-06 | Down | Amino acid permease 6 |
| *Zm00001d046472* | 0.360715684 | -1.471065941 | 4.96E-09 | Down | Hemoglobin-like protein HbO |
| *Zm00001d046475* | 0.497054496 | -1.008524061 | 0.021826008 | Down |  |
| *Zm00001d046632* | 0.472728058 | -1.080917598 | 3.32E-19 | Down | MYB transcription factor |
| *Zm00001d046865* | 0.303722983 | -1.71917201 | 2.13E-29 | Down | Protein eceriferum 3 |
| *Zm00001d046949* | 0.404621935 | -1.305353561 | 5.68E-17 | Down | Haloacid dehalogenase-like hydrolase (HAD) superfamily protein |
| *Zm00001d046961* | 0.443412029 | -1.173280185 | 0.006332204 | Down | VQ motif family protein |
| *Zm00001d047124* | 0.144159973 | -2.794257454 | 2.90E-28 | Down | Proline oxidase |
| *Zm00001d047402* | 0.299052254 | -1.741530503 | 2.87E-36 | Down | Putative calmodulin-binding family protein |
| *Zm00001d047582* | 0.411674597 | -1.280423666 | 1.73E-35 | Down | Galactinol synthase3 |
| *Zm00001d047661* | 0.363206208 | -1.461139234 | 0.000121371 | Down | Tetratricopeptide repeat (TPR)-like superfamily protein |
| *Zm00001d047664* | 0.20956026 | -2.254562938 | 9.76E-13 | Down | Multidrug resistance protein ABC transporter family protein |
| *Zm00001d047705* | 0.349520277 | -1.516551941 | 0.000127567 | Down | Cyclase/dehydrase family protein |
| *Zm00001d047758* | 0.351049689 | -1.510252845 | 1.35E-18 | Down | MAP kinase1 |
| *Zm00001d047808* | 0.417206481 | -1.261166526 | 5.39E-21 | Down | Initiator binding protein1 |
| *Zm00001d047820* | 0.487231003 | -1.03732216 | 0.00563293 | Down | Rotundifolia like 8 |
| *Zm00001d047841* | 0.395842655 | -1.337001011 | 1.01E-33 | Down | 17.4 kDa class I heat shock protein |
| *Zm00001d047917* | 0.451987746 | -1.145644434 | 0.000309859 | Down | ACT domain-containing protein ACR8 |
| *Zm00001d047931* | 0.264217946 | -1.920199633 | 7.89E-12 | Down | 3-ketoacyl-coa synthase |
| *Zm00001d047937* | 0.491285643 | -1.025366017 | 2.69E-06 | Down | Armadillo repeat only 1 |
| *Zm00001d048020* | 0.154061128 | -2.698425203 | 1.14E-18 | Down | Hemoglobin1 |
| *Zm00001d048044* | 0.495023223 | -1.014431888 | 0.027225174 | Down | Putative NAC domain transcription factor superfamily protein |
| *Zm00001d048061* | 0.162057821 | -2.625419447 | 1.29E-25 | Down | 3-ketoacyl-coa synthase |
| *Zm00001d048348* | 0.426435139 | -1.22960177 | 5.52E-23 | Down | Transcription activator-related |
| *Zm00001d048533* | 0.482562535 | -1.051212183 | 0.013271604 | Down | Cyclin-dependent protein kinase inhibitor EL2 |
| *Zm00001d048948* | 0.460169466 | -1.119762835 | 4.85E-07 | Down | Hevein-like preproprotein |
| *Zm00001d049081* | 0.487916492 | -1.035293848 | 0.023839121 | Down |  |
| *Zm00001d049103* | 0.378669376 | -1.400989343 | 0.000352624 | Down | Cysteine/Histidine-rich C1 domain family protein |
| *Zm00001d049157* | 0.298330143 | -1.745018341 | 4.86E-11 | Down | Octicosapeptide/Phox/Bem1p (PB1) domain-containing protein |
| *Zm00001d049510* | 0.452464105 | -1.144124752 | 1.35E-20 | Down | Putative cellulose synthase-like family protein |
| *Zm00001d049630* | 0.431526807 | -1.212477912 | 9.04E-11 | Down | Galactose oxidase/kelch repeat superfamily protein |
| *Zm00001d049860* | 0.381253627 | -1.391177031 | 2.39E-15 | Down | NAC domain containing protein 84 |
| *Zm00001d049995* | 0.282930232 | -1.821481754 | 4.16E-10 | Down | Nitrate reductase |
| *Zm00001d050093* | 0.21762197 | -2.200103885 | 4.16E-18 | Down | SAUR33-auxin-responsive SAUR family member |
| *Zm00001d050164* | 0.464956758 | -1.104831547 | 0.005835501 | Down | Wall-associated receptor kinase-like 20 |
| *Zm00001d050557* | 0.335597672 | -1.575195389 | 4.74E-11 | Down | Chaperone protein dnaJ-related |
| *Zm00001d050899* | 0.269465337 | -1.891828395 | 2.22E-11 | Down | Cell number regulator 2 |
| *Zm00001d051001* | 0.419748538 | -1.252402794 | 0.001740778 | Down | Glyceraldehyde-3-phosphate dehydrogenase |
| *Zm00001d051156* | 0.482238729 | -1.052180575 | 0.000686389 | Down | Putative phosphoenolpyruvate carboxylase kinase family protein |
| *Zm00001d051468* | 0.320371913 | -1.642180421 | 6.17E-06 | Down | RING/U-box superfamily protein |
| *Zm00001d051525* | 0.430830816 | -1.214806651 | 0.000219478 | Down | Oligopeptide transporter 4 |
| *Zm00001d051554* | 0.314076278 | -1.670813114 | 5.10E-16 | Down | Abscisic acid 8'-hydroxylase2 |
| *Zm00001d051938* | 0.122805453 | -3.025553466 | 9.30E-169 | Down | Cinnamoyl-CoA reductase 1 |
| *Zm00001d052162* | 0.359666664 | -1.475267645 | 7.62E-15 | Down |  |
| *Zm00001d052206* | 0.487591343 | -1.036255585 | 0.003660724 | Down | Protein exordium |
| *Zm00001d052208* | 0.264561283 | -1.918326146 | 7.26E-11 | Down | Epidermal patterning factor-like protein 1 |
| *Zm00001d052209* | 0.481163223 | -1.055401719 | 0.02512558 | Down | Cytokinin-O-glucosyltransferase 2 |
| *Zm00001d052333* | 0.464570995 | -1.106029012 | 2.69E-16 | Down | Hypoxia-responsive family protein |
| *Zm00001d052372* | 0.355755619 | -1.491041552 | 1.05E-05 | Down | Protein LURP-one-related 8 |
| *Zm00001d052494* | 0.454269657 | -1.138379151 | 1.01E-15 | Down | Pyruvate kinase |
| *Zm00001d052653* | 0.365216606 | -1.453175729 | 6.33E-25 | Down | Respiratory burst oxidase4 |
| *Zm00001d052661* | 0.276351725 | -1.855422477 | 2.06E-10 | Down |  |
| *Zm00001d053087* | 0.371011142 | -1.430465583 | 1.10E-06 | Down | G-type lectin S-receptor-like serine/threonine-protein kinase SD2-5 |
| *Zm00001d053212* | 0.418764472 | -1.255789045 | 2.17E-14 | Down | E3 ubiquitin ligase BIG BROTHER-related |
| *Zm00001d053378* | 0.459246719 | -1.122658681 | 2.93E-15 | Down |  |
| *Zm00001d053626* | 0.379831743 | -1.396567615 | 8.62E-17 | Down | SPX domain-containing protein 1 |
| *Zm00001d053715* | 0.207369248 | -2.269726133 | 9.29E-48 | Down | UDP-glycosyltransferase 84A1 |
| *Zm00001d053746* | 0.393842741 | -1.344308411 | 0.002122426 | Down | Probable WRKY transcription factor 40 |
| *Zm00001d053988* | 0.416472972 | -1.263705224 | 1.89E-16 | Down | BZIP transcription factor |
| *Zm00001d053995* | 0.39593414 | -1.336667624 | 3.80E-07 | Down |  |
| *Zm00001d054044* | 0.401451501 | -1.316702386 | 0.000124429 | Down | Catalase isozyme 3 |
| *Zm00001d000204* | 2.614611117 | 1.386596384 | 1.14E-08 | Up | Probable sulfate transporter 3.4 |
| *Zm00001d000299* | 2.076538258 | 1.054180452 | 0.016476807 | Up | Endosomal targeting BRO1-like domain-containing protein |
| *Zm00001d001989* | 4.063835141 | 2.022841877 | 3.26E-08 | Up |  |
| *Zm00001d002000* | 3.957034376 | 1.984419598 | 4.66E-07 | Up | Lipoxygenase6 |
| *Zm00001d002058* | 2.227760289 | 1.155594005 | 4.85E-05 | Up |  |
| *Zm00001d002284* | 2.053149563 | 1.037838725 | 1.32E-05 | Up | F-box/kelch-repeat protein |
| *Zm00001d002346* | 2.065558346 | 1.046531813 | 0.010848352 | Up | Cinnamyl alcohol dehydrogenase1 |
| *Zm00001d002347* | 2.820307676 | 1.495852559 | 0.000131557 | Up | Bifunctional inhibitor/lipid-transfer protein/seed storage  2S albumin superfamily protein |
| *Zm00001d002503* | 2.666879476 | 1.415152627 | 6.23E-08 | Up | ABC transporter C family member 9 |
| *Zm00001d002760* | 2.023836294 | 1.017092597 | 0.027505508 | Up | Ethylene-responsive transcription factor ERF105 |
| *Zm00001d003379* | 2.226519532 | 1.154790268 | 0.002240805 | Up | Major latex protein 22 |
| *Zm00001d003559* | 2.026397866 | 1.018917463 | 0.030780157 | Up | Ferric reduction oxidase 7 (chloroplastic) |
| *Zm00001d003567* | 3.696645765 | 1.886216803 | 3.64E-06 | Up | 26S proteasome non-ATPase regulatory subunit 11 homolog |
| *Zm00001d003823* | 2.071289455 | 1.050529179 | 4.71E-05 | Up | Cytochrome P450 77A4 |
| *Zm00001d003848* | 2.266811976 | 1.180664729 | 6.07E-06 | Up |  |
| *Zm00001d003974* | 2.103256456 | 1.072624773 | 1.90E-08 | Up | Phosphoenolpyruvate carboxylase family protein |
| *Zm00001d004416* | 2.009734492 | 1.007004918 | 2.24E-05 | Up | Probable RNA-dependent RNA polymerase 5 |
| *Zm00001d004635* | 2.734614329 | 1.45133738 | 0.000354911 | Up | Leucine-rich repeat protein kinase family protein |
| *Zm00001d004649* | 2.04778812 | 1.03406645 | 0.024804427 | Up |  |
| *Zm00001d005146* | 2.097822839 | 1.068892848 | 0.000876566 | Up | PVR3-like protein |
| *Zm00001d005451* | 2.208363467 | 1.14297764 | 4.53E-11 | Up | Cellulose synthase A catalytic subunit 5 [UDP-forming] |
| *Zm00001d005535* | 2.121685203 | 1.085210618 | 2.93E-06 | Up | Aspartokinase |
| *Zm00001d005542* | 2.863385148 | 1.517721737 | 5.83E-14 | Up | Plastid phosphate/phosphoenolpyruvate translocator1 |
| *Zm00001d005624* | 3.299524416 | 1.722258094 | 1.04E-07 | Up | Delta(8)-fatty-acid desaturase 2 |
| *Zm00001d005793* | 2.058012132 | 1.041251487 | 0.014173085 | Up | Zein-beta |
| *Zm00001d005946* | 2.007446733 | 1.005361707 | 0.033932819 | Up |  |
| *Zm00001d006010* | 2.712099361 | 1.439410034 | 0.000769999 | Up |  |
| *Zm00001d006080* | 2.088764475 | 1.062649826 | 6.66E-11 | Up | Oleoyl-acyl carrier protein thioesterase |
| *Zm00001d006180* | 2.882471346 | 1.527306266 | 1.21E-05 | Up | NC domain-containing protein-related |
| *Zm00001d006293* | 2.988419494 | 1.579382678 | 0.000165638 | Up | NIN-like protein 1 |
| *Zm00001d006371* | 3.175946749 | 1.667186723 | 1.06E-07 | Up | C2 calcium/lipid-binding plant phosphoribosyltransferase family protein |
| *Zm00001d006608* | 2.176652127 | 1.122110854 | 0.002427424 | Up | Uncharacterized protein |
| *Zm00001d006705* | 2.026099841 | 1.018705268 | 0.03058671 | Up | ARM repeat superfamily protein |
| *Zm00001d006835* | 2.767941173 | 1.468813282 | 6.73E-28 | Up | Nuclear transcription factor Y subunit A-8 |
| *Zm00001d006866* | 3.012922035 | 1.591163341 | 0.000107827 | Up | 3-oxoacyl-[acyl-carrier-protein] synthase II chloroplastic |
| *Zm00001d006875* | 2.187599412 | 1.12934858 | 5.15E-08 | Up | Alanine aminotransferase10 |
| *Zm00001d007382* | 2.331112729 | 1.221018773 | 0.003621951 | Up | Transcription factor ICE1 |
| *Zm00001d008453* | 2.125356807 | 1.087705063 | 0.000706453 | Up |  |
| *Zm00001d008826* | 3.130661338 | 1.646467452 | 2.38E-29 | Up | Gigantea1 |
| *Zm00001d009146* | 2.472615749 | 1.306038059 | 0.000150997 | Up | S-adenosylmethionine synthase |
| *Zm00001d009374* | 2.220265878 | 1.15073245 | 0.011586946 | Up | DNA replication licensing factor MCM4 |
| *Zm00001d009439* | 2.561179193 | 1.356808195 | 1.26E-07 | Up | Putative CRAL/TRIO domain containing, Sec14p-like phosphatidylinositol transfer family protein |
| *Zm00001d009503* | 2.360630513 | 1.239172248 | 0.000132608 | Up | 2-oxoglutarate (2OG) and Fe(II)-dependent oxygenase superfamily protein |
| *Zm00001d009702* | 2.10423499 | 1.073295827 | 0.015478563 | Up | P-loop containing nucleoside triphosphate hydrolases superfamily protein |
| *Zm00001d009779* | 2.670006521 | 1.416843266 | 1.59E-11 | Up | Dihydrolipoyl dehydrogenases |
| *Zm00001d009811* | 2.428797643 | 1.280242296 | 6.08E-06 | Up | Protein networked 4A |
| *Zm00001d009888* | 2.240529954 | 1.163840015 | 0.001228583 | Up | DNA primase |
| *Zm00001d010018* | 2.022396162 | 1.01606563 | 7.53E-05 | Up | Protein kinase superfamily protein |
| *Zm00001d010178* | 2.659098895 | 1.410937434 | 5.32E-06 | Up | Protein kinase superfamily protein |
| *Zm00001d010193* | 2.190516649 | 1.13127118 | 0.000706453 | Up | Gibberellin-regulated protein 2 |
| *Zm00001d010371* | 2.016741389 | 1.012026096 | 0.033801312 | Up | Ribosome-inactivating protein 3 |
| *Zm00001d010578* | 2.050357231 | 1.035875291 | 1.80E-05 | Up | Protein kinase superfamily protein |
| *Zm00001d010689* | 2.543782054 | 1.346975069 | 8.04E-07 | Up | Cytokinin-O-glucosyltransferase 1 |
| *Zm00001d011183* | 2.240966736 | 1.164121234 | 1.53E-13 | Up | Thiamine biosynthesis1 |
| *Zm00001d011365* | 2.384028126 | 1.253401256 | 0.000288342 | Up | Alpha/beta-Hydrolases superfamily protein |
| *Zm00001d011486* | 2.784450156 | 1.477392468 | 2.77E-06 | Up | CTP synthase family protein |
| *Zm00001d011673* | 2.605447769 | 1.381531334 | 0.001022184 | Up | Farnesyl diphosphate synthase2 |
| *Zm00001d011712* | 3.009043286 | 1.589304861 | 8.47E-09 | Up | Putative B3 DNA binding domain family protein |
| *Zm00001d011846* | 3.352284951 | 1.745144786 | 1.18E-06 | Up | Calcium-dependent lipid-binding (CaLB domain) family protein |
| *Zm00001d011970* | 2.355240325 | 1.235874277 | 0.000470826 | Up | Probable protein S-acyltransferase 7 |
| *Zm00001d012033* | 4.035806084 | 2.012856856 | 1.73E-47 | Up | Stearoyl-acyl-carrier-protein desaturase2 |
| *Zm00001d012572* | 4.61559896 | 2.206517877 | 1.02E-09 | Up | Bifunctional inhibitor/lipid-transfer protein/seed storage 2S  Albumin superfamily protein |
| *Zm00001d012763* | 2.079917762 | 1.056526487 | 0.000409453 | Up | Kinesin-related protein16 |
| *Zm00001d013078* | 2.123657169 | 1.086550885 | 0.004137285 | Up | BAX inhibitor-1 family protein |
| *Zm00001d013222* | 2.577557338 | 1.366004521 | 3.72E-11 | Up | Gibberellin-regulated protein 10 |
| *Zm00001d013302* | 2.183752826 | 1.12680957 | 0.001578056 | Up | Auxin-responsive protein IAA14 |
| *Zm00001d013367* | 2.256247784 | 1.173925515 | 1.20E-06 | Up | Tubulin alpha-4 chain |
| *Zm00001d013461* | 2.014420229 | 1.010364676 | 0.014043811 | Up | Phospholipase A2-alpha |
| *Zm00001d013524* | 2.265875569 | 1.180068638 | 0.009258761 | Up | Polygalacturonate 4-alpha-galacturonosyltransferase |
| *Zm00001d013612* | 2.177630623 | 1.12275926 | 0.003411701 | Up | Tubulin beta-4 chain |
| *Zm00001d013703* | 3.429169752 | 1.777859323 | 1.52E-05 | Up | Serine/threonine-protein kinase Nek1 |
| *Zm00001d013873* | 2.760556551 | 1.464959155 | 1.19E-09 | Up | Actin-2 |
| *Zm00001d014496* | 2.12314775 | 1.086204772 | 0.014600533 | Up | ARM repeat superfamily protein |
| *Zm00001d014689* | 2.19243759 | 1.132535776 | 0.000359119 | Up | L-arabinokinase |
| *Zm00001d014814* | 2.273991617 | 1.185226936 | 0.000248409 | Up | Transcription factor bhlh93 |
| *Zm00001d015138* | 2.33269236 | 1.221996055 | 5.88E-06 | Up |  |
| *Zm00001d015780* | 2.44199702 | 1.28806144 | 6.59E-10 | Up | Protein WVD2-like 5 |
| *Zm00001d015912* | 2.658317757 | 1.410513565 | 0.001045415 | Up | Sugars will eventually be exported transporter4c |
| *Zm00001d016000* | 2.738781351 | 1.453534094 | 3.14E-29 | Up | MYB-related protein 3R-1 |
| *Zm00001d016660* | 2.920776021 | 1.54635173 | 0.000221191 | Up | Peptidyl-prolyl isomerase1 |
| *Zm00001d016662* | 2.140972642 | 1.098266361 | 0.004662085 | Up |  |
| *Zm00001d016684* | 2.60149837 | 1.379342804 | 0.000736677 | Up | 1,4-alpha-glucan branching enzyme |
| *Zm00001d017329* | 2.00038951 | 1.000280944 | 0.037112854 | Up | UDP-glycosyltransferase 82A1 |
| *Zm00001d017458* | 2.324414588 | 1.216867414 | 6.28E-11 | Up | Methylglutaconyl-CoA hydratase |
| *Zm00001d018080* | 2.599650218 | 1.378317522 | 1.02E-06 | Up | Probable beta-D-xylosidase 7 |
| *Zm00001d018128* | 2.097506419 | 1.068675226 | 0.000237499 | Up | Cyclin-D2-1 |
| *Zm00001d018331* | 2.453012651 | 1.294554674 | 0.002080473 | Up | Hsp70-Hsp90 organizing protein 2 |
| *Zm00001d018377* | 2.006163768 | 1.004439381 | 3.94E-05 | Up | Diphosphomevalonate decarboxylase MVD2 |
| *Zm00001d018631* | 2.458012439 | 1.297492217 | 0.000694197 | Up | Dirigent protein 11 |
| *Zm00001d018744* | 2.356542197 | 1.236671515 | 0.002564895 | Up | Putative cyclotide family protein |
| *Zm00001d018752* | 2.006572232 | 1.004733091 | 0.03588397 | Up | Leucine-rich repeat receptor-like serine/threonine-protein kinase BAM1 |
| *Zm00001d018883* | 2.62835714 | 1.394161321 | 8.51E-07 | Up | Probable inactive leucine-rich repeat receptor-like protein kinase |
| *Zm00001d018964* | 2.013705947 | 1.009853028 | 0.034382116 | Up | Guanylate-binding family protein |
| *Zm00001d018988* | 2.583395192 | 1.369268355 | 0.001614881 | Up | Associate of C-myc |
| *Zm00001d019250* | 2.037258844 | 1.026629293 | 0.00397213 | Up | Cytokinin-N-glucosyltransferase 1 |
| *Zm00001d019265* | 3.389438753 | 1.761046401 | 7.94E-06 | Up | UDP-glucosyltransferase |
| *Zm00001d020171* | 2.181579592 | 1.125373109 | 0.005520405 | Up | Leucine-rich repeat (LRR) family protein |
| *Zm00001d020172* | 2.160415543 | 1.111308833 | 2.96E-05 | Up | Probable LRR receptor-like serine/threonine-protein kinase |
| *Zm00001d020315* | 2.191023206 | 1.131604764 | 0.003207072 | Up |  |
| *Zm00001d020461* | 2.395373122 | 1.260250399 | 1.37E-08 | Up | Ankyrin protein kinase-like |
| *Zm00001d020592* | 3.839316797 | 1.940849608 | 3.67E-07 | Up | Glutelin-2 |
| *Zm00001d020771* | 2.164769566 | 1.114213462 | 2.08E-10 | Up | Glucose-6-phosphate isomerase |
| *Zm00001d020862* | 2.814420072 | 1.492837677 | 8.75E-09 | Up | Probable LRR receptor-like serine/threonine-protein kinase |
| *Zm00001d020929* | 2.036645578 | 1.026194941 | 0.003888815 | Up |  |
| *Zm00001d020984* | 2.724554291 | 1.446020239 | 5.03E-07 | Up | Probable sarcosine oxidase |
| *Zm00001d020986* | 2.654391731 | 1.408381297 | 2.00E-14 | Up | O-fucosyltransferase family protein |
| *Zm00001d021248* | 2.793309398 | 1.481975381 | 3.10E-12 | Up | NC domain-containing protein-related |
| *Zm00001d021433* | 2.193620795 | 1.133314152 | 0.000294957 | Up | High mobility group B protein 2 |
| *Zm00001d021442* | 2.759867104 | 1.464598799 | 3.30E-05 | Up | Protein NLP2 |
| *Zm00001d021558* | 2.28697338 | 1.193439573 | 1.30E-08 | Up | (S)-ureidoglycine aminohydrolase |
| *Zm00001d021654* | 2.048722667 | 1.034724702 | 0.003045533 | Up |  |
| *Zm00001d021813* | 3.370084804 | 1.752784895 | 3.11E-07 | Up |  |
| *Zm00001d021826* | 2.007373803 | 1.005309293 | 0.001676548 | Up | Nodulation signaling pathway 2 protein |
| *Zm00001d022062* | 2.141161201 | 1.098393415 | 6.67E-06 | Up |  |
| *Zm00001d022109* | 3.400179601 | 1.765610953 | 7.35E-07 | Up | Nuclear transcription factor Y subunit A-8 |
| *Zm00001d022144* | 2.997744514 | 1.583877433 | 2.98E-08 | Up | 3-oxoacyl-[acyl-carrier-protein] synthase II (chloroplastic) |
| *Zm00001d022274* | 2.026303908 | 1.018850568 | 0.023712215 | Up | Pyruvate orthophosphate dikinase4 |
| *Zm00001d022395* | 2.731391723 | 1.449636233 | 1.26E-06 | Up | Rhythmically expressed protein |
| *Zm00001d022421* | 2.597159557 | 1.376934649 | 0.000244878 | Up | Hydroxyproline-rich glycoprotein family protein |
| *Zm00001d022590* | 2.247882594 | 1.168566686 | 0.002606838 | Up |  |
| *Zm00001d022647* | 2.193597022 | 1.133298518 | 0.001519915 | Up | Armadillo repeat only 1 |
| *Zm00001d023462* | 2.453805134 | 1.295020684 | 0.000459827 | Up | Probable DNA primase large subunit |
| *Zm00001d023544* | 2.251807229 | 1.171083327 | 9.38E-24 | Up | Putative DUF869 domain containing family protein |
| *Zm00001d023801* | 2.02294042 | 1.01645383 | 0.012485981 | Up | DNA polymerase alpha 2 |
| *Zm00001d023899* | 2.668992046 | 1.416295006 | 3.78E-11 | Up | Peroxidase 24 |
| *Zm00001d024518* | 2.686707249 | 1.42583913 | 4.19E-18 | Up | Acyl-CoA-binding protein1 |
| *Zm00001d024939* | 2.081615724 | 1.057703765 | 0.000212022 | Up | Phosphoglycerate mutase-like protein |
| *Zm00001d024998* | 2.576942084 | 1.365660113 | 8.80E-24 | Up | Acetyl-coenzyme A carboxylase1 |
| *Zm00001d025103* | 2.094398352 | 1.066535867 | 2.23E-15 | Up | Amine oxidase1 |
| *Zm00001d025753* | 2.0448555 | 1.031998899 | 0.000595804 | Up | Chitinase B1 |
| *Zm00001d025818* | 4.474414908 | 2.161699042 | 2.72E-08 | Up | GDSL esterase/lipase LTL1 |
| *Zm00001d025834* | 4.98167326 | 2.316630401 | 1.12E-12 | Up | Inositol transporter 4 |
| *Zm00001d025924* | 2.088955625 | 1.062781846 | 0.00322408 | Up | (+)-Neomenthol dehydrogenase |
| *Zm00001d025979* | 2.015736403 | 1.01130699 | 0.033206949 | Up | Eukaryotic translation initiation factor 4G |
| *Zm00001d026109* | 2.06340987 | 1.045030423 | 0.000641172 | Up | Putative polyphenol oxidase family protein |
| *Zm00001d026591* | 2.2294087 | 1.156661119 | 0.000587361 | Up |  |
| *Zm00001d027281* | 3.87454368 | 1.954026409 | 2.81E-46 | Up | Expansin-B4 |
| *Zm00001d027332* | 2.143847049 | 1.100201981 | 9.48E-05 | Up | Nonspecific lipid-transfer protein |
| *Zm00001d027383* | 4.704424066 | 2.234018113 | 8.45E-16 | Up | Hydroxymethylglutaryl-CoA synthase |
| *Zm00001d027500* | 2.208875151 | 1.143311878 | 8.57E-05 | Up | Flower-specific gamma-thionin |
| *Zm00001d027539* | 2.190630974 | 1.131346473 | 0.000550283 | Up | Glutathione transferase11 |
| *Zm00001d027748* | 2.747877443 | 1.45831766 | 0.000586585 | Up | Probable RNA helicase SDE3 |
| *Zm00001d027760* | 2.308544263 | 1.206983394 | 0.001040481 | Up | Histone H2A |
| *Zm00001d027874* | 2.152749641 | 1.106180548 | 8.00E-05 | Up | Nuclear transcription factor y subunit a1 |
| *Zm00001d027996* | 2.146804896 | 1.102191084 | 6.15E-06 | Up | Alcohol dehydrogenase-like 2 |
| *Zm00001d028039* | 2.61244642 | 1.385401448 | 1.18E-06 | Up | Probable protein S-acyltransferase 22 |
| *Zm00001d028165* | 2.194793833 | 1.134085427 | 0.010848352 | Up | Aminotransferase ALD1 |
| *Zm00001d028258* | 2.054651235 | 1.038893525 | 0.026986681 | Up | Cytochrome P450 family 94 subfamily D polypeptide 2 |
| *Zm00001d029031* | 2.709148962 | 1.437839722 | 8.33E-06 | Up | Cytochrome P450 CYP714B3 |
| *Zm00001d029040* | 3.115968441 | 1.639680621 | 5.50E-17 | Up | Probable inactive receptor kinase |
| *Zm00001d029424* | 2.366863329 | 1.242976402 | 0.000401933 | Up |  |
| *Zm00001d030891* | 2.201735651 | 1.138641264 | 5.08E-06 | Up | Homeodomain-like superfamily protein |
| *Zm00001d030907* | 2.220548883 | 1.150916331 | 4.18E-07 | Up | B3 domain-containing protein |
| *Zm00001d030990* | 2.283182807 | 1.191046376 | 0.000460726 | Up | F-box/LRR-repeat protein 17 |
| *Zm00001d031005* | 2.205274205 | 1.140958052 | 0.004665813 | Up | Protein NETWORKED 1A |
| *Zm00001d031203* | 2.682180545 | 1.423406353 | 1.00E-09 | Up | Alpha/beta-Hydrolases superfamily protein |
| *Zm00001d031212* | 2.317058862 | 1.212294695 | 7.23E-08 | Up | Thiolase1 |
| *Zm00001d031253* | 2.083095668 | 1.058729098 | 4.99E-07 | Up | Dicarboxylic acid transporter2 |
| *Zm00001d031929* | 2.372890128 | 1.246645301 | 4.96E-08 | Up | Dihydroxy-acid dehydratase (chloroplastic) |
| *Zm00001d031988* | 3.019217742 | 1.594174806 | 1.59E-10 | Up | Transmembrane 9 superfamily member 9 |
| *Zm00001d032019* | 2.845571887 | 1.508718626 | 3.14E-18 | Up | Acyl carrier protein1 |
| *Zm00001d032036* | 2.077183636 | 1.054628765 | 0.005746518 | Up | Probable carboxylesterase 8 |
| *Zm00001d032274* | 2.794435391 | 1.482556819 | 0.000149364 | Up | Retinol dehydrogenase 13 |
| *Zm00001d032461* | 3.342212649 | 1.740803528 | 1.72E-08 | Up | Tasselless1 |
| *Zm00001d032464* | 2.46485042 | 1.301500099 | 2.96E-12 | Up | Kinesin-like protein KIN-7D mitochondrial |
| *Zm00001d032933* | 3.208649025 | 1.68196599 | 8.26E-10 | Up | Protein networked 1A |
| *Zm00001d032972* | 2.177042995 | 1.1223699 | 0.014801003 | Up | Pathogenesis-related thaumatin superfamily protein |
| *Zm00001d033048* | 2.051548432 | 1.036713213 | 0.011089568 | Up | TIFY8 |
| *Zm00001d033225* | 3.904819731 | 1.965255947 | 8.20E-09 | Up | Pyruvate dehydrogenase E1 component subunit beta-3 (chloroplastic) |
| *Zm00001d033280* | 2.983707114 | 1.577105925 | 0.000100821 | Up | IQ calmodulin-binding motif family protein |
| *Zm00001d033365* | 2.254341388 | 1.172706007 | 0.007746561 | Up | Putative cyclin-A3-1 |
| *Zm00001d033391* | 2.063775061 | 1.045285734 | 1.61E-07 | Up |  |
| *Zm00001d033405* | 4.247961126 | 2.086770564 | 5.61E-14 | Up | Pyruvate kinase |
| *Zm00001d033483* | 2.025503069 | 1.018280271 | 0.009911652 | Up | Dehydrin13 |
| *Zm00001d033559* | 2.322770224 | 1.215846445 | 0.000291043 | Up | Costars family protein |
| *Zm00001d033902* | 2.06508007 | 1.046197721 | 0.023707541 | Up | CAP (Cysteine-rich secretory proteins Antigen 5 and Pathogenesis-related 1 protein) superfamily protein |
| *Zm00001d034074* | 2.544398654 | 1.347324729 | 1.36E-38 | Up | Starch phosphorylase1 |
| *Zm00001d034076* | 3.367897605 | 1.751848276 | 1.07E-27 | Up | Cyclic nucleotide-gated ion channel 2 |
| *Zm00001d034117* | 2.43642575 | 1.284766257 | 2.46E-13 | Up | Actin-like ATPase superfamily protein |
| *Zm00001d034187* | 2.310831054 | 1.208411787 | 0.006389482 | Up | Thioredoxin-like protein HCF164 (chloroplastic) |
| *Zm00001d034191* | 2.585544408 | 1.370468084 | 3.44E-06 | Up | Electron transporter |
| *Zm00001d034257* | 3.217275948 | 1.685839682 | 1.67E-06 | Up | Proline-rich receptor-like protein kinase PERK4 |
| *Zm00001d034313* | 2.004963814 | 1.003576199 | 0.020837127 | Up | Reticulon-like protein B8 |
| *Zm00001d034387* | 2.004493175 | 1.003237505 | 0.000114432 | Up | Aldehyde oxidase1 |
| *Zm00001d034444* | 2.158493086 | 1.110024471 | 2.53E-06 | Up | Uncharacterized protein |
| *Zm00001d034480* | 2.014556475 | 1.01046225 | 5.96E-07 | Up | Protein WVD2-like 3 |
| *Zm00001d035161* | 2.817480437 | 1.494405593 | 0.000226418 | Up | Lysine histidine transporter 2 |
| *Zm00001d035559* | 2.036696845 | 1.026231257 | 8.87E-30 | Up | Dirigent protein |
| *Zm00001d035760* | 2.995271008 | 1.582686542 | 0.000139101 | Up | Zein protein 15kDa |
| *Zm00001d035776* | 2.001968259 | 1.001419101 | 0.010608685 | Up | Paired amphipathic helix protein Sin3-like 3 |
| *Zm00001d035899* | 2.879451875 | 1.52579421 | 3.03E-07 | Up | Pectin lyase-like superfamily protein |
| *Zm00001d036425* | 2.223195246 | 1.152634655 | 0.002324013 | Up | Silky1 |
| *Zm00001d036462* | 2.671281973 | 1.417532271 | 2.16E-10 | Up | ARM repeat superfamily protein |
| *Zm00001d036648* | 2.678243694 | 1.421287238 | 0.000743057 | Up | Nuclear transcription factor Y subunit C-2 |
| *Zm00001d036877* | 2.052170474 | 1.037150581 | 0.009375687 | Up | Phosphatidylcholine:diacylglycerol cholinephosphotransferase 1 |
| *Zm00001d036968* | 2.143773967 | 1.1001528 | 2.48E-12 | Up | Probable phospholipid-transporting ATPase 7 |
| *Zm00001d037097* | 2.193180696 | 1.13302468 | 0.001857394 | Up |  |
| *Zm00001d037108* | 2.061644911 | 1.043795871 | 0.000147296 | Up | 3-oxoacyl- |
| *Zm00001d037196* | 2.107135397 | 1.07528302 | 0.018423274 | Up | Retrovirus-related Pol polyprotein LINE-1 |
| *Zm00001d037234* | 2.61143078 | 1.384840463 | 1.24E-05 | Up | Sugary2 |
| *Zm00001d037333* | 2.194506231 | 1.133896366 | 1.13E-06 | Up | Ketose-bisphosphate aldolase class-II family protein |
| *Zm00001d037334* | 2.289702491 | 1.195160156 | 8.74E-08 | Up | Odorant 1 protein |
| *Zm00001d037384* | 2.106762413 | 1.075027626 | 0.020665937 | Up | Anthocyanidin 3-O-glucosyltransferase |
| *Zm00001d037436* | 3.20694923 | 1.681201514 | 4.89E-05 | Up | Zein delta 18 kDa |
| *Zm00001d037631* | 2.161572963 | 1.112081535 | 0.001064443 | Up |  |
| *Zm00001d037756* | 2.051600083 | 1.036749535 | 8.65E-06 | Up | Zinc transporter 4 precursor |
| *Zm00001d037816* | 3.076898101 | 1.621476666 | 1.24E-05 | Up | C4-dicarboxylate transporter/malic acid transport protein isoform 1 |
| *Zm00001d038269* | 2.049413188 | 1.035210879 | 0.025891387 | Up | UPF0503 protein chloroplastic |
| *Zm00001d038282* | 2.055601537 | 1.039560636 | 0.002268249 | Up | Putative LSTK-1-like/NimA-related protein kinase family protein isoform 1 |
| *Zm00001d038574* | 3.873503174 | 1.953638922 | 1.09E-09 | Up | Basic leucine zipper 24 |
| *Zm00001d038597* | 3.021559048 | 1.595293136 | 0.000145282 | Up | Globulin3 |
| *Zm00001d038891* | 2.244935729 | 1.166674142 | 0.00014009 | Up | Phosphoethanolamine N-methyltransferase 3 |
| *Zm00001d039105* | 2.444484645 | 1.289530343 | 2.94E-06 | Up | Putative MAP kinase family protein |
| *Zm00001d039425* | 2.112338999 | 1.078841384 | 8.84E-06 | Up |  |
| *Zm00001d039488* | 2.397565563 | 1.261570267 | 0.000517619 | Up | Pollen-specific protein SF21 |
| *Zm00001d039647* | 2.338010878 | 1.225281643 | 0.006262049 | Up | Boron transporter-like protein 2 |
| *Zm00001d039859* | 3.154473603 | 1.657399278 | 4.40E-61 | Up | Metallothionein-like protein type 2 |
| *Zm00001d040163* | 2.05904506 | 1.041975402 | 3.09E-05 | Up | Deoxy xylulose reductoisomerase1 |
| *Zm00001d040189* | 3.865949971 | 1.950822968 | 1.29E-06 | Up | No-apical-meristem-related protein1 |
| *Zm00001d040695* | 4.280371742 | 2.097736097 | 8.03E-21 | Up | PTI1-like tyrosine-protein kinase 3 |
| *Zm00001d040730* | 2.028372865 | 1.020322879 | 0.025305144 | Up | GDSL esterase/lipase LIP-4 |
| *Zm00001d041553* | 2.298379106 | 1.200616783 | 0.00012547 | Up | Osmotin-like protein |
| *Zm00001d041776* | 2.989759532 | 1.580029452 | 9.48E-05 | Up | Dhurrinase2 |
| *Zm00001d041962* | 3.183819558 | 1.670758574 | 6.36E-22 | Up | Glycerol-3-phosphate dehydrogenase [NAD(+)] 1 (chloroplastic) |
| *Zm00001d042084* | 2.004732208 | 1.003409534 | 5.37E-14 | Up | Villin-2 |
| *Zm00001d042344* | 2.336164312 | 1.224141749 | 6.85E-32 | Up | Stearoyl-acyl-carrier-protein desaturase1 |
| *Zm00001d042366* | 3.162628783 | 1.661124228 | 2.96E-09 | Up |  |
| *Zm00001d042470* | 2.143353314 | 1.099869686 | 1.71E-05 | Up | Protein kinase superfamily protein |
| *Zm00001d042555* | 2.051546977 | 1.03671219 | 0.00025926 | Up | Zinc-binding dehydrogenase family protein |
| *Zm00001d042658* | 2.06132727 | 1.043573575 | 0.001093467 | Up | Adenine nucleotide alpha hydrolases-like superfamily protein |
| *Zm00001d042961* | 2.148946761 | 1.103629742 | 0.000648818 | Up | Respiratory burst oxidase1 |
| *Zm00001d043147* | 2.256903035 | 1.174344436 | 0.000109839 | Up | FAD-dependent oxidoreductase family protein |
| *Zm00001d043265* | 2.048028385 | 1.034235711 | 0.023101864 | Up | LysM domain protein |
| *Zm00001d043443* | 2.628124587 | 1.394033668 | 8.40E-30 | Up | Mannan endo-14-beta-mannosidase 2 |
| *Zm00001d043504* | 2.3807826 | 1.251435888 | 7.12E-05 | Up | P-loop nucleoside triphosphate hydrolases superfamily protein with CH (Calponin Homology) domain |
| *Zm00001d043509* | 2.641619227 | 1.401422526 | 4.08E-12 | Up | Pectinesterase |
| *Zm00001d043601* | 2.663470143 | 1.413307109 | 5.62E-05 | Up | NADP-dependent malic enzyme6 |
| *Zm00001d043784* | 2.004961357 | 1.003574431 | 0.001079474 | Up | RING/U-box superfamily protein |
| *Zm00001d043938* | 2.453047487 | 1.294575162 | 0.000110333 | Up | Peroxidase 19 |
| *Zm00001d044202* | 2.793085328 | 1.481859648 | 0.000147327 | Up | Alpha/beta-Hydrolases superfamily protein |
| *Zm00001d044685* | 2.563523648 | 1.358128206 | 0.000499148 | Up | Non-specific lipid-transfer protein |
| *Zm00001d044686* | 2.051829172 | 1.036910622 | 5.79E-22 | Up | Non-specific lipid-transfer protein |
| *Zm00001d045027* | 2.492294235 | 1.3174744 | 1.04E-07 | Up | 3-oxoacyl-[acyl-carrier-protein] synthase I (chloroplastic) |
| *Zm00001d045192* | 2.095065867 | 1.066995602 | 0.00084132 | Up | Ribonucleoside-diphosphate reductase large subunit |
| *Zm00001d045302* | 2.088243105 | 1.062289674 | 0.01602522 | Up | Zea nodulation homolog1 |
| *Zm00001d045326* | 2.025052208 | 1.017959103 | 0.000152882 | Up | ATP-dependent 6-phosphofructokinase 3 |
| *Zm00001d045685* | 2.336967587 | 1.224637725 | 2.02E-06 | Up |  |
| *Zm00001d045873* | 2.452190207 | 1.294070888 | 0.000594184 | Up | Mannan endo-1,4-beta-mannosidase |
| *Zm00001d045940* | 2.632487731 | 1.396426807 | 0.000313548 | Up |  |
| *Zm00001d046444* | 2.419758818 | 1.274863259 | 5.63E-05 | Up | Fatty acid elongase2 |
| *Zm00001d046508* | 2.137246309 | 1.095753183 | 0.015517785 | Up | Phospholipase D5 |
| *Zm00001d046655* | 2.211770782 | 1.145201879 | 0.000686362 | Up | Putative STRUBBELIG family receptor protein kinase |
| *Zm00001d046866* | 2.158202892 | 1.109830499 | 1.26E-06 | Up | Senescence-associated protein DH |
| *Zm00001d046967* | 2.240692957 | 1.16394497 | 3.24E-05 | Up | Nuclear transcription factor Y subunit C-2 |
| *Zm00001d047054* | 3.445995118 | 1.784920658 | 1.46E-12 | Up | Pyruvate kinase |
| *Zm00001d047063* | 2.087003258 | 1.061432853 | 0.006450281 | Up | Camphor resistance CrcB family protein |
| *Zm00001d047096* | 2.338124989 | 1.225352054 | 0.004098931 | Up | Expansin-B4 |
| *Zm00001d047621* | 2.632241184 | 1.396291685 | 4.07E-05 | Up |  |
| *Zm00001d047764* | 2.240779712 | 1.164000827 | 0.010766353 | Up | Expressed protein |
| *Zm00001d048086* | 2.417629142 | 1.273592956 | 0.002106494 | Up | Replication protein A 70 kDa DNA-binding subunit B |
| *Zm00001d048099* | 2.903739314 | 1.53791194 | 8.31E-08 | Up | Beta-glucosidase 11 |
| *Zm00001d048476* | 2.143430259 | 1.099921477 | 0.008678408 | Up | Acyltransferase |
| *Zm00001d048634* | 2.26712698 | 1.180865198 | 3.71E-18 | Up | DIBOA-glucoside dioxygenase BX6 |
| *Zm00001d048702* | 2.591481435 | 1.373777059 | 3.16E-05 | Up | Benzoxazinone synthesis3 |
| *Zm00001d048703* | 2.169345757 | 1.117260012 | 0.007696924 | Up | Benzoxazinone synthesis4 |
| *Zm00001d048705* | 2.119379098 | 1.083641669 | 0.003882787 | Up | Benzoxazinone synthesis5 |
| *Zm00001d048731* | 2.598759531 | 1.377823144 | 0.0001028 | Up | P-loop containing nucleoside triphosphate hydrolases superfamily protein |
| *Zm00001d048850* | 2.071295777 | 1.050533582 | 0.020438855 | Up | Zein-alpha PMS1 |
| *Zm00001d048851* | 2.131614479 | 1.091946538 | 0.012614749 | Up | Floury4 |
| *Zm00001d048899* | 2.551002487 | 1.351064305 | 2.23E-10 | Up | HVA22-like protein a |
| *Zm00001d048901* | 2.088386451 | 1.062388704 | 4.78E-08 | Up | Transcription factor bhlh47 |
| *Zm00001d048979* | 2.191612923 | 1.131993016 | 1.51E-05 | Up | Putative sucrose-phosphate synthase family protein |
| *Zm00001d049085* | 3.282332139 | 1.714721232 | 1.20E-05 | Up | Putative laccase family protein |
| *Zm00001d049181* | 2.787513639 | 1.478978864 | 2.89E-08 | Up | O-methyltransferase ZRP4 |
| *Zm00001d049276* | 3.832683782 | 1.938354973 | 1.75E-09 | Up | Serine carboxypeptidase-like 46 |
| *Zm00001d049375* | 2.82406904 | 1.497775359 | 3.16E-05 | Up | Zinc transporter 3 |
| *Zm00001d049573* | 2.503272995 | 1.323815633 | 0.001873451 | Up | Cytochrome P450 family 87 subfamily A polypeptide 6 |
| *Zm00001d049753* | 2.084990261 | 1.060040645 | 3.16E-10 | Up | Sugary1 |
| *Zm00001d049975* | 4.551246932 | 2.186261863 | 4.88E-17 | Up | Enoyl-[acyl-carrier-protein] reductase [NADH] (chloroplastic) |
| *Zm00001d050100* | 2.309544545 | 1.207608372 | 0.001588287 | Up | Histone H2B.2 |
| *Zm00001d050178* | 2.103069029 | 1.072496204 | 0.022144904 | Up | Eukaryotic aspartyl protease family protein |
| *Zm00001d050340* | 2.702148853 | 1.43410715 | 0.000534191 | Up |  |
| *Zm00001d050484* | 2.688256869 | 1.426670997 | 2.93E-12 | Up | Transmembrane 9 superfamily member 9 |
| *Zm00001d050615* | 2.143765791 | 1.100147298 | 0.009412541 | Up | Zinc-finger domain of monoamine-oxidase A repressor R1 protein |
| *Zm00001d051157* | 2.057699231 | 1.041032122 | 0.009562668 | Up | Adenosine kinase 2 |
| *Zm00001d051313* | 2.45984265 | 1.298566033 | 0.002380047 | Up | Leucine-rich repeat (LRR) family protein |
| *Zm00001d051316* | 2.104579185 | 1.073531792 | 0.003480139 | Up | Putative homeodomain-like transcription factor superfamily protein isoform 1 |
| *Zm00001d051320* | 2.077839407 | 1.055084155 | 0.006640791 | Up | Esterase/lipase/thioesterase family protein |
| *Zm00001d051362* | 2.296873784 | 1.199671581 | 0.000210658 | Up | Tonoplast intrinsic protein2 |
| *Zm00001d051514* | 2.301552039 | 1.202607063 | 0.005275511 | Up | ABC transporter B family member 2 |
| *Zm00001d051636* | 2.649767394 | 1.405865721 | 6.53E-07 | Up | Delta(12)-fatty-acid desaturase |
| *Zm00001d051800* | 2.165995501 | 1.115030246 | 0.001477234 | Up | Caleosin protein |
| *Zm00001d051995* | 2.278082389 | 1.187819925 | 0.003328724 | Up | Proliferating cell nuclear antigen2 |
| *Zm00001d052389* | 2.754072694 | 1.46156664 | 0.000326606 | Up | Disease resistance protein RPM1 |
| *Zm00001d052405* | 4.692587115 | 2.230383528 | 1.38E-09 | Up | WRI1 transcription factor2 |
| *Zm00001d052893* | 2.118099981 | 1.082770691 | 0.003621951 | Up | Protein EMSY-LIKE 4 |
| *Zm00001d053109* | 4.345428252 | 2.119498365 | 1.14E-08 | Up | DUF724 domain-containing protein 2 |
| *Zm00001d053143* | 3.739747362 | 1.902940812 | 1.91E-06 | Up | Amino acid permease 2 |
| *Zm00001d053228* | 2.501885648 | 1.323015851 | 2.09E-22 | Up | Acyl carrier protein |
| *Zm00001d053395* | 2.018845595 | 1.013530575 | 0.030741322 | Up | Pectin lyase-like superfamily protein |
| *Zm00001d053396* | 2.032010887 | 1.022908132 | 1.38E-07 | Up | Abscisic acid receptor PYL9 |
| *Zm00001d053438* | 2.00964865 | 1.006943295 | 3.77E-07 | Up | Putative heavy metal transport/detoxification superfamily protein |
| *Zm00001d053643* | 2.036999116 | 1.026445354 | 0.000548359 | Up | Seed specific protein1 |
| *Zm00001d053659* | 2.265593244 | 1.179888868 | 0.000648818 | Up | Calcium ion binding protein |
| *Zm00001d053722* | 2.142942109 | 1.099592877 | 0.003159175 | Up | Cyclase |
| *Zm00001d053998* | 2.185483586 | 1.127952543 | 0.013371862 | Up | Putative wee1-like protein kinase |
