# Supplementary material for: Transcriptome and co-expression network analyses of key genes and pathways associated with differential abscisic acid accumulation during maize seed maturation
Source: BMC Plant Biol. 2022 Jul 22;22:359. doi: 10.1186/s12870-022-03751-1 (PMC9308322; doi:10.1186/s12870-022-03751-1)
Supplement: Supplementary file 5 — Additional file 5: Table S5. Gene-specific primers used in RT-qPCR. [file 12870_2022_3751_MOESM5_ESM.docx]

**Supplemental Table** **5** Gene-specific primers used in RT-qPCR.

| **Gene ID** | **Forward sequence** | **Reverse sequence** |
| --- | --- | --- |
| *Zm00001d021946* | TGCTGCTTTCCTTCATCTCC | CGAAGAACCTACGGGGATTG |
| *Zm00001d003648* | GAAAGGGAGGACATAACGA | AAGTTCAGCTCCATCCGTGT |
| *Zm00001d035560* | ACGACCCTACCTACCTTTGA | CATAGAACCCCACGATGCTG |
| *Zm00001d012043* | CAATGTGGGTGGTGATTGGA | AAGGTGCTACTGGTCTCTGT |
| *Zm00001d044747* | CAAGACGTTTAGGTGCTGCT | ATGTCCCGTTCATGCTCAAG |
| *Zm00001d027996* | AACATCGTCAGGGGGTTAGA | CACTTTCATCTGTGCCCTCT |
| *Zm00001d028474* | GTATCAGAGGCACATCCAGC | GAATGGGTGGCTTGTTACCT |
| *Zm00001d028391* | GGGACTGCCGATAAAGATG | CACACACCACTGTCACTACC |
| *Zm00001d043516* | ATGGGGAACCTGAAAGACG | GGTCAGAACGACGAGCAAA |
| zm00001d044228 | ATCTGGGACTTTGTTGTTGTTG | CTACACGAACACGACACACTAA |
| *zm00001d017666* | CTACATCGCGCTGTACATGATC | CGAGGTAGAAGTAGCTGAAGAA |
| *zm00001d031785* | GTCAACAACGTGCTCTTCAAC | CAGATGTACTGCTTGGTCTGG |
| *zm00001d008875* | GGAAGGAGGGTTAGTGCAGT | GCCATCTTCCCATTGTCGTC |
| *zm00001d027332* | GTGGTTGTGTCACTTTTCTGTT | ACAACACGACACGACTATAGAG |
| *zm00001d013612* | AATGTCAAGTCCAGCGTGTG | GGCCTCAGTGAACTCCATCT |
| *zm00001d047597* | CATGATGGCCAAGTGAGTAAAG | TTGCCTTACTAATATGCAACGC |
| *Zm00001d016438* | TTCCCAAGGTCTCTAAGGTTTC | ATTAGAGAAGTCGATCGCCTTT |
| *Zm00001d015477* | CAAGAACGAGGCTCTGAACG | TCTTCACGAACCTCTCCGAC |
| *Zm00001d012407* | GAATTCACCAACCAAACTCCAC | GAAGAACTTCCTGGAAGCCATT |
| *Zm00001d017121* | CAAAGTGCTTCCTGTCCTTAAC | TTGCTGACTTTTCAAGCCTAAC |
| *Zm00001d010588* | CAACGCCATCCATAACTCCG | CTTGCTCGTGTCGTCCTTG |
| *Zm00001d018414* | AGCAAAGCAAGAAGACAGTTG | CTGCTGGTCGATCGTCAT |
| *Zm00001d002025* | CTTCCATCTACCGAGGTGTAAC | ATTATGGTCTCCACGTTGTACC |
| *Zm00001d010193* | CAGCTTTGCCAAAAAGACATGA | CGCGCACAATATAATGAACCAA |
| *zm00001d039859* | CGAGGAAGAAGGATGTCGTG | AACACCCATGATGAGAGTCTG |
| *ZmUBI* | TAAGCTGCCGATGTGCCTGCG | CTGAAAGACAGAACATAATGAGCACAG |
