# Supplementary figures and images for: Transcriptome and co-expression network analyses of key genes and pathways associated with differential abscisic acid accumulation during maize seed maturation
Source: BMC Plant Biol. 2022 Jul 22;22:359. doi: 10.1186/s12870-022-03751-1 (PMC9308322; doi:10.1186/s12870-022-03751-1)

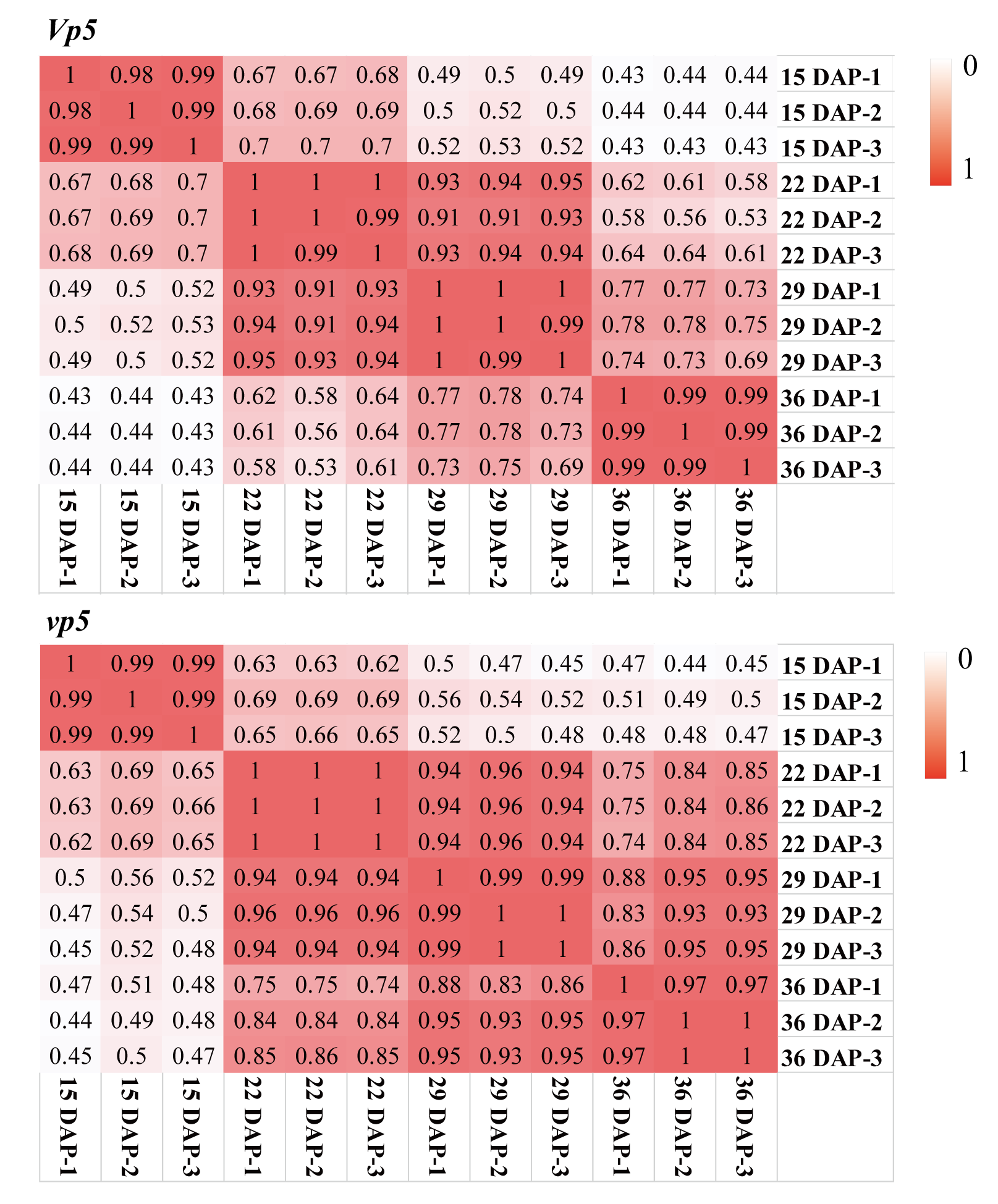

Supplement: Supplementary file 6 — Additional file 6: Fig. S1. Heat map of correlation coefficient between Vp5 and vp5 samples. [file 12870_2022_3751_MOESM6_ESM.tif]

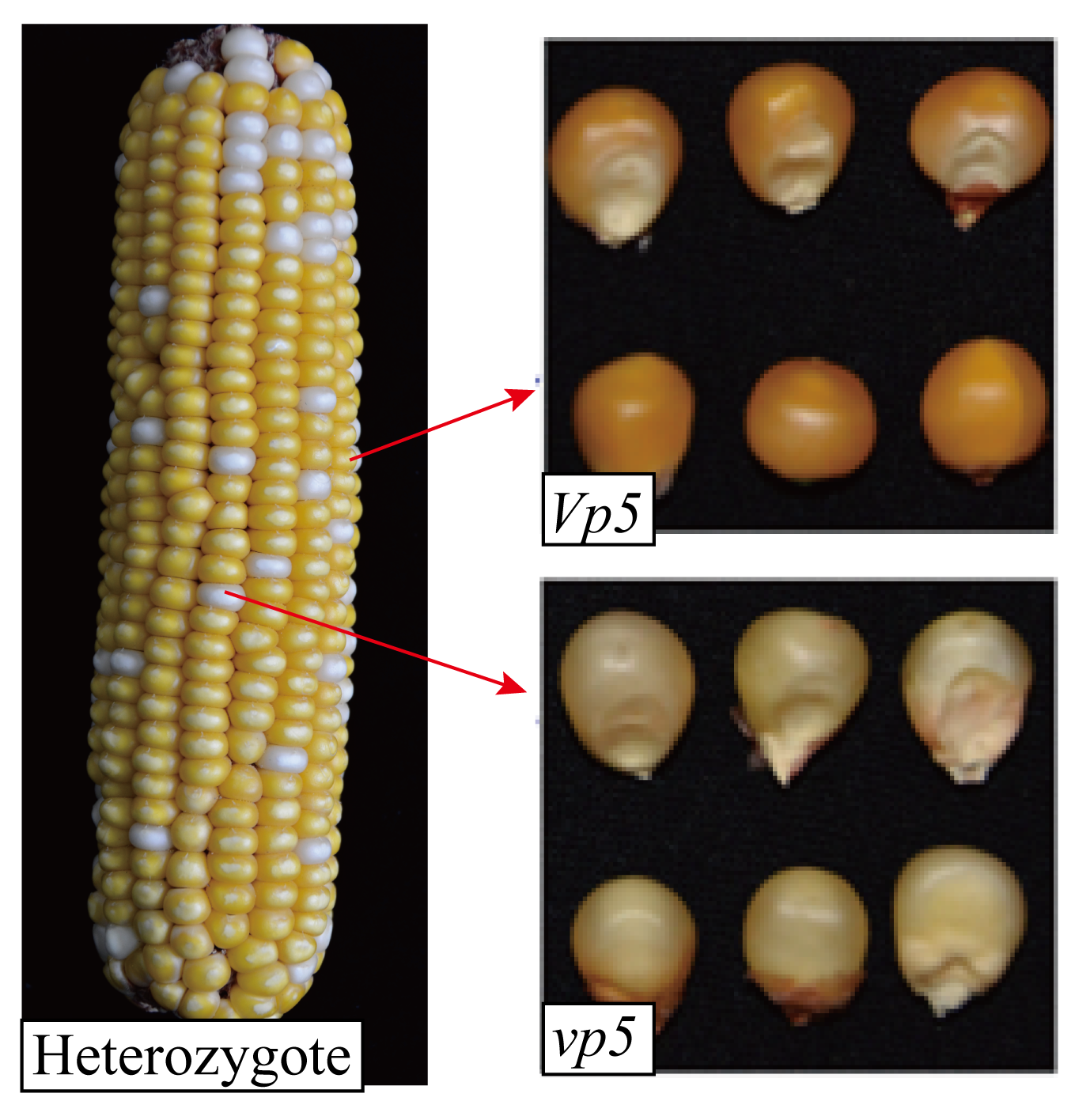

Supplement: Supplementary file 7 — Additional file 7: Fig. S2. A mature ear with white vp5 kernels and yellow Vp5 kernels separating from the mutation. [file 12870_2022_3751_MOESM7_ESM.tif]
